# Review and redescription of species in the *Oecetis avara* group, with the description of 15 new species (Trichoptera, Leptoceridae)

**DOI:** 10.3897/zookeys.376.6047

**Published:** 2014-02-03

**Authors:** Roger J. Blahnik, Ralph W. Holzenthal

**Affiliations:** 1 Department of Entomology, University of Minnesota, 1980 Folwell Ave., 219 Hodson Hall, St. Paul, Minnesota, 55108, U.S.A.

**Keywords:** Caddisfly, Neotropics, Nearctic, key, species complex, COI barcode

## Abstract

The *O. avara* group of *Oecetis* is formally defined to include 4 described species, *O. avara* (Banks), *O. disjuncta* (Banks), *O. elata* Denning & Sykora, and *O. metlacenis* Bueno-Soria, and 15 new species. *Oecetis marquesi* Bueno-Soria, previously considered a member of the *O. avara* group, is treated as *incertae sedis* to species group, but is also redescribed and treated in the current work. New species described here (with their respective distributions) include: *O. acciptrina* (Costa Rica, Panama, Ecuador), *O. agosta* (Mexico), *O. angularis* (Guatemala to Ecuador), *O. apache* (SW USA), *O. campana* (Ecuador), *O. constricta* (Mexico to Ecuador, Venezuela, and Trinidad), *O. houghtoni* (North America), *O. maritza* (Costa Rica), *O. mexicana* (Mexico to Ecuador), *O. patula* (Guatemala, Nicaragua), *O. protrusa* (Mexico to Ecuador), *O. sordida* (Mexico, USA, Canada), *O. tumida* (Costa Rica), *O. uncata* (Costa Rica), and *O. verrucula* (Mexico to Costa Rica). A key to the species is also provided.

## Introduction

The genus *Oecetis* is the largest in the family Leptoceridae, with nearly 500 species worldwide (over 500 with the publication of this paper). The genus has gone through a dramatic and relatively recent expansion in known species diversity, especially with the publications by Schmid (1995a, 1995b, 1995c) on species from India; Malicky (2005, 2006, and numerous miscellaneous publications) on species from Asia; Mey (1995, 1998a, 1998b, 2003) on species from the Philippines; Yang and Morse (2000) on species from China; Wells (2000, 2004, 2006) on species from Australia, and Randriamasimanana and Gibon (1998, 1999a, 1999b, 2000) on species from Madagascar. Almost half of the known species in the world have been described since 1995. From the New World, only 55 species of *Oecetis* are currently recognized, 25 recorded from the Nearctic region and 37 from the Neotropics. Morse (1993) provided a checklist of the species for the entire Nearctic region, including Mexico, and Poole and Gentili (1996) provided an updated checklist of the species from North America north of Mexico. Neotropical species were catalogued by Flint et al. (1999). The most recently described *Oecetis* from the New World are 1 species described from Haiti (Flint and Sykora 2004), 7 from Bolivia and Argentina, described by Angrisano and Sganga (2009) and Rueda Martín et al. (2011), and 1 from Brazil by Quinteiro and Calor (2012). The work by Rueda Martín et al. (2011) also included a key for the known Neotropical species.

One difficulty encountered in the taxonomy of New World *Oecetis* is the existence of species complexes of closely related species, a problem undoubtedly due to the structural simplicity and conservatism of features of the male genitalia in some species groups. One known species complex in North America (but also extending into South America) is that of *Oecetis inconspicua* (Floyd 1995, Zhou et al. 2011). The taxonomy of this group, which may ultimately prove to contain a significant percentage of the species diversity of North American *Oecetis*, has still not been resolved. The *Oecetis avara* group, as treated here, is another such species complex.

The only comprehensive treatment of the genus *Oecetis* as a whole is that of Chen (1993), a dissertation treatment that was unfortunately never published and thus is not readily available. As noted above, about half of the diversity in the genus has been described since then, making a reassessment already desirable. Chen recognized 4 subgenera in *Oecetis*, all of them found in the New World. One of these was the subgenus *Pseudosetodes* Ulmer, 1905, comprised of 7 species groups distributed in the Old and New Worlds and also Australia. Evidence for monophyly of the subgenus was primarily based on a male genitalic character: males with “a symmetrical phallus lacking parameres (internal spines).” The *Oecetis avara* and *Oecetis punctata* groups are 2 New World endemic groups that were assigned to the subgenus and considered by Chen to be sister taxa. Based on our own examination of the included species, we agree with the monophyly and probable sister relationship of the 2 species groups. In addition to having an upright lobe on the inferior appendages, species of both groups are very similar in appearance, particularly in color and forewing spotting. The *Oecetis punctata* group is confined to the Neotropics and the *Oecetis avara* group is found in both the Nearctic and Neotropical regions. As recognized by Chen, the *Oecetis punctata* group included only 3 described species: *Oecetis punctata* (Navás, 1924); *Oecetis silviae* Bueno-Soria, 1981; and *Oecetis knutsoni* Flint, 1981. However, one of the species subsequently described by Rueda Martín et al. (2011), *Oecetis chipiriri*, also belongs here. Additional, undescribed species are known in the collections of the Smithsonian and University of Minnesota. Like the *Oecetis avara* group, species within the *Oecetis punctata* group seem to be quite variable and will probably be found to constitute a complex of closely related species. Thus, this group is also in need of a revision.

The *Oecetis avara* group contains only 4 previously described species: *Oecetis avara* (Banks, 1895), *Oecetis disjuncta* (Banks, 1920), *Oecetis elata* Denning & Sykora (1966), and *Oecetis metlacensis* Bueno-Soria (1981). *Oecetis marquesi* Bueno-Soria, 1981, was also considered a member of the *Oecetis avara* group by Chen (1993), but is treated here as *incertae sedis* to species group. It is, however, redescribed and treated in the current paper, along with a discussion of the reasons for its transfer. Of species included in the *Oecetis avara* group, *Oecetis avara* itself has by far the widest recorded distribution. However, as outlined below, much of the material previously assigned to *Oecetis avara* actually represents members of a species complex.

The immediate incentive for the current revision was the collection by the second author of a number of putative species belonging to the *Oecetis avara* group from his work in Costa Rica. As a significant result of this study, 10 species in the *Oecetis avara* group are now known from Costa Rica, including a remarkable 9 new species! It was recognized from the outset that these forms were morphologically very similar and that a more comprehensive study of material from throughout the Neotropics would be valuable in assessing their variability. Consequently, a standard revision was originally planned and initiated by the first author, focusing on the Neotropical material. Because the North American fauna has been known for a long time, we did not originally believe that it was necessary to include all of the extensive material potentially available from North America, but instead tried to determine which, if any, of the Neotropical species belonged to *Oecetis avara*, the common North American species. The holotype of *Oecetis avara* was examined and representative material of the species was borrowed from the Smithsonian, to complement additional material available in the University of Minnesota collection. It turned out that North American material assigned to *Oecetis avara* was much more variable than expected. Moreover, the variability was difficult to assess. Since a complete revision of North American material identified as *Oecetis avara* was beyond the scope of the original project, we limited the eventual study to a review and revision of the Neotropical species in the *Oecetis avara* group. However, several new species in the *Oecetis avara* group are also recognized and described from North America, based on the North American material examined. Despite constituting an incomplete revision, all of the described species of the *Oecetis avara* group have been reillustrated, including types of the Nearctic species, *Oecetis avara* and *Oecetis disjuncta*.

The difficulties presented by variable populations of *Oecetis avara* in the Nearctic was evident already to Ross (1944), who noted the differences in forewing spotting and genitalia in the species, and even suggested that *Oecetis disjuncta* may not be a species distinct from *Oecetis avara*. Denning (1956) expressed the same opinion. Smith and Lehmkuhl (1980) devoted an entire paper to distinguishing *Oecetis avara* from *Oecetis disjuncta* (males, females, larvae, and pupae) and demonstrated that these were different species. However, a great deal of variation remained in the form identified by Smith and Lehmkuhl (1980) as *Oecetis avara*, and we have concluded that at least 1 additional species is represented. The populational differences in this material is much more subtle and difficult to interpret than those that separate these forms from *Oecetis disjuncta*. Because of the difficulty in assessing this variation, we limited our objectives in this paper to critically defining characters found in new and previously described type specimens of North American species. This allowed us to identify populations when they strictly conformed to characters found in the types, but left some variation unaccounted for. Eventually it may be found that this variation falls within existing species, or it may represent as yet unrecognized additional species. We hope the current paper will serve as a stimulus for additional taxonomic and population studies of the North American taxa. Some remaining taxonomic problems, as currently evident to us, are included in a short section following the species descriptions.

Compared to the variability in Nearctic populations of *Oecetis avara*, the species from Central and South America are much more morphologically stable. This is despite the relatively minor differences that seem to separate them. Almost all of the species recognized here are remarkably constant in morphology, some over rather extensive distributions. This contrasts to the previous perception of *Oecetis avara* being a very variable species. Additionally, a number of species occur together in sympatric populations, without intergrading variation, thus confirming their species status. A list of species known to occur in sympatry (in the strict sense of occurring together at the same site) can be found in Table 1. In addition to their genitalic differences, many of the Neotropical species can also be sorted without reference to male genitalia (with experience), based on slight differences in color, forewing spotting, and size. However, often a series of pinned specimens is required to appreciate these subtle differences. A key is provided to all species in the *Oecetis avara* group, in addition to detailed diagnoses, descriptions and illustrations of males of new and previously described species.

**Table 1.** Species of the *Oecetis avara* group occurring in sympatry.

	1	2	3	4	5	6	7	8	9	10	11	12	13	14	15	16	17	18	19
1. *Oecetis acciptrina*	–						×												
2. *Oecetis agosta*		–											×						
3. *Oecetis apache*			–													×			
4. *Oecetis angularis*				–			×					×			×				
5. *Oecetis avara*					–					×									
6. *Oecetis campana*						–							×						
7. *Oecetis constricta*							–												×
8. *Oecetis disjuncta*								–											
9. *Oecetis elata*									–										
10. *Oecetis houghtoni*										–						×			
11. *Oecetis maritza*											–								
12. *Oecetis metlacensis*												–			×				
13. *Oecetis mexicana*													–		×		×		×
14. *Oecetis patula*														–					
15. *Oecetis protrusa*															–		×		
16. *Oecetis sordida*																–			
17. *Oecetis tumida*																	–		
18. *Oecetis uncata*																		–	
19. *Oecetis verrucula*																			–

## Methods

Methodology follows that discussed by Blahnik and Holzenthal (2004) for caddisflies in general. Genitalia of most specimens were cleared in 10% KOH at room temperature for 6-12 hours. Toward the end of the study, some specimens were cleared in 85% lactic acid, as discussed by Blahnik et al. (2007). Terminology for male genitalia follows that used by Morse (1975) for Leptoceridae, and as indicated in Fig. 5 for genitalic characters peculiar to the *Oecetis avara* group. Terminology for wing veins follows that of Schmid (1998), and is indicated in Fig. 33. Illustrations were drawn with use of an ocular grid, scanned, and drawn using Adobe Illustrator (Holzenthal 2008).

Each pinned specimen, or lot of specimens in alcohol, examined during the study, was affixed with a barcode label (4 mil polyester, 8 × 14 mm, code 49) with a unique alphanumeric sequence preceded with the prefix UMSP. The prefix is not meant to imply ownership by the University of Minnesota Insect Collection (UMSP), but only to indicate that the specimen was databased in that collection. Specimen taxonomic and collection data are stored in Biota (v3.0) [http://viceroy.eeb.uconn.edu/Biota/]. Specimen barcode label information is included for holotypes in the list of material examined. A detailed list of all material examined, including individual barcode numbers, is maintained at UMSP and can be provided on request.

Females of the various species are not described because, for the most part, they are morphologically very similar and consistent diagnostic characters to distinguish the species were not found. Females listed in the paratype series and material examined lists are presumptively identified by association with their male counterparts.

Habitus photographs for the various species are provided in Figs 35–57 and reflect the general differences in forewing spotting, color, and size among the species. Wing photographs are at the same relative scale to make comparisons more direct. It should be noted, however, that there is often significant size variation within species, as well as some variation in color and forewing spotting.

Distribution maps (Maps 1–5) for the species are available as supplemental. kmz files, and can be downloaded from the journal’s website [see Appendix] and opened in Google Earth [http://www.google.com/earth/index.html]. These were created using the application provided by Earth Point [http://www.earthpoint.us/ExcelToKml.aspx] to convert Microsoft Excel files to. kml or. kmz files. Geographic coordinates for specimens that did not have specific latitude and longitude data on the label (see the material examined list for the individual species) were estimated using Acme Mapper 2.0 [http://mapper.acme.com] or Google Earth. Although localities were mapped as accurately as possible, the coordinates for some of these specimens is approximate; they are included to indicate the general distribution of the species. The reader is advised to deselect the otherwise default “other placemarks” in Google Earth (names of landmarks, cities, etc.), so as not to obscure or compete with the individual species distribution symbols.

Representatives of over half the species included in this monograph were submitted to the Barcode of Life (BoL/BOLD) initiative, Guelph, Ontario, Canada [http://www.barcodinglife.org/]. Only a single leg of a specimen was submitted for DNA extraction, with the remainder of the specimen and genitalia retained as a voucher and stored in the University of Minnesota Insect Collection (UMSP). Not all of the specimens or species included in the study produced sequence data and some COI barcode sequences obtained were only partial sequences. A K2P neighbor joining (NJ) phylogram of the sequences obtained (the default method of analysis made available by the BoL site) is included in Fig. 58 and is provided as ancillary data. The identification numbers associated with the specimens from Minnesota in the phylogram are their UMSP database barcode labels, as discussed above, and not their BoL processing IDs.

Holotypes are deposited in the collections of the University of Minnesota, St. Paul, Minnesota, USA (UMSP) and the National Museum of Natural History, Smithsonian Institution, Washington DC, USA (NMNH), as designated in the species descriptions. Paratypes are deposited in the same institutions, and also in the Instituto Nacional de Biodiversidad, Heredia, Costa Rica (INBIO) and the Instituto de Zoología Agrícola, Universidad Central de Venezuela, Maracay, Venezuela (IZAM).

### Character systems used in diagnosing species

Because the differences between species are subtler than those ordinarily used to diagnose species of *Oecetis*, some discussion of the character systems useful in distinguishing species is warranted. Of these, the shape of the phallobase is perhaps the single most useful character. It varies in length, angularity of the ventral surface, and often in the shape of the apex, as viewed caudally. The second most useful character is the shape of the inferior appendage. In all species of the *Oecetis avara* group, the inferior appendage has distinct dorsal and ventral lobes and an overall shape that can be described as mitten-like. The relative development of the 2 lobes varies among species, perhaps reaching its most polar variation in *Oecetis sordida* sp. n. (formerly included in *Oecetis disjuncta*) and *Oecetis avara*. In *Oecetis sordida*, the dorsal lobe is well developed and the ventral lobe short, demarcated from the dorsal lobe by hardly more than a rounded notch (Fig. 23A). In *Oecetis avara*, the dorsal lobe is less prominent, but the ventral lobe is well developed and demarcated from the dorsal lobe by an angular projection (Fig. 5A). The angle formed between the dorsal and ventral lobes, in those species in which the lobe is angularly projecting, varies from obtuse (as in *Oecetis constricta* sp. n., Fig. 7A), to acute (as in *Oecetis avara*, Fig. 5A, *Oecetis verrucula* sp. n., Fig. 26A, and *Oecetis protrusa* sp. n., Fig. 22A). In general, the more prominently the ventral lobe is developed, the more acute the articulation. An additional character useful in distinguishing some species is that the ventral lobes of the inferior appendages, as viewed ventrally, each have an angular bend on the mesal surface that causes the apices of the lobes to be either slightly diverging, as for example in *Oecetis agosta* sp. n., *Oecetis constricta*, sp. n., or *Oecetis verrucula*, (Figs 2E, 7F, and 26E), or more angularly diverging, as in *Oecetis angularis* sp. n. or *Oecetis protrusa* sp. n. (Figs 3E and 22E). In some species, as for instance *Oecetis avara*, this character is either intermediate or not consistent enough to be useful.

In general, there seems to be a correlation between the shape of the inferior appendages and the angularity of the ventral surface of the phallobase, as viewed laterally. Species with a weakly demarcated (notched or weakly protruding) ventral lobe of the inferior appendages usually have the ventral surface of the phallobase distinctly angled (as opposed to being merely arched). These species also lack an asymmetrical apical phallic sclerite, a character first noted by Smith and Lehmkuhl (1980) to distinguish *Oecetis disjuncta* from *Oecetis avara*. Species with this combination of characters include *Oecetis acciptrina* sp. n., *Oecetis apache* sp. n., *Oecetis disjuncta* (Banks), *Oecetis elata* Denning & Sykora, *Oecetis metlacensis* Bueno-Soria, *Oecetis sordida* sp. n., and *Oecetis uncata* sp. n. These species, however, differ greatly in overall size and coloration. The opposite character combination would be for the dorsal and ventral lobes of the inferior appendages to be angularly demarcated (ventral lobe with an angular projection) and for the phallobase to be arched ventrally, as viewed laterally, rather than very angularly bent. Additionally, an asymmetrical phallic sclerite is present in many of the species. This combination of characters is exemplified in *Oecetis avara*, *Oecetis houghtoni* sp. n., *Oecetis protrusa* sp. n., and *Oecetis mexicana* sp. n., among others.

The last important character system includes the overall habitus, affected by the relative size, coloration, and forewing spotting of the individual species. This is best appreciated in pinned material. The pigmentation of the forewing spots is located on the veins and sometimes adjacent wing membrane and generally not the overlying setae. Thus the spotting is evident even in alcohol preserved material in which the setation is lost. Spots are generally absent or indistinct in teneral specimens (which are frequently collected). Color varies from light yellow (stramineous) to dark brown. *Oecetis mexicana* sp. n. and *Oecetis protrusa* sp. n. are among the palest colored species in the group. Because it is a widespread and common species, we have used *Oecetis mexicana* sp. n. as a standard of comparison in describing species that are only slightly darker or lighter in color. Forewing spotting varies from absent or indistinct to the presence of very distinct spots at the intersection of major veins and crossveins, or at the terminus of major veins in some species, usually with the largest spots at the base of the discal and thyridial cells and the base of fork V (Fig. 33A). Some variation in these characters occurs within, as well as between species, although usually to a much lesser extent. The spacing of the crossveins in the forewing that constitute the chord is another character that is useful in diagnosing some species. The chord is constituted by the *s*, *r-m*, and *m* crossveins. Polar character states are illustrated in Figs 33A and 34A. The spacing often varies within species, with the crossveins being either more or less equally spaced, or (more typically) with the *s* and *r-m* crossveins somewhat closer. In species with the crossveins narrowly separated, they may appear either diagonal (Fig. 34A), or the *s* and *r-m* crossveins may be more closely spaced, sometimes almost (but not quite) linear. In species with the crossveins more distantly separated, they appear either evenly “stepped” (Fig. 33A), or with the space between the *r-m* and *m* crossveins distinctly wider. Species typified by one or the other configuration may have individuals that vary, due to the relatively large degree of variation within species. Size varies considerably in the *Oecetis avara* group, from small in species like *Oecetis mexicana* sp. n., *Oecetis constricta* sp. n., or *Oecetis elata* Denning & Sykora (forewing length 6.5–9.0 mm), to relatively large in species like *Oecetis sordida* sp. n. (forewing length 10.8–12.7 mm).

### *Oecetis avara* group

The most diagnostic aspect of this species group, and the strongest indicator of its monophyly, is the shape and structure of the inferior appendage of the male, which has a characteristic, somewhat mitten-like form. In all of the species, the dorsal lobe of this appendage has stout, thickened downward-curved setae on its mesal surface. The asymmetrical phallic sclerite, used by Smith and Lehmkuhl (1980) to differentiate *Oecetis avara* from *Oecetis disjuncta* is present in several species of the group, most of them newly described here; when present it is usually present on the right side, but may be present on the left side instead, even within the same species. Even in species where it is normally present, the sclerite may not always be evident, either because it is small or weakly projecting (or possibly it is absent in some individuals).

Although consistent characters for identifying females of *Oecetis avara* group species were not found, the range of variability likely to be encountered is included in the illustrations of *Oecetis avara* and *Oecetis disjuncta* by Smith and Lehmkuhl (1980). However, as noted below, there is some uncertainty about exactly which species were illustrated by Smith & Lehmkuhl. Females are most reliably associated with their male counterparts by similarities in color and forewing spotting. Usually females have wings that are shorter and slightly darker in color than males. Where several species co-occur, females of similar coloration can often be sorted by relative size differences, but this may not always be completely reliable.

The lighter colored and distinctly spotted species of the *Oecetis avara* group, such as *Oecetis mexicana* sp. n. and *Oecetis protrusa* sp. n., are very similar in color and general appearance to members of the *Oecetis punctata* group. In these *Oecetis avara* group species, much of the color in the apical part of the forewings comes from elongate, diverging, semi-prostrate setae emerging from the wing veins. Setation of the forewing membrane is relatively short and scant, with the membrane itself clear and semi-iridescent beneath. Other species in the *Oecetis avara* group, such as *Oecetis disjuncta*, *Oecetis sordida* sp. n., and *Oecetis apache* sp. n., have the forewings more uniformly setose and the setation on the membrane in the apical part of the wing veins of the forewing more or less apically directed, and thus the laterally splayed setae on the veins may be less evident.

In addition to the general shape of the inferior appendages, specimens in the *Oecetis avara* group, can generally be distinguished from specimens in the *Oecetis punctata* group by the spacing of the crossveins in the chord of the forewing. In the *punctata* group the outer 2 crossveins (the *s* and *r-m* crossveins) are linearly arranged, or almost so, and distant from the *m* crossvein. In the *Oecetis avara* group these same cross veins are usually distinctly staggered, although often somewhat closer to each other than to the *m* crossvein.

*Adult*. Crossvein *r* absent (as in *Oecetis* generally). R_1_ weakly developed and indistinct, except at apex. Chord with *s*, *r-m*, and *m* crossveins distinctly spaced, either narrowly (Fig. 34A) or more widely (Fig. 33A); *s* and *r-m* typically more closely spaced (Fig. 33A). Wing color ranging from yellow (testaceous) to dark brown (lighter color predominating). Chord of forewing, except in unmarked or weakly marked species, either pigmented or with small, dark spots at intersection of crossveins with major veins, spots sometimes extending onto membrane of wing; often with spots at opposite ends of chord somewhat larger. Forewing spots typically present at base of major forks, or at intersection of veins; those at base of discal and thyridial cells, and at base of fork V largest, usually evident even in weakly marked species; small spots often present at apex of R_1_, R_2_, R_3_, R_4+5_, M_1+2_, M_3+4_, Cu_1a_, and Cu_1b_, spots sometimes extending onto wing membrane, absent or indistinct in weakly marked species.

*Male genitalia*. Segment IX very short. Tergum X forming relatively short, narrow, declinate, mesal lobe with small apical sensilla (absent only in *Oecetis elata* Denning & Sykora), tergum often somewhat variable in length and shape, even within species. A pair of small papillate projections are present in the majority of species, lateral to the mesal lobe of tergum X. These are variable even within species and not considered to be of diagnostic value; no further mention is made of them in subsequent descriptions. Preanal appendage simple in structure, short to moderate in length (length ranging from about 1½ to 5 times maximum width), typically with basal setae short and fine, apical setae relatively coarse and elongate. Inferior appendage of characteristic form, somewhat mitten-like, with relatively broad, rounded dorsal lobe and notched or angularly demarcated ventral lobe, ventral lobe either projecting or not; marginal and mesal setae of dorsal lobe thickened, almost spine-like; lateral setae of ventral lobe typically short and fine; ventral lobe usually with slightly raised or rounded basomesal projection with short, thickened setae. Phallobase short or very short, widened apically, ventral margin more extended, often arched or bent, ventral apex (viewed caudally) either rounded (narrowly or widely U-shaped) or angular (V-shaped). Endotheca short, with distinct, short, ring-like phallotremal sclerite; asymmetrical lateral sclerite present in some species.

## Species descriptions

### Oecetis acciptrina sp. n.

http://zoobank.org/35CDDAA0-2FA5-4E14-9E23-0D6854C081A3

http://species-id.net/wiki/Oecetis_acciptrina

Figs 1 , 52 , Map 3

#### Diagnosis.

This species is readily identified by its small size and by the distinctive hooked apex of the phallobase, which is V-shaped in caudal view. In no other species that is similar in size, and within the geographic range of this species, is the ventral lobe of the inferior appendage so indistinctly demarcated from the dorsal lobe. Otherwise, this species is very similar in size and appearance to *Oecetis constricta* sp. n., or *Oecetis mexicana* sp. n., although it is slightly darker in color and with somewhat smaller forewing spots. Species sympatric with this species and also having the apex of the phallobase V-shaped in caudal view (*Oecetis metlacensis*, *Oecetis uncata* sp. n., and *Oecetis angularis* sp. n.) are much larger in size. The habitus figure for this species (Fig. 52) is of a female, included because the pinned male (holotype) was partially denuded of setae. The female is smaller than the male and slightly darker, but similarly marked.

#### Adult.

Forewing length: male (5.5 mm), female (5.3). Color yellowish-brown (darker than *Oecetis mexicana* sp. n.). Antennae whitish, with indistinct, narrow annulations at intersection of antennomeres. Forewing spots present, but relatively small and indistinct, not greatly contrasting with overall color of wing; spots at base of discal and thyridial cells, and base of fork V largest; veins of forewing chord lightly pigmented, relatively narrowly and evenly spaced, spots at opposite ends of chord evident; apical spots, at apices of major veins, present, but indistinct. Setae along veins in apical part of forewing semi-prostrate, somewhat laterally diverging, not conspicuously denser than on wing membrane. Fringe of setae along costal margin of forewing moderately dense, elongate, semi-erect.

#### Male genitalia.

**Figure 1.**
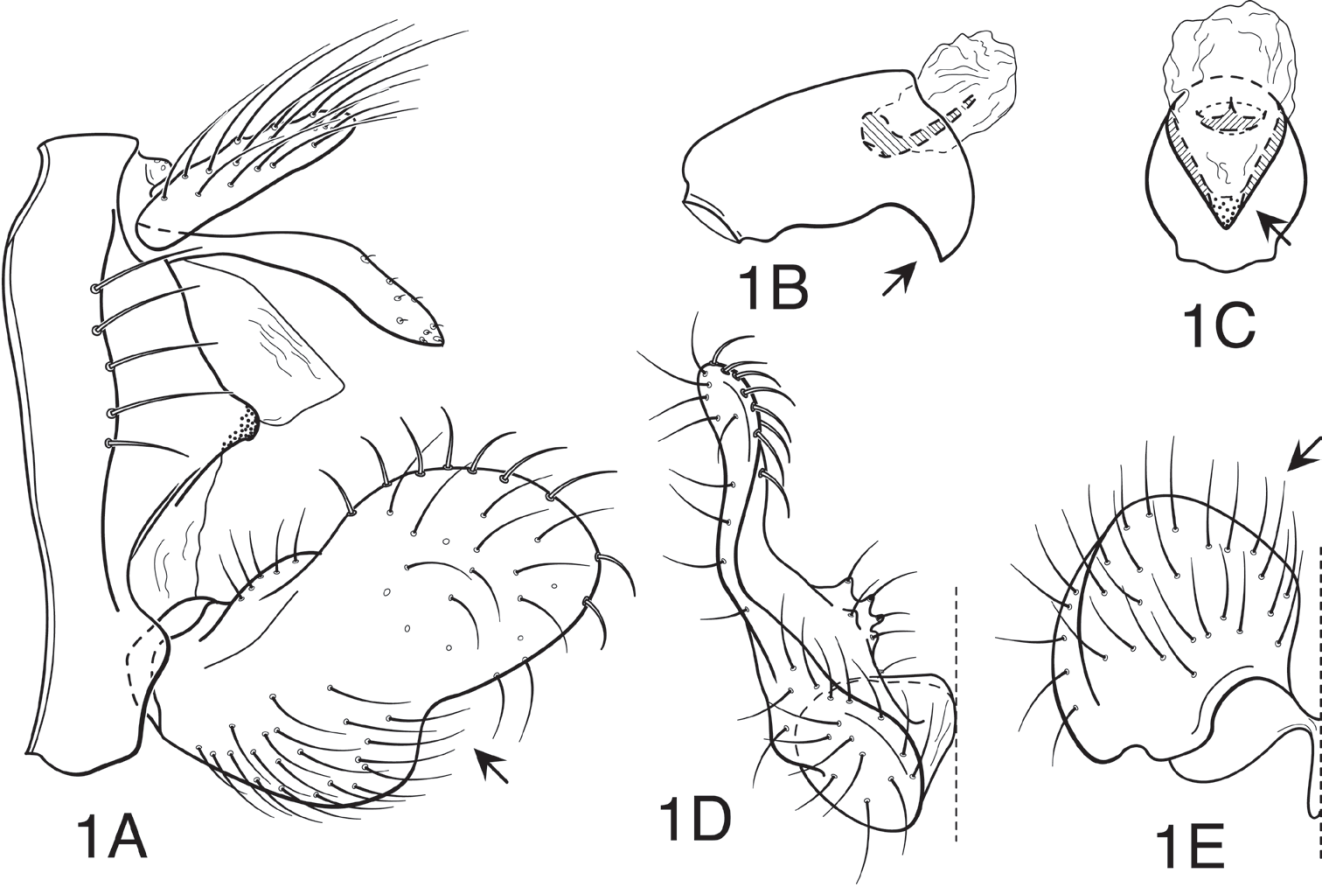
*Oecetis acciptrina* sp. n., male genitalia: **A** lateral **B** phallic apparatus, lateral **C** phallic apparatus, caudal **D** inferior appendage, caudal **E** inferior appendage, ventral.

Segment IX very short, with elongate setae along posterolateral margin. Tergum X with narrow, deflexed mesal lobe, apex of lobe inflated (lobulate), with small sensilla; lobe continuous basoventrally with short, paired lateral membranous projections. Preanal appendage moderately elongate, length about 3-4 times maximum width, simple in structure, apical setae elongate. Inferior appendage with prominent, but short, broadly rounded dorsal lobe and weakly projecting ventral lobe; ventral lobe either demarcated from dorsal lobe by broadly rounded invagination or forming weakly protruding, obtusely angled projection; posterior margin of ventral lobe, as viewed ventrally (Fig. 1E), with mesal bend rounded, not angular, apical projections of paired appendages not strongly diverging; basomesal projection of appendage very weakly projecting, with short, stiff setae; dorsal lobe with stout, mesally-curved setae on dorsal margin and stout, ventrally-curved setae on mesal surface. Phallobase short, tubular basally, ventral apex abruptly and strongly down-curved at about apical third, nearly at right angle with base, apex acute; ventral apex, as viewed caudally, V-shaped (distinctly keeled) (Fig. 1C). Phallotremal sclerite prominent, basally forming short tubular collar, ventral margin projecting, apex acute; asymmetrical lateral sclerite absent.

#### Holotype.

**Male** (pinned), **ECUADOR: Pichincha:** Santo Domingo (47 km S), Río Palenque Biol. Station, el 750 m, 29.vii.1976, J. Cohen (NMNH) (UMSP000208335).

#### Paratypes.

**COSTA RICA: Puntarenas:** Reserva Biologica Carara, Quebrada Bonita, 9°46.500'N, 84°36.300'W, el 35 m, 18–20.v.1990, Holzenthal & Blahnik, 1 male (alcohol) (UMSP); **PANAMA: Canal Zone:** Barro Colorado Island, 12.iii.1967, M. E. Irwin, 5 males, 6 females (alcohol) (NMNH); **Chiriqui:** Guadalupe Arriba, 8°52.433'N, 82°33.217'W, 6–12.vi.1984, H. Wolda, 1 male (alcohol) (NMNH); **ECUADOR: Pichincha:** same data as holotype, 2 females (pinned) (NMNH).

#### Etymology.

This species is named *Oecetis acciptrina* after the Latin word *accipter*, a hawk, and referring to the phallobase in this species, which has its ventral apex strongly bent, somewhat resembling a hawk’s bill.

### Oecetis agosta sp. n.

http://zoobank.org/C87FB5D2-278A-4C7A-AEA5-75F91DFEC287

http://species-id.net/wiki/Oecetis_agosta

Figs 2 , 54 , Map 3

#### Diagnosis.

This species is most readily identified by the peculiar shape of the phallobase of the male, which is distinctly bent or elbowed in the middle and has a protruding apex and a ventral margin that is noticeably convexly bulged before the apex. In overall morphology the genitalia are nearly intermediate between *Oecetis verrucula* sp. n. and *Oecetis protrusa* sp. n., agreeing with the former in having an inferior appendage with a very acutely angled and distinctly protruding ventral lobe, and with the latter in the general form of the phallobase, with a protruding apex and preapical convex bulge on the ventral margin. However, the dorsal lobe of the inferior appendage is broader than in *Oecetis verrucula* and the phallobase is much more angularly bent or elbowed in the middle than *Oecetis protrusa*. Additionally, unlike *Oecetis protrusa*, which has very large, conspicuous forewing spots, and *Oecetis verrucula*, which has smaller, but distinct spots, *Oecetis agosta* is nearly immaculate, with only the faintest trace of pigmentation at the branch points of the major veins. However, the available material for *Oecetis agosta* is very limited and it is impossible to assess how constant this latter character may be. Although this species is currently known from a single locality in Mexico, and from very limited material, it is distinctive enough to suggest that it should be recognized as a new species.

#### Adult.

Forewing length: male (7.6 mm). Color yellowish. Antennae whitish with indistinct, narrow annulations at intersection of antennomeres. Forewing spots not apparent (slight trace of pigmentation at base of discal and thyridial cells and base of fork V); veins of forewing chord narrowly spaced, *s* and *r-m* veins very close; chord very lightly, not evidently, pigmented; apical spots of forewing absent. Setae along veins in apical part of forewing mostly apically directed, a few moderately, not conspicuously, laterally diverging. Fringe of setae along costal margin of forewing dense, short, not strongly projecting.

#### Male genitalia.

**Figure 2.**
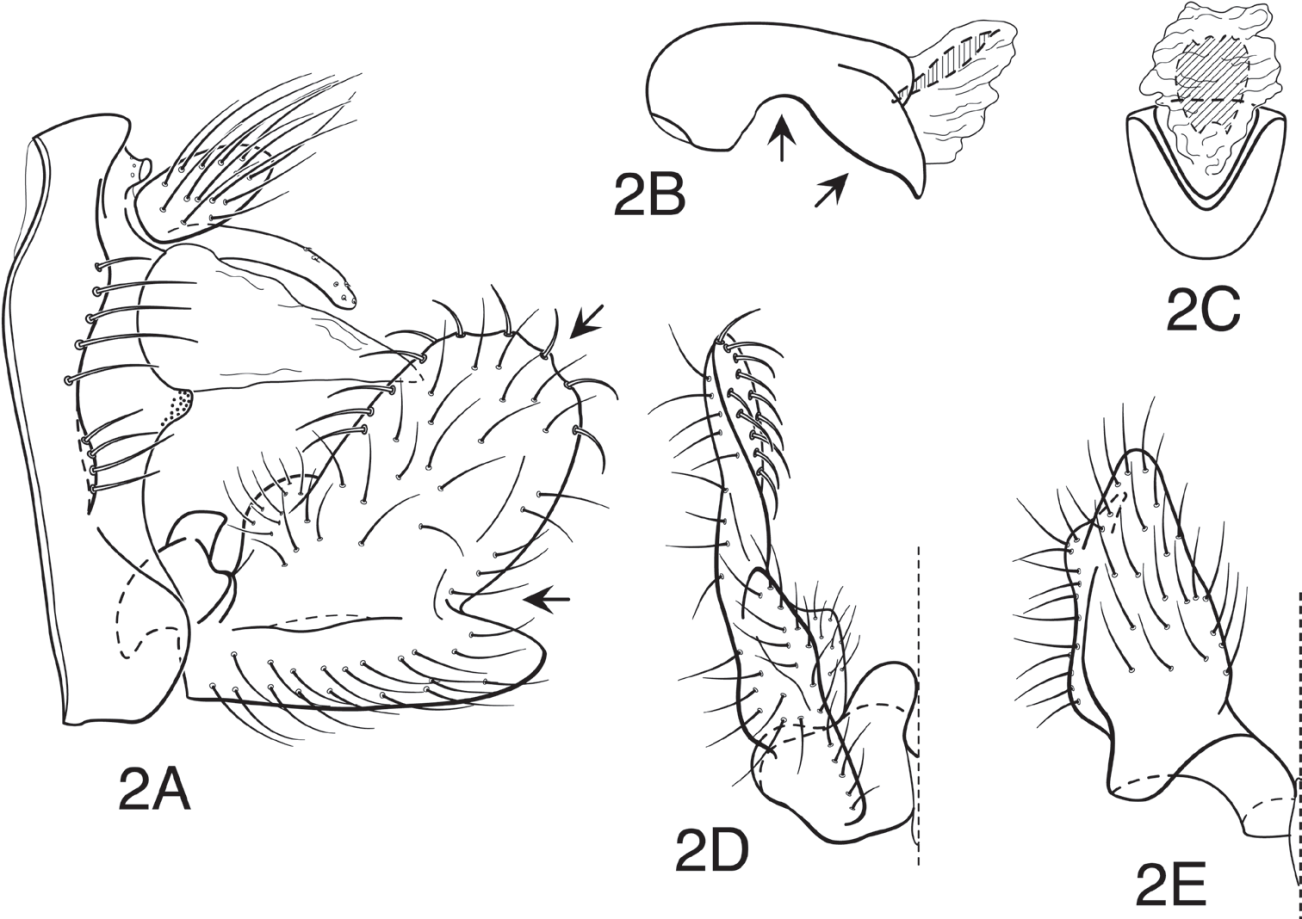
*Oecetis agosta* sp. n., male genitalia: **A** lateral **B** phallic apparatus, lateral **C** phallic apparatus, caudal **D** inferior appendage, caudal **E** inferior appendage, ventral.

Segment IX very short, with elongate setae along posterolateral margin. Tergum X with narrow, deflexed mesal lobe, lobe nearly uniform in width, narrowing apically, with small sensilla; lobe continuous basoventrally with projecting lateral membranous projections. Preanal appendage somewhat laterally compressed, short, length about 2 times maximum width, simple in structure, apical setae elongate. Inferior appendage with prominent, very broadly rounded dorsal lobe and angularly projecting ventral lobe; projection of ventral lobe prominent and strongly protruding, forming acute angle with dorsal lobe, angle abrupt; posterior margin of ventral lobe, as viewed ventrally (Fig. 2E), very weakly bent near base, apices of paired lobes only slightly diverging; basomesal projection of appendage prominent, rounded, with short, stiff setae; dorsal lobe with stout, mesally-curved setae on dorsal margin and stout, ventrally-curved setae on mesal surface. Phallobase moderately elongate, strongly elbowed in middle, ventral apex deflexed and very distinctly projecting, ventral margin slightly convexly bulging before apex; ventral apex, as viewed caudally, narrowly U-shaped (not keeled preapically). Phallotremal sclerite prominent, basally forming very narrow, tubular collar, ventral margin projecting and broadly rounded apically; asymmetrical lateral sclerite absent.

#### Holotype.

**Male** (pinned), **MEXICO: San Luis Potosí:** El Salto Falls, 23–24.vi.1965, O.S. Flint (NMNH) (UMSP000208285).

#### Paratypes.

**MEXICO:** same data as holotype, 1 male (alcohol) (NMNH).

#### Etymology.

This species is named *Oecetis agosta*, after the Greek word *agostos*, a bent arm, and referring to the distinctly elbowed or bent phallobase of this species.

### Oecetis angularis sp. n.

http://zoobank.org/CBD87120-82F9-4DF7-9062-40993116FD69

http://species-id.net/wiki/Oecetis_angularis

**Figure 3.**
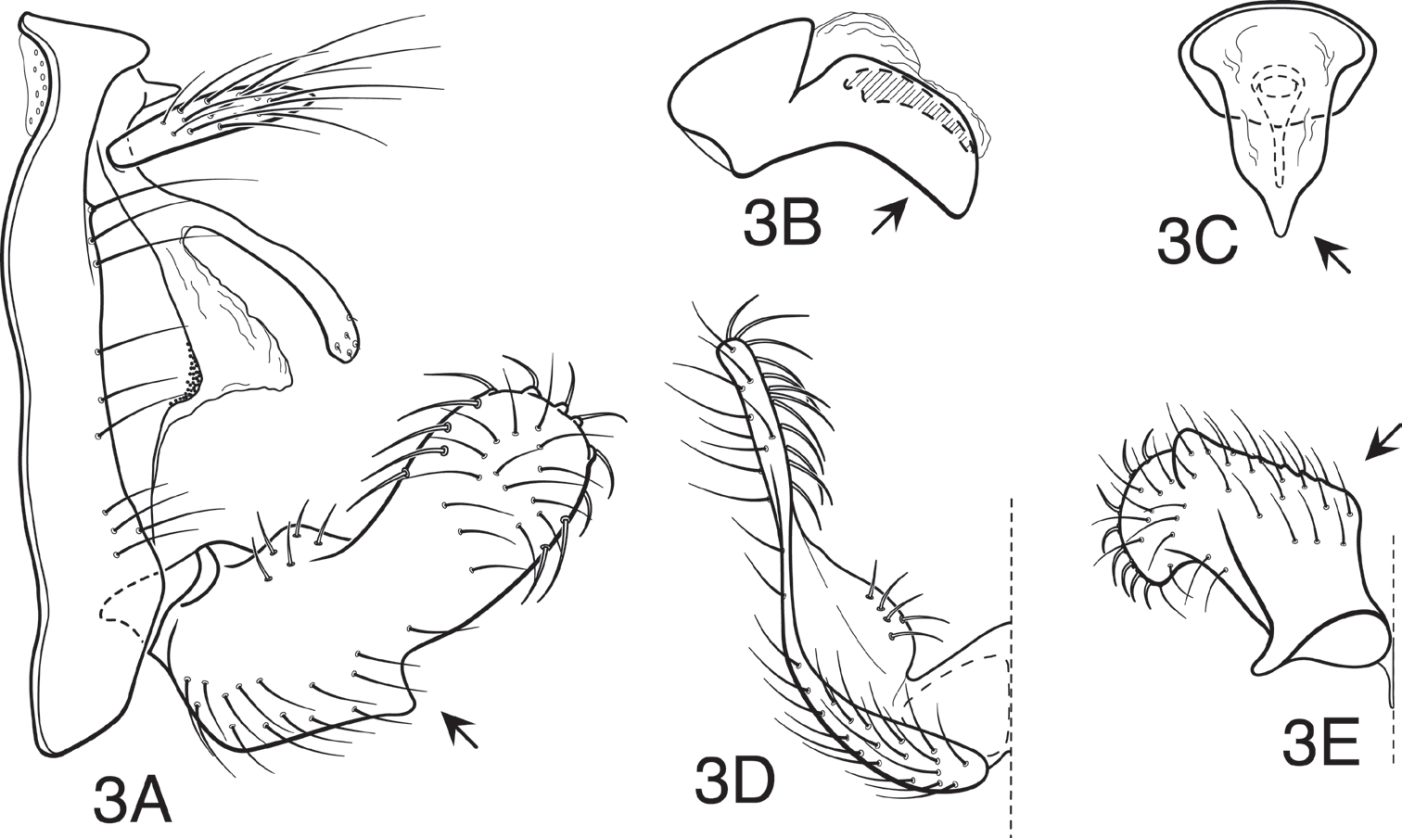
*Oecetis angularis* sp. n., male genitalia: **A** lateral **B** phallic apparatus, lateral **C** phallic apparatus, caudal **D** inferior appendage, caudal **E** inferior appendage, ventral.

Figs 3 , 33 , 47 , Map 1

#### Diagnosis.

*Oecetis angularis* sp. n. is diagnosed by its intermediate size, coloration (yellow, with distinct forewing spots), weakly protruding, but angularly demarcated ventral lobe of the inferior appendage (Fig. 3A), and by having a phallobase with its ventral apex acutely angled (V-shaped), as viewed caudally (Fig. 3C). The ventral margin of the phallobase is arched rather than strongly bent, as viewed laterally. Other species in which the apex of the phallobase is V-shaped in caudal view include *Oecetis metlacensis*, *Oecetis uncata* sp. n., and *Oecetis apache* sp. n., all of which are slightly larger in size, darker in color (yellowish-brown or light brown), and have the phallobase more strongly arched or bent as viewed laterally, and *Oecetis acciptrina*, which is much smaller in size and has the phallobase very strongly bent or hooked near its apex as viewed laterally. *Oecetis metlacensis* and *Oecetis apache* additionally differ by having much less prominent forewing spots (absent in *Oecetis apache*).

Despite the resemblance of the genitalia to the above mentioned species, in overall appearance (size, color and forewing spotting) *Oecetis angularis* probably more closely resembles *Oecetis protrusa* sp. n., *Oecetis tumida* sp. n., or *Oecetis mexicana*, sp. n., all of which differ in having the apex of the phallobase U-shaped in caudal view (although the shape of the apex may be somewhat difficult to discern in *Oecetis tumida* due to its bulbous expansion). A distinction can be made on the basis that the ventral margin of the phallobase is not “keeled” preapically in species described as having a U-shaped apex. The general shape of the inferior appendage in *Oecetis angularis*, with a weakly protruding ventral lobe, is most similar to *Oecetis tumida*, which is also similar in size; *Oecetis tumida* differs in having the ventral margin the phallobase noticeably convexly rounded preapically, as viewed laterally (Fig. 24B), somewhat as in *Oecetis protrusa*. In Costa Rica, where *Oecetis angularis* and *Oecetis protrusa* have been found together, *Oecetis angularis* is generally somewhat larger in size than *Oecetis protrusa*. In other parts of their ranges, however, size differences seem to be less apparent.

In size, color, and forewing spotting *Oecetis angularis* is also nearly identical to *Oecetis punctata*, with which it may co-occur. However, the differences in the shape of the inferior appendages of the males, as well as the arrangement of the crossveins of the chord, as discussed above for characters separating the *Oecetis avara* and *Oecetis punctata* groups, should readily distinguish them.

#### Adult.

Forewing length: male (9.3–10.8 mm); female (6.3–9.0 mm). Color pale yellowish to brownish-yellow (similar to *Oecetis mexicana*); forewing spots distinct, spots at base of discal and thyridial cells large or moderately large, other spots smaller; veins of forewing chord widely spaced, *s* and *r-m* veins slightly closer; chord with small spots at juncture of major veins, spots at opposite ends of chord larger; spots at apices of major veins small, pigmentation confined to vein. Setae along veins in apical part of forewing elongate, semi-prostrate, laterally diverging. Fringe of setae along costal margin of forewing moderate in length, somewhat projecting, not conspicuously erect.

#### Male genitalia.

Segment IX very short, with elongate setae along posterolateral margin. Preanal appendage moderately elongate, simple in structure, apical setae more elongate. Tergum X with elongate, narrow, deflexed mesal lobe, apex of lobe slightly enlarged, with numerous small sensilla; mesal lobe continuous basoventrally with short, paired lateral membranous or lightly sclerotized projections. Inferior appendage with prominent rounded dorsal process and angularly projecting ventral process; ventral process not strongly projecting, forming an approximate right angle with dorsal process; basomesal projection rounded, moderately prominent, with short, stout mesally directed setae; dorsal process with stout, ventrally curved setae; base of appendage, as viewed ventrally (Fig. 3E), distinctly angled. Phallobase short and tubular basally, with relatively elongate, arched ventral apex; apex, as viewed caudally, with ventral margin sharply V-shaped and slightly keeled (Fig. 3C); phallotremal sclerite elongate, prominent.

#### Holotype.

**Male**(pinned), **COSTA RICA: Puntarenas:** Río Bellavista, ca. 1.5 km NW Las Alturas, 8°57.060'N, 82°50.760'W, el 1400 m, 16-17.iii.1991, Holzenthal, Muñoz, Huisman (UMSP) (UMSP000208089).

#### Paratypes.

**GUATEMALA: Suchitepequez:** Finca Moca, 12.vi.1966, Flint & Ortiz, 1 male, 1 female (pinned) (NMNH); Puente Ixtacapa, 18-19.vi.1966, Flint & Ortiz, 1 male (pinned)(NMNH); **COSTA RICA:** Arenal, Quebrada Trondorancito, 24.vii.1967, O. S. Flint, Jr., 13 males, 9 females (pinned) (NMNH); La Suiza, 17.vi.1967, Flint & Ortiz, 1 male (pinned) (NMNH); Río Seco, NW Esparta, 28.vii.1967, O. S. Flint, Jr., 1 male (alcohol) (NMNH); **Alajuela:** Reserva Forestal San Ramon, Río San Lorencito and tribs., 10°12.960'N, 84°36.420'W, el 980 m, 30.iii.-1.iv.1987, Holzenthal, Hamilton, Heyn, 1 male (alcohol) (UMSP); Río Pizote, ca. 5 km N Dos Ríos, 10°56.880'N, 85°17.460'W, el 470 m, 9.iii.1986, Holzenthal & Fasth, 1 male (pinned), 16 males, 15 females (alcohol) (UMSP); **Cartago:** Río Reventazón in CATIE along Sendero Espaveles, 9°53.580'N, 83°39.060'W, el 500 m, 22.iii.1991, Muñoz, 5 males, 2 females (pinned), 1 male (alcohol) (UMSP); **Limón:** Río Banano, 16 km WSW Bomba, 9°52.280'N, 83°10.020'W, el 150 m, 26.iii.1987, Holzenthal, Hamilton, Heyn, 2 males, 2 females (pinned) (UMSP); Río Telire and small trib., SE Suretka, 9°33.240'N, 82°53.520'W, el 48 m, 1.ii.1986, 1 male, 1 female (pinned) (UMSP); Res. Biol. Barbilla, Río Dantas, 9°59.640'N, 83°26.580'W, el 300 m, 27-30.i.1992, Holzenthal, Muñoz, Kjer, 7 males (alcohol) (UMSP); **Puntarenas:** same data as holotype, 15 males, 8 females (pinned) (UMSP); Río Cotón in Las Alturas, 8°56.280'N, 82°49.560'W, el 1360 m, 18.iii.1991, Holzenthal Muñoz, Huisman, 18 males, 7 females (pinned), 10 males, 18 females (alcohol) (UMSP); same location, 12.viii.1990, Holzenthal, Blahnik, Muñoz, 7 males, 1 female (pinned), 8 males (alcohol) (UMSP); Río Jaba, 2.4 km (air) NW San Vito, 8°49.920'N, 82°59.460'W, el 970 m, 13.vi.1986, Holzenthal, Heyn, Armitage, 1 male (pinned), 1 male, 1 female (alcohol) (UMSP); Río Ceibo, route 2, ca. 6 km W road to Buenos Aires, 9°8.940'N, 83°22.620'W, el 250 m, 20.ii.1986, Holzenthal, Morse, Fasth, 1 male (alcohol) (UMSP); Río Singri, ca. 2 km (air) S Finca Helechales, 9°3.420'N, 83°4.920'W, el 720 m, 21.ii.1986, Holzenthal, Morse, Fasth, 10 males, 20 females (alcohol) (UMSP); **ECUADOR: Cotopaxi:** Latacunga, 133 km W, 2.vii.1975, el 1080 m, A. Langley & J. Cohen, 1 male (pinned) (NMNH); Quevedo, 36 km NE, el 335 m, 2.vii.1976, J. Cohen, 9 males, 4 females (alcohol) (NMNH); **Loja:** Río Puyango, el 300 m, 17-18.viii.1977, L. E. Peña G., 2 males, 1 female (pinned) (NMNH); **Pichincha:** Santo Domingo de los Colorados, 14. km E, 5.vii.1975, Langley & Cohen, 1 male (pinned) (NMNH).

#### Etymology.

This species is named *Oecetis angularis* for the acutely angled ventral apex of the phallobase, as seen in caudal view.

### Oecetis apache sp. n.

http://zoobank.org/2C581B2E-8AFA-41C2-920F-8A3AC519121C

http://species-id.net/wiki/Oecetis_apache

Figs 4 , 37 , Map 2

Oecetis metlacensis . Houghton 2001: 91.

#### Diagnosis.

In size, general color and diagnostic features, which include a phallobase angularly bent or strongly arched in the middle and with a V-shaped apex as viewed caudally, this species most closely resembles *Oecetis metlacensis*, and was identified as such by Houghton (2001) in reporting that species for the first time from the United States. However, on direct comparison (and as noted by Houghton) there are some differences. Notably, the dorsal lobe of the inferior appendage is more robust, the angle it makes with the ventral lobe is less pronounced, and the forewings seem to lack any spotting at all. Additionally, the forewings are proportionately wider than those of *Oecetis metlacensis*. In color and general form, particularly in the overall shape of the inferior appendages, it is also very similar to *Oecetis disjuncta*, which differs in that the apex of the phallobase is U-shaped or rounded in caudal view, rather than V-shaped.

#### Adult.

Forewing length: male (11.5–12.8 mm), female (10.3 mm). Color tan (light brown). Antennae whitish with indistinct, narrow annulations at intersection of antennomeres. Forewing spots absent; veins of forewing chord with *m* widely spaced, *s* and *r-m* veins relatively close. Forewings slightly broader than *Oecetis metlacensis*. Setae along veins in apical part of forewing mostly apically directed, not or only slightly laterally diverging. Fringe of setae along costal margin of forewing dense, short, not strongly projecting.

#### Male genitalia.

**Figure 4.**
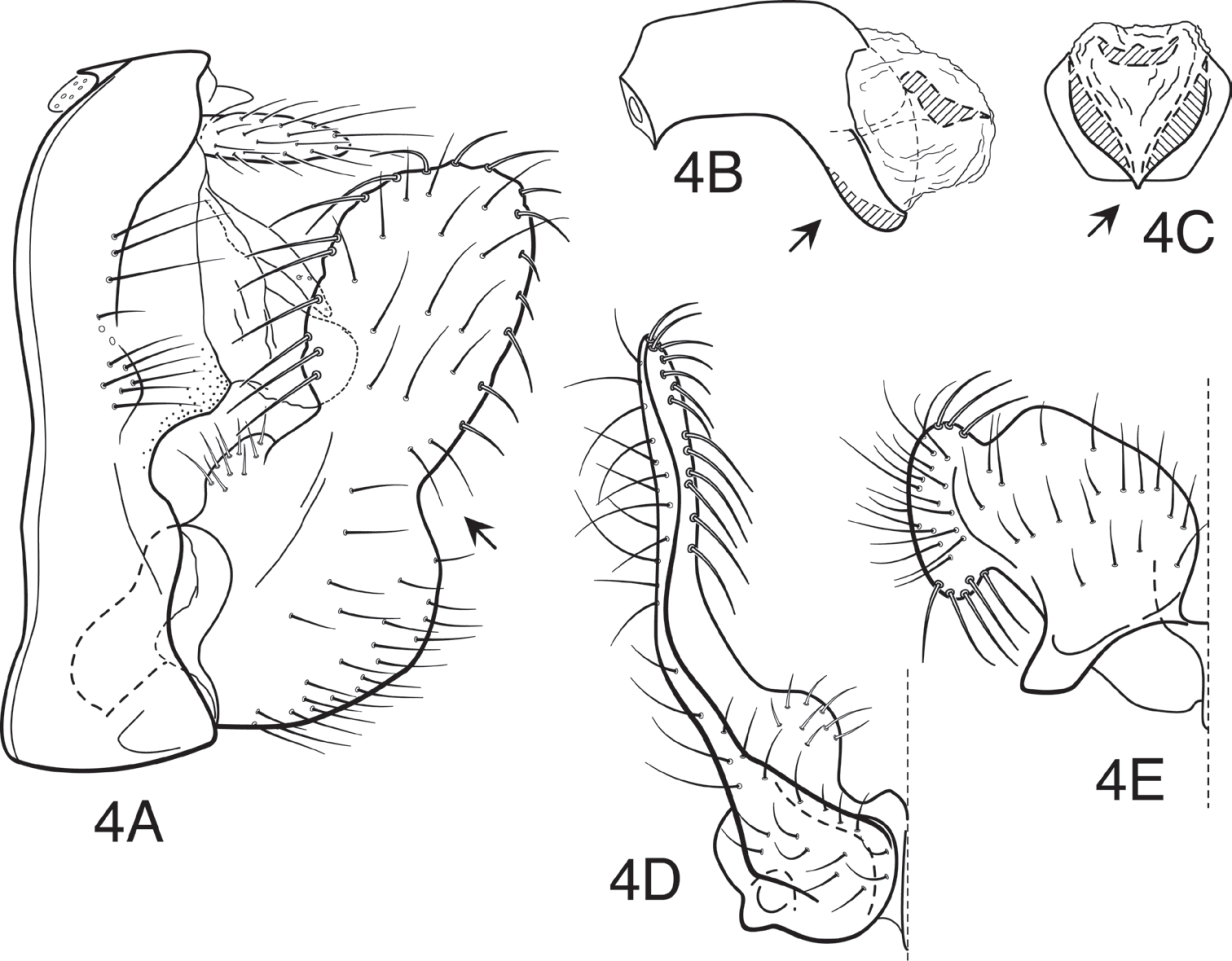
*Oecetis apache* sp. n., male genitalia: **A** lateral **B** phallic apparatus, lateral **C** phallic apparatus, caudal **D** inferior appendage, caudal **E** inferior appendage, ventral.

Segment IX very short, with elongate setae along posterolateral margin. Tergum X with narrow, deflexed mesal lobe, lobe short, tapering apically, apex with small sensilla; lobe continuous basoventrally with paired lateral membranous projections. Preanal appendage moderately elongate, length 3-5 times maximum width (in some as long as *Oecetis disjuncta*), simple in structure, apical setae elongate. Inferior appendage with prominent rounded dorsal lobe and scarcely projecting ventral lobe, separated from dorsal lobe by shallow, rounded notch; posterior margin of ventral lobe, as viewed ventrally (Fig. 4E), with rounded bend near base, apices of lobes strongly diverging; basomesal projection of appendage forming distinct rounded projection with short, stiff setae; dorsal lobe with stout, mesally-curved setae on dorsal margin, anteroventrally directed setae on anterior margin, and ventrally-curved setae on mesal surface. Phallobase relatively elongate and tubular basally, ventral apex about as long as base, strongly, subangularly deflexed; ventral apex, as viewed caudally, V-shaped (distinctly keeled) (Fig. 4C). Phallotremal sclerite prominent, basally forming tubular collar, ventral margin somewhat projecting, apex acute; asymmetrical lateral sclerite absent.

#### Holotype.

**Male** (pinned), **USA: Arizona:** Apache Co., W. Fork Black R., FR 68 @ West Fork Campground, 33°46.676'N, 109°24.291'W, el 2438 m, 18.vi.1999, D. Houghton (UMSP) (UMSP000021236).

#### Paratypes.

**USA: Arizona:** same locality and date as holotype, 5 males, 1 female (pinned) (UMSP) (NMNH); same locality, 19.vi.1999, D.C. Houghton, 24 males (alcohol) (UMSP); Apache Co. E Fork Black River, F.R. 249, Three Forks Crossing, Apache-Sitgeaves National Forest, 33°51.300'N, 109°18.890'W, el 2530 m, 18.vi.1999, D.C. Houghton, 5 males (alcohol) (UMSP); **New Mexico:** Catron Co., Gila National Monument, el 1829 m, 4.vii.1964, D.R. Davis, 1 male (pinned) (NMNH).

#### Etymology.

This species is named *Oecetis apache*, used as a noun in apposition, after Apache Co., Arizona, where the holotype specimen was collected, but also referring to its general southwestern U.S. distribution, homeland of the Apache.

### Oecetis avara

(Banks)

http://species-id.net/wiki/Oecetis_avara

**Figure 5.**
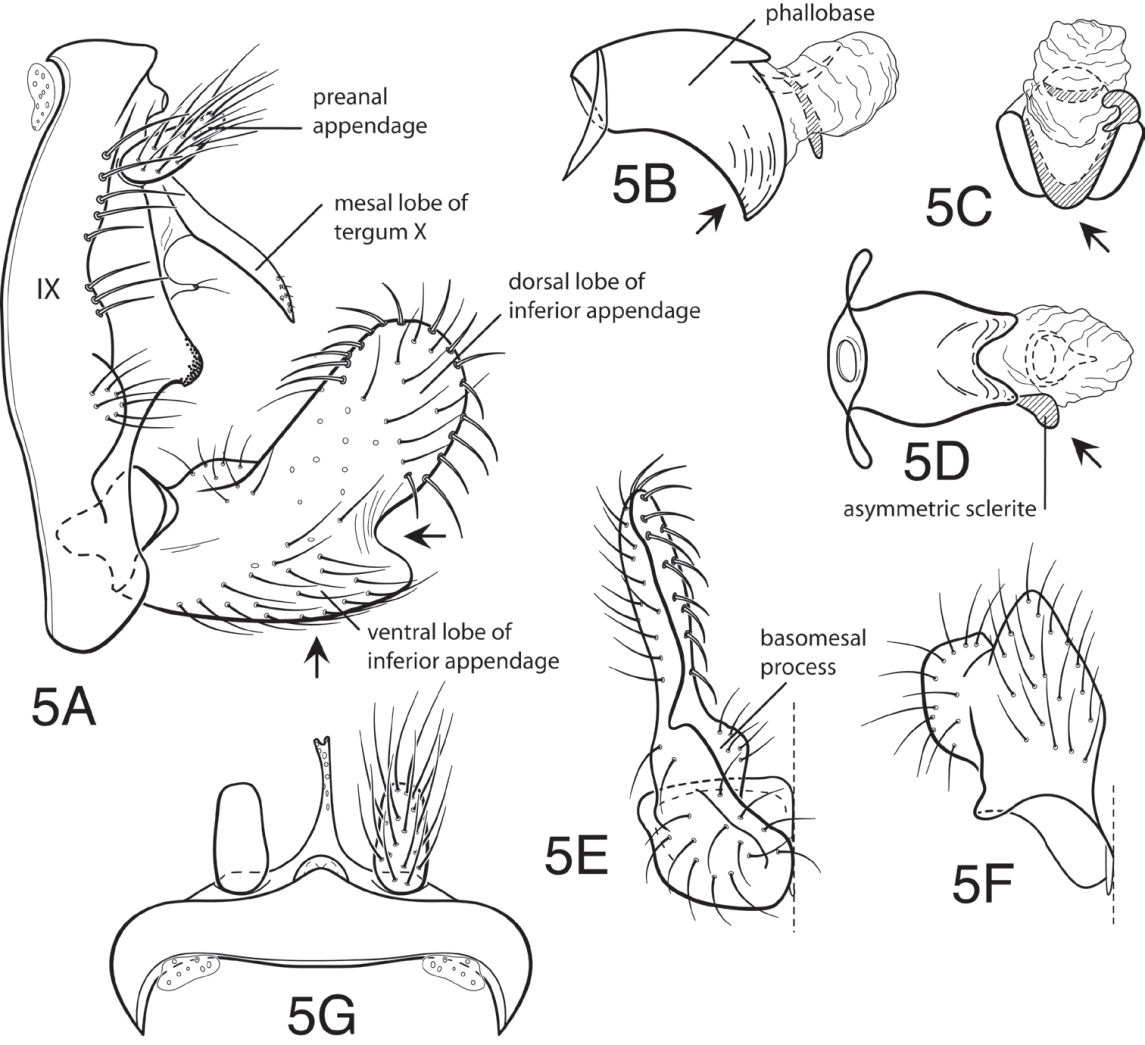
*Oecetis avara* (Banks), Holotype, male genitalia: **A** lateral **B** phallic apparatus, lateral **C** phallic apparatus, caudal **D** phallic apparatus, ventral **E** inferior appendage, caudal **F** inferior appendage, ventral **G** segment IX and tergum X, dorsal.

Figs 5 , 13 , 14 , 40 , 41 , Map 2

**Figure 6.**
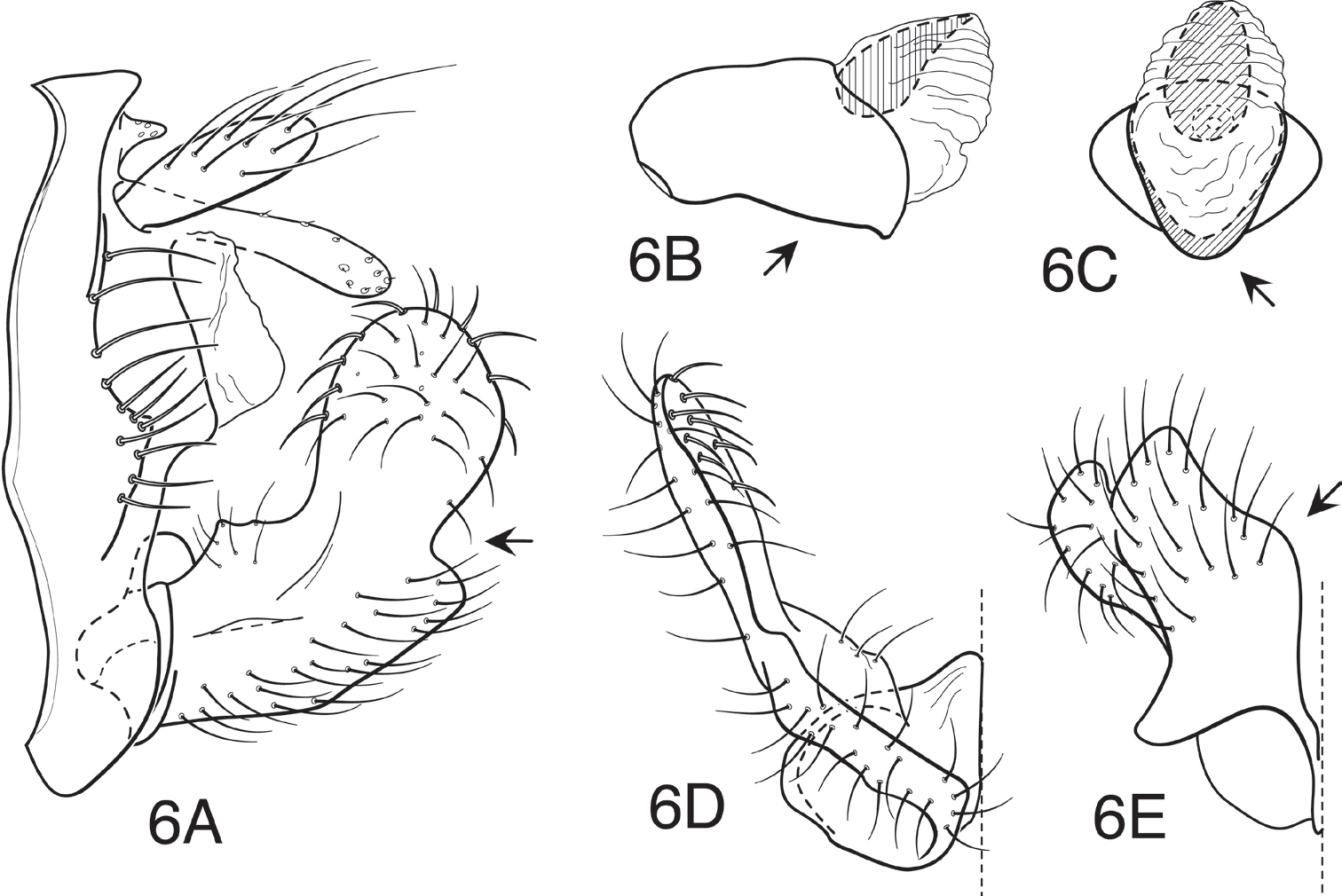
*Oecetis campana* sp. n., male genitalia: **A** lateral **B** phallic apparatus, lateral **C** phallic apparatus, caudal **D** inferior appendage, caudal **E** inferior appendage, ventral.

Setodes avara Banks 1895: 316. [holotype male, Sherbrook Canada, pinned with genitalia in vial, MCZ type 11553].
Oecetina avara (Banks): Banks 1899: 214.
Oecetodes avara (Banks): Ulmer 1905: 129.
Oecetis avara (Banks): Sibley 1926: 105, 189; Betten 1934: 269 (plate 34, Figs 1–3) [description, illustration]; Ross 1944: 240 (figures 813, 818A, 828) [description, illustration]; Denning 1956: 267 (fig. 10: 34d) [illustration]; Smith and Lehmkuhl 1980: 641 (Figs 3–4, 6, 8, 11, 15, 17, 19–20, 22, 24, 27–28, 35–36, 39, 42, 44, 49, 50) [description, illustration, distribution]; Bueno and Flint 1980: [distribution – Mexico, Central America, Venezuela]; Bueno-Soria 1981: 111 (fig. 5A) [illustration only]; Flint 1981: 96 (fig. 364) [illustration, distribution – Colombia]; Botosaneanu and Alkins-Koo 1993: 35 [distribution – Trinidad & Tobago]; Flint 1996a: 104 [distribution – Trinidad & Tobago]; Flint 1996b: 423 [distribution – Peru]. Moulton and Stewart 1996: 150 (fig. 407) [illustration, distribution]; Rueda Martín et al. 2011: 21 (fig. 2A-F) [illustration, distribution – Bolivia].
Oecetis (Oecetodes) avara (Banks): Fischer 1966: 153; 1972: 150.
Oecetis (Pseudosetodes) avara (Banks): Chen 1993 (unpublished): (Figs 27A–D, J-M) [description, illustration, distribution].

#### Diagnosis.

This species is similar to and most likely to be confused with *Oecetis houghtoni* sp. n., especially due to their sympatric distributions and relatively minor morphological differences. Unfortunately, the range of variation for the 2 species has not been fully established and specimens from some localities may not be currently diagnosable if they do not conform closely to the holotypes for the 2 species (see remarks section following the species description). The holotype of *Oecetis avara* is characterized by a very well developed ventral lobe of the inferior appendage that forms a prominent, acutely angled projection. The ventral margin is distinctly elongate (see Figs 11–14 for comparison to *Oecetis houghtoni*). Some evidence of “wrinkling” is apparent at the point of articulation between the dorsal and ventral lobes; this is distinctly more noticeable than in *Oecetis houghtoni* due to the sharper angle of articulation. Also, as compared to *Oecetis houghtoni*, the phallobase is usually more distinctly sclerotized and arched ventrally, with its apex strongly downturned and very finely striate, often with the extreme apex slightly ridged or burred. Unfortunately, characters of the phallobase are subtle and also variable within the 2 species and thus may not always be completely diagnostic. Although the holotype of *Oecetis avara* itself is grayish-brown in color, with forewings only weakly spotted (possibly as a result of the specimen being somewhat faded), specimens from most areas of its distribution, including those from Minnesota, are distinctly lighter in color than *Oecetis houghtoni* and also have more prominent forewing spots. In Minnesota, *Oecetis avara* can be distinguished from *Oecetis houghtoni* by color alone (Figs 40 and 41).

Despite the close similarity and difficulty of distinguishing *Oecetis avara* from *Oecetis houghtoni*, another species that closely resembles *Oecetis avara*, both in color and genitalic aspects, is *Oecetis verrucula* sp. n. from Central America. The only significant difference is the possession of a small rugose wart or projection preapically on the phallobase in *Oecetis verrucula*, similar to the character state in *Oecetis sordida*, sp. n. This character is absent in *Oecetis avara*, but suggested by the fine striations often observed near the apex of the phallobase. COI barcode data (Fig. 58) also suggest that *Oecetis avara* and *Oecetis verrucula* are more closely related to each other than to *Oecetis houghtoni*. Rationale for treating *Oecetis verrucula* as a separate species is discussed in its diagnosis and description.

#### Adult.

Forewing length: male (8.2–10.3 mm), female (7.5–8.7 mm). Color generally pale yellowish-brown (slightly darker than *Oecetis mexicana* sp. n., paler than *Oecetis houghtoni* sp. n.). Antennae whitish with indistinct, narrow annulations at intersection of antennomeres. Forewing spots small, but distinct; spots at base of discal and thyridial cells and base of fork V largest, other spots indistinct; veins of forewing chord relatively widely spaced (wider than *Oecetis houghtoni* sp. n.), either evenly spaced or *s* and *r-m* veins slightly closer; chord with crossveins pigmented or with small spots at juncture of major veins; apical spots, at apices of major veins, indistinct, but usually evident, pigmentation extending slightly beyond veins. Setae along veins in apical part of forewing only moderately elongate, semi-prostrate, laterally diverging. Fringe of setae along costal margin of forewing dense, short, not strongly projecting.

#### Male genitalia.

Segment IX very short, with elongate setae along posterolateral margin. Tergum X with narrow, deflexed mesal lobe, apex of lobe tapered or rounded, with small sensilla; lobe continuous basoventrally with short, paired lateral membranous projections. Preanal appendage moderately elongate, length 2–3 times maximum width (longer, on average, than *Oecetis houghtoni* sp. n.), simple in structure, apical setae elongate. Inferior appendage with prominent rounded dorsal lobe and angularly projecting ventral lobe; projection of ventral lobe prominent and strongly protruding, typically forming acute angle with dorsal lobe, angle usually abrupt, causing appendage at basal angle to be somewhat “wrinkled;” posterior margin of dorsal lobe evenly rounded, not angulate; posterior margin of ventral lobe, as viewed ventrally (Fig. 5F), only weakly bent near base; basomesal projection of appendage rounded, with short, stiff setae; dorsal lobe with stout, mesally-curved setae on dorsal margin and stout, ventrally-curved setae on mesal surface. Phallobase very short, tubular basally, ventral apex strongly down curved, often noticeably sclerotized, with minute striations; ventral apex, as viewed caudally, U-shaped (Fig. 5C). Phallotremal sclerite prominent, basally forming short tubular collar, ventral margin projecting, apex acute; asymmetrical lateral sclerite present.

#### Material examined.

**CANADA: Quebec:** Sherbrook, male holotype (pinned) (MCZ); **USA: Alabama:** Bibb Co., Little Cahaba River, 4 mi NE Sixmile, 23.v.1988, C.M. & O.S. Flint, Jr., 1 male (pinned) (NMNH); **Florida:** Jackson Co., Chipola River at County Road 280, 1.5 mi E Oakdale, 11.vi.1995, V.L. Nations & M.J. Howe, 6 males, 4 females (pinned) (UMSP); **Kansas:** Clark Co., 12 mi W, 5 mi S Columbus, 8-9.vi.1976, G.F. Hevel, 2 males (pinned) (NMNH); **Maryland:** Montgomery Co., Potomac River, Carderock, 27.viii.1981, Flint & Butler, 1 male (pinned) (NMNH); **Minnesota:** Aitkin Co., Mississippi River, County Road 3, 47°01.417'N, 93°16.5000'W, 12.vii.2000, D.C. Houghton, 321 males, 497 females (alcohol) (UMSP); Aitkin Co., Hay Lake, Hay Lake State Park, 46°57.500'N, 93°13.000'W, 12.vii.2000, D.C. Houghton, 1 female (alcohol) (UMSP); Anoka Co., Coon Creek, confl. Mississippi River, 45°08.000'N, 93°17.000'W, 10.vii.2000, D.C. Houghton, 13 males, 8 females (alcohol) (UMSP); Blue Earth Co., Minnesota River, Mile 112, Sibley Park, Mankato, 11.vii.1979, N. Potthoff, 1 male, 1 female (alcohol) (UMSP); Blue Earth Co., Minneopa Creek, Minneopa State Park, 44°09.034'N, 94°05.500'W, 10.vi.2000, D.C. Houghton, 1 male, 1 female (alcohol) (UMSP); Blue Earth Co., un. sp. 1, Minneopa State Park, 44°09.667'N, 94°05.500'W, 10.vi.2000, D.C. Houghton, 1 male, 2 females (alcohol) (UMSP); Blue Earth Co., Le Seur River, County Road 16, public access, 44°04.916'N, 94°00.583'N, 11.vi.2000, D.C. Houghton, 17 males, 13 females (alcohol) (UMSP); Blue Earth Co., Maple River, County Road 166, public access, 44°00.034'N, 94°03.083'W, 11.vi.2000, D.C. Houghton, 198 males, 97 females (alcohol) (UMSP); Brown Co., un. sp., Cottonwood River, Flandrau State Park, 44°17.333'N, 94°28.916'W, 09.vi.2000, D.C. Houghton, 6 males, 17 females (alcohol) (UMSP); Brown Co., Cottonwood River, Flandrau State Park, 44°17.545'N, 93°28.134'W, 02.viii.1999, D.C. Houghton, 14 males, 1 female (pinned), 96 males, 53 females (alcohol) (UMSP); Chisago Co., Sunrise River at Kost Dam County Park, ca. 1.5 km S Kost, 45°28.75'N, 92°52.54'W, el 280 m, 13.vii.2004, Holzenthal et al., 3 males, 3 females (pinned) (UMSP); Crow Wing Co., Pine River, County Road 11, public access, 46°34.583'N, 94°01.834'W, 16.ix.2000, D.C. Houghton, 3 males, 2 females (alcohol) (UMSP); Crow Wing Co., Pine River, County Road 11, 46°34.333'N, 94°02.000'W, 29.vi.2000, D.C. Houghton, 214 males, 222 females (alcohol) (UMSP); Fillmore Co., Middle Br. Root River, Co. Rd. 21, ca. 5 mi NW Lanesboro, 43°49.080'N, 92°0.900'W, el 283 m, 13.vii.1991, R.J. Blahnik, 1 male (pinned) (UMSP); Fillmore Co., Spring Valley, 15.vi.1935, 1 male (pinned) (UMSP); Koochiching Co, Rainey R., confl. Little Fork River, State Highway 11 nr. International Falls, 48°31.174'N, 93°34.174'W, 12.vii.1999, D.C. Houghton, 983 males, 1461 females (alcohol) (UMSP); Koochiching Co., Big Fork River, State Highway 11, public access, 48°30.700'N, 93°42.754'W, 12.vii.1999, D.C. Houghton, 35 males, 5 females (pinned), 421 males, 203 females (alcohol) (UMSP); Koochiching Co., Little Fork River, Minn. F.S., 17.vii.1968, E.F. Cook, 1 male (alcohol) (UMSP); Koochiching Co., Tamarac River, Pine Island State Forest, Balsiger F.R., 7 km E Waskish, 48°09.526'N, 94°22.950'W, 23.vii.1999, D.C. Houghton, 1 female (pinned) (UMSP); Lyon Co., Redwood River, Camden State Park, 44°22.452'N, 95°55.315'W, 06.vi.2000, D.C. Houghton, 1 male (alcohol) (UMSP); same locality, 31.vii.1999, D.C. Houghton, 1 male, 2 females (pinned) (UMSP); McLeod Co., Otter Creek, County Road 01, 44°53.667'N, 94°03.33'W, 22.vi.2001, D.C. Houghton, 2 males (alcohol) (UMSP); Morrison Co., Mississippi River, Charles Lindberg State Park, 45°57.000'N, 94°23.333'W, 24.vi.2000, D.C. Houghton, 2 males (alcohol) (UMSP); Pine Co., Kettle River, Banning State Park, 46°10.000'N, 92°50.000'W, 28.vi.2001, D.C. Houghton, 1 male (alcohol) (UMSP); Pine Co. Kettle River, 9.viii.1991, L.J. Luedeman, 4 males, 2 females (alcohol) (UMSP); Ramsey Co., St. Paul, University Farm, A.A. Granovsky, 20.viii.1935, 1 male (pinned) (UMSP); Rock Co., Rock River, County Road 1, 43°32.666'N, 96°11.917'W, 07.vi.2000, D.C. Houghton, 9 males, 6 females (alcohol) (UMSP); Swift Co., Pomme de Terre River, State Road 12 rest area, 45°17.450'N, 95°58.745'W, 335 m, 29.vii.1999, D.C. Houghton, 7 males, 5 females (pinned) (UMSP); Wright Co., North Fork Crow River, public access, 45°05.500'N, 93°52.000'W, 17.ix.2000, D.C. Houghton, 1 male, 1 female (alcohol) (UMSP); Wright Co., North Fork Crow River, County Road 115, 45°05.500'N, 93°52.333'W, 27.vi.2001, D.C. Houghton, 25 males, 11 females (alcohol) (UMSP); Wright Co., Deer Lake, County Road 108, 45°08.500'N, 93°54.667'W, 27.vi.2001, D.C. Houghton, 2 females (alcohol) (UMSP); Yellow Medicine Co., Yellow Medicine River, confl. Minnesota River, Upper Sioux Agency State Park, 44°44.319'N, 95°25.904'W, 30.vii.1999, D.C. Houghton, 43 males, 30 females (alcohol) (UMSP); Yellow Medicine Co., Minnesota River, Mile 243.9, T115N, R38W, S28, 14.viii.1980, N. Potthoff, 5 males, 20 females (alcohol) (UMSP); Yellow Medicine Co., Minnesota River, picnic area, Upper Sioux Agency State Park, 44°44.393'N, 95°27.306'W, 122 m, 30.vii.1999, D.C. Houghton, 10 males, 1 female (pinned) (UMSP); **New York:** Warren Co., Hudson River, Riparius, 43°39.5'N, 73°53.8'W, 29.vii.1996, C.M. & O.S. Flint, Jr., 2 males (pinned) (NMNH); **Wisconsin:** Polk Co., St. Croix Falls, 29.viii.1937, L.R. Penner, 2 males, 10 females (pinned) (UMSP).

#### Remarks.

Mexican, Central, and South American specimens identified previously as *Oecetis avara* have been transferred to a number of new species in this paper. Some North American material has also been transferred to another new species, *Oecetis houghtoni*. Most North American material examined was from Minnesota, where *Oecetis avara* and *Oecetis houghtoni* co-occur, sometimes at the same location. In Minnesota the 2 species are distinguishable by color attributes and also by minor genitalic characters. Available COI barcode data are consistent with the recognition of 2 species and conform to the morphological differences noted (Fig. 58). Only a limited amount of material from other localities in North America was examined. Some material examined from other localities conformed to the differences noted in the holotypes of the 2 species, and these are included in the materials examined list above, but specimens from other localities could not always be confidently identified. This may either represent variation within the 2 species, or additional unidentified species. A more extensive study of North American material is necessary to determine this, but was outside the scope of the current study. Figs 28 and 29–30 illustrate some of the variation encountered. See also the remarks section under *Oecetis houghtoni*.

The illustration of *Oecetis avara* (Fig. 5) is taken from the holotype specimen. The holotype was reported by Smith and Lehmkuhl (1980) as having the phallic structure badly damaged, but this was not evident in our examination of the holotype. The species has been illustrated by a number of authors, but many of the illustrations vary from the genitalia of the holotype specimen. Although Smith and Lehmkuhl (1980) reported examining the type, the specimen illustrated was apparently a specimen from Saskatchewan determined to be *Oecetis avara*, and not that of the type itself. Due to the minor differences among species in the *Oecetis avara* group, it is usually difficult to state conclusively which species was illustrated by different authors. Only the illustrations provided by Betten (1934) and Moulton and Stewart (1996) conform closely enough to the holotype to be confidently identified as *Oecetis avara*. The illustration by Bueno-Soria (1981) is also similar to the holotype of *Oecetis avara*, but may have been based on *Oecetis verrucula* sp. n., which occurs in Mexico and whose diagnostic ventral wart may have been omitted, since it is a relatively minor feature.

### Oecetis campana sp. n.

http://zoobank.org/4825E24C-A58E-49D7-82AE-BE60C24B01C4

http://species-id.net/wiki/Oecetis_campana

Figs 6 , 51 , Map 4

#### Diagnosis.

*Oecetis campana* sp. n. is similar in size and general coloration to both *Oecetis mexicana* sp. n. and *Oecetis constricta* sp. n. It differs from either of these species in that the phallobase of the male, in lateral view, has its ventral margin nearly straight or very weakly arched (Fig. 6B), and thus more “bell-shaped” in overall appearance. Like *Oecetis mexicana*, the apex of the phallobase, in caudal view, is U-shaped, rather than “pinched.” In coloration it is slightly darker than *Oecetis mexicana*, and has larger forewing spots and more elongate setation along the costal margin. The ventral lobe of the inferior appendage forms an approximate right angle to the dorsal lobe, as compared to the distinctly obtuse angle in *Oecetis constricta*. *Oecetis mexicana* is variable in this respect. *Oecetis campana* is currently only known from a restricted region in Ecuador, but the existence of essentially identical forms from several locations suggests that it represents a distinct species. Based on their illustration, it seems likely that the specimen of *Oecetis avara* illustrated by Rueda Martín et al. (2011) from Bolivia represents this species. Specimens typical of and referable to *Oecetis mexicana* are also found in Ecuador, and at one site were found together with *Oecetis campana*.

#### Adult.

Forewing length: male (6.5–7.9 mm), female (6.5 mm). Color yellowish, about same color as *Oecetis mexicana* sp. n. Antennae whitish, annulations not evident. Forewing spots distinct and large; spot at base of discal cell largest, spots at base of other major wing forks nearly as large; veins of forewing chord widely spaced, either even spaced or *s* and *r-m* veins slightly closer; chord with small spots at juncture of major veins, those at opposite ends of chord larger; apical spots, at apices of major veins prominent, rounded, pigmentation extending beyond veins. Setae along veins in apical part of forewing elongate, semi-prostrate, laterally diverging. Fringe of setae along costal margin of forewing moderately dense, very elongate, nearly erect.

#### Male genitalia.

Segment IX very short, with elongate setae along posterolateral margin. Tergum X with narrow, deflexed mesal lobe, apex of lobe lobulate, with small sensilla; lobe continuous basoventrally with short, paired lateral membranous projections. Preanal appendage moderately elongate, about 3 times maximum width, simple in structure, apical setae elongate. Inferior appendage with prominent rounded dorsal lobe and angularly projecting ventral lobe; ventral lobe moderately projecting, forming approximate 90° angle with dorsal lobe, posterior margin of ventral lobe, as viewed ventrally (Fig. 6E), angularly bent near base, linear or slightly concave from bend to apex of projection; basomesal projection weakly developed, scarcely protruding, with short, stiff setae; dorsal lobe with stout, mesally-curved setae on dorsal margin and stout, ventrally-curved setae on mesal surface. Phallobase very short, tubular, flared apically, ventral apex somewhat projecting, ventral margin, as viewed laterally, nearly straight or very weakly curved; ventral apex, as viewed caudally, U-shaped (Fig. 6C). Phallotremal sclerite prominent, basally forming short tubular collar, ventral margin projecting; asymmetrical lateral sclerite present, broadly rounded, not conspicuous.

#### Holotype.

**Male** (pinned), **ECUADOR: Zamora-Chinchipe:** Río Chicaña, 9 km N Yanzatza, el 880 m, 20.xi.1990, O.S. Flint, Jr. (NMNH) (UMSP000208264).

#### Paratypes.

**ECUADOR: Napo:** Pano, 580 m, 12.ix.1990, O.S. Flint, Jr. 1 male (pinned) (NMNH); **Pastaza:** Puyo, 1–7.ii.1976, Spangler, et al., 1 male (pinned), 1 male, 3 females (alcohol) (NMNH); Puyo, 8–11.ii.1976, Spangler, et al., 1 male, 1 female (alcohol) (NMNH); Puyo, 5.v.1977, P.J. Spangler & D.R. Givens, 1 male (alcohol) (NMNH); Puyo, 27 km N, Est. Fluv. Metrica, 4.ii.1976, Spangler, et al., 1 male (alcohol) (NMNH); **Zamora-Chinchipe:** 6 km E Zumbi, el 980 m, 21.ix.1990, O.S. Flint, Jr., 1 male, 1 female (pinned) (NMNH); Yanzaza, 15.vi.1976, A. Langley, et al., 1 male (pinned) (NMNH); Río Chicaña, 9 km N Yanzatza, el 880 m, 20.xi.1990, O.S. Flint, Jr., 9 males (pinned), 13 males, 5 females (alcohol) (NMNH) (UMSP).

#### Etymology.

This species is named *Oecetis campana*, from the Latin for bell, in reference to the shape of the phallobase in this species, which has something of a bell-shape due to the apex being distinctly flared and the ventral margin very little curved.

### Oecetis constricta sp. n.

http://zoobank.org/10FF58C4-38F0-4455-B8C3-4BCA176943B4

http://species-id.net/wiki/Oecetis_constricta

Figs 7 , 56 , Map 4

#### Diagnosis.

**Figure 7.**
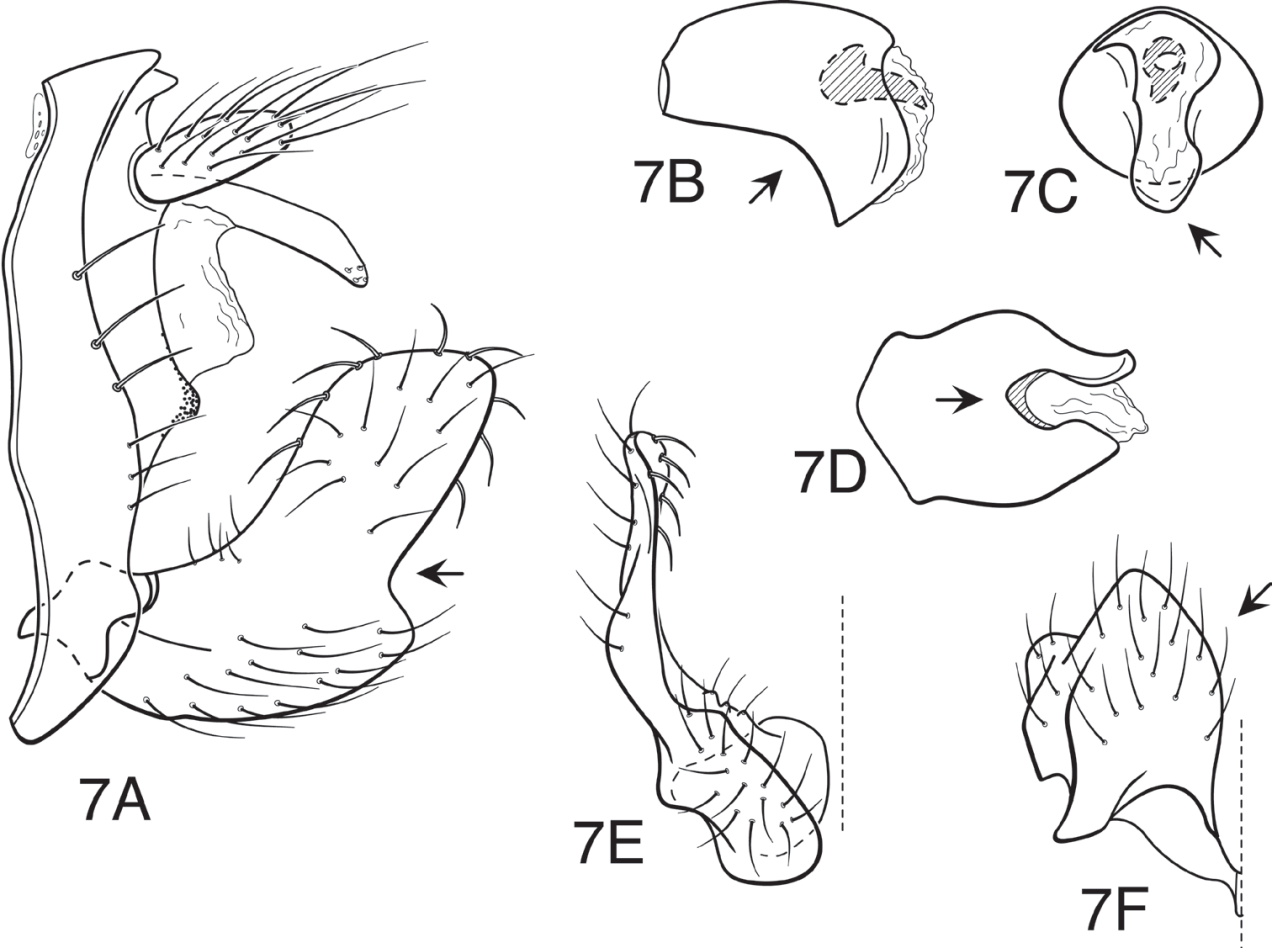
*Oecetis constricta* sp. n., male genitalia: **A** lateral **B** phallic apparatus, lateral **C** phallic apparatus, ventral **D** phallic apparatus, caudal **E** inferior appendage, caudal **F** inferior appendage, ventral.

This species resembles *Oecetis mexicana* sp. n. and *Oecetis campana* sp. n., in its small size, yellow coloration, prominent and distinctly rounded forewing spots, and in the obtuse angle formed between the ventral and dorsal lobes of the inferior appendage. The character most useful in diagnosing this species is the distinctive constricted, or “pinched” apex of the phallobase, as viewed caudally (Figs 7C–D). It should be noted, however, that in some exceptional specimens assigned to *Oecetis mexicana* from Panama the apex of the phallobase was rather narrowly U-shaped (Fig. 19C). It appears that the pinched apex of the phallobase in *Oecetis constricta* is formed, in part, by the asymmetrical phallic sclerite forming a part of the marginal wall of the phallobase, and thus the sclerite is not as projecting as it is in *Oecetis mexicana*. An additional population from Costa Rica (assigned to *Oecetis constricta* and listed below under additional material examined) seems to be somewhat intermediate in characters between *Oecetis mexicana* and *Oecetis constricta*; it lacks the distinctive pinched apex of *Oecetis constricta*, but agrees in other details. It was not included in the paratype material due to its aberrant characters. The COI barcode of a specimen from this series was similar to other specimens of *Oecetis constricta*; however, no barcode sequences have yet been obtained for *Oecetis mexicana*. It is possible that there is some hybridization or introgression occurring between the 2 species and it is also possible that the specimens mentioned may have been misassigned (see remarks section following species description). In general, the 2 species seem to be distinct and their distributions overlap significantly. In a large enough series, it can be noted that *Oecetis constricta* is slightly darker in color than either *Oecetis mexicana* or *Oecetis campana*. Setae on the costal margin of the forewing are slightly longer than in *Oecetis mexicana* and shorter than in *Oecetis campana*. Other differences from *Oecetis mexicana* include its slightly smaller size and a tendency for the phallobase to be more angularly bent ventrally. The ventral lobe of the inferior appendage is always clearly obtusely angled in relation to the dorsal lobe. An additional difference between *Oecetis constricta* and the other 2 species is that the mesal margins of the inferior appendages, in ventral view, tend to be less angularly diverging (compare Fig. 7F to Figs 6E and 18F).

#### Adult.

Forewing length: male (6.6–7.8 mm), female (5.5–6.5 mm). Color yellowish with slight brownish cast, slightly darker than *Oecetis mexicana* sp. n. Antennae pale yellow with indistinct, narrow annulations at intersection of antennomeres. Forewing spots distinct and large; spots at base of discal and thyridial cells largest, spots at base of other major wing forks nearly as large; veins of forewing chord widely and usually nearly evenly spaced, with crossveins nearly perpendicular, or *s* and *r-m* veins slightly closer; chord with small spots at juncture of major veins, those at opposite ends of chord larger; apical spots, at apices of major veins prominent, pigmentation extending onto the membrane. Setae on veins in apical part of forewing, semi-prostrate, laterally splayed, and diverging. Fringe of setae along costal margin of forewing moderately dense, elongate, nearly erect.

#### Male genitalia.

Segment IX very short, with elongate setae along posterolateral margin. Tergum X with narrow, deflexed mesal lobe, apex of lobe lobulate or slightly tapered apically, with small sensilla; lobe continuous basoventrally with short, paired lateral membranous projections. Preanal appendage moderately elongate, length about 3 times maximum width, simple in structure, apical setae elongate. Inferior appendage with prominent rounded dorsal lobe and angularly projecting ventral lobe; ventral lobe moderately projecting, forming distinctly obtuse angle with dorsal lobe, posterior margin of ventral lobe, as viewed ventrally (Fig. 7F), gradually curved outward, not strongly bent at base; basomesal projection weakly developed, scarcely or not protruding, with short, stiff setae; dorsal lobe with stout, mesally-curved setae on dorsal margin and stout, ventrally-curved setae on mesal surface. Phallobase short and tubular, ventral apex distinctly, rather angularly down curved; ventral apex, as viewed caudally, distinctively “pinched” and narrowed, apex U-shaped (Fig. 7C). Phallotremal sclerite prominent, basally forming short tubular collar, ventral margin projecting, apex acute; asymmetrical lateral sclerite present, but may not be evident because it forms lateral wall of “pinched” apex.

#### Holotype.

**Male** (pinned), **COSTA RICA: San José:** Res. Biol. Carara, Río Carara in Carara, 9°46.680'N, 84°31.860'W, el 200 m, 14.iii.1991, Holzenthal, Muñoz, Huisman (UMSP) (UMSP000207959).

#### Paratypes.

**MEXICO: Chiapas:** Puente Arroyo Viejo, Rt 200, km 141, 9.vi.1967, Flint & Ortiz, 1 male, 2 females (pinned) (NMNH); **Veracruz:** Almilmga, nr. El Palmar, Río Tonto, 1.iv.1963, F. Pacheco, 10 males, 4 females (alcohol) (NMNH); **GUATEMALA:** Río Matapa, 10 km SE Esquintla, el 275 m, 5–6.iii.1970, E.J. Fee, 1 male (alcohol) (NMNH); **Suchetepequez:** Puente Ixtacapa, 18–19.vi.1966, Flint & Ortiz, 1 male, 3 females (pinned) (NMNH); **HONDURAS:** El Zamorano, 28–29.i.1966, G.F. Freytag, 1 male, 3 females (alcohol) (NMNH); **NICARAGUA:** Puente Quinama, E Villa Somoza, 29.vii.1967, O.S. Flint, Jr., 1 male, 1 female (pinned) (NMNH); **COSTA RICA:** Pedregoso, 21.ii.--, D.L. Rounds, 1 male (pinned) (NMNH); Las Canas, 13.vii.1965, P.J. Spangler, 1 male (alcohol) (NMNH); **Guanacaste:** P. N. (Parque Nacional) Guanacaste, Maritza, Río Tempisquito, 10°57.480'N, 85°29.820'W, el 550 m, 19–20.vii.1987, Holzenthal, Morse, Clausen, 1 male (pinned) (UMSP); El Hacha, Quebrada Pedregal, 10°58.980'N, 85°32.340'W, el 300 m, 27.vii.1987, Holzenthal, Morse, Clausen, 1 male (pinned) (UMSP); Río Tizate, 7.2 km NE Canas Dulces, 10°46.380'N, 85°26.940'W, el 275 m, 28.vi.1986, Holzenthal, Heyn, Armitage, 1 male (pinned), 2 males, 1 female (alcohol) (UMSP); **Heredia:** La Selva Field Station nr. Puerto Viejo, 21–28.iii.1988, W. E. Steiner, J. M. Hill, J. M Swearingen & J. M. Mitchell, 1 male, 4 females (pinned) (NMNH); Est. Biol. La Selva, Río Puerto Viejo, 10°26.400'N, 84°0.720'W, 30 m, 10–11.ii.1986, Holzenthal, 1 male (alcohol) (UMSP); **Limón:** Río Uatsi, ca. 8 km (air) W Bribri, 9°37.200'N, 82°54.000'W, el 60 m, 25.iii.1987, Holzenthal, Hamilton, Heyn, 1 male (pinned) (UMSP); **Puntarenas:** Res. Biol. Carara, Río Carara, 4.3 km (rd) E Costanera Sur, 9°48.600'N, 84°34.320'W, el 20 m, 12.iii.1991, Holzenthal, Muñoz, Huisman, 6 males, 7 females (pinned), 3 males, 14 females (alcohol) (UMSP); Res. Biol. Carara, Quebrada Bonita, 9°46.500'N, 84°36.300'W, el 35 m, 11.iii.1991, Holzenthal, Muñoz, Huisman, 11 males, 2 females (pinned) (UMSP); same location, 18–20.v.1990, Holzenthal & Blahnik, 7 males, 7 females (pinned), 2 males, 3 females (alcohol) (UMSP); Quebrada Pita, ca. 3 km (air) W Golfito, 8°38.520'N, 83°11.580'W, el 15 m, 15.ii.1986, Holzenthal, Morse, Fasth, 2 males, 3 females (alcohol) (UMSP); Rio Jaba at rock quarry, 1.4 km (air) W Las Cruces, 8°47.400'N, 82°58.200'W, el 1150 m, 15.iii.1991, Holzenthal, Muñoz, Huisman, 1 male, 5 females (alcohol) (UMSP); Parque Nacional Corcovado, Est. Sirena, Río Camaronal, 8°28.860N, 83°35.640'W, el 5 m, 12–13.iv.1989, Holzenthal & Blahnik, 1 male (alcohol) (UMSP); **San José:** same data as holotype, 15 males, 5 females (pinned) (UMSP); Res. Biol. Carara, Río del Sur, 1.5 km (rd) S Carara, 9°46.140'N, 84°31.860'W, el 160 m, 13.iii.1991, Holzenthal, Muñoz, Huisman, 3 males (pinned), 6 males, 6 females (alcohol) (UMSP); Res. Biol. Carara, Río Carara in Carara, 9°46.680'N, 84°31.860'W, el 200 m, 14.iii.1991, Holzenthal, Muñoz, Huisman, 12 males, 12 females (alcohol) (UMSP); Río Negro, 3.5 km SE jct. route 239, 9°40.800'N, 84°23.640'W, el 230 m, 21.iii.1986, Holzenthal & Fasth, 3 males, 1 female (pinned), 8 males, 11 females (alcohol) (UMSP); **PANAMA: San Blas:** Quebrada Pingadi, 9 km N Nusaganda, 1–2.iii.1995, Flint & Louton, 6 males, 5 females (pinned), 1 male, 3 females (alcohol) (NMNH); **COLOMBIA: Antioquia:** Quebrada Honda, 12 km SW Fredonia, el 1450 m, 22.ii.1983, O. S. Flint, Jr., 1 male, 1 female (pinned) (NMNH); **Magdalena:** Municipio de Santa Marta, Río Minca en Minca, 11°8.584'N, 74°6.967'W, el 570 m, 9.xii.1997, F. Muñoz-Q, et al., 2 males (pinned) (UMSP); **Tolima:** Armero nr. Guayabal, 2–10.ii.1977, E.L. Peyton, 28 males, 7 females (alcohol); **ECUADOR: Cotopaxi:** Quevedo, 36 km NE, el 305 m, 21.vii.1976, J. Cohen, 4 males, 3 females (alcohol) (NMNH); **Guayas:** Sta. Elena, 47.6 km N, el 37 m., 14.vii.1975, J. Cohen, 1 male (alcohol) (NMNH); **Napo:** Pano, el 580 m, 12.ix.1990, O. S. Flint, Jr., 1 male, 2 females (pinned) (NMNH); **VENEZUELA: Sucre:** Río Cocollar, 1.5 km SE Las Piedras de Cocollar, 10°09.617'N, 63°47.605'W, el 810 m, 7–8.iv.1995, Holzenthal & Flint, 2 males (pinned) (UMSP) (NMNH); **Zulia:** Río Yasa, ca. 3 km (air) E Kasmera (Est. Biológica), 9°56.460'N, 72°43.200'W, el 150 m, 14.i.1994, Holzenthal, Cressa, Rincón, 3 males, 1 female (pinned), 2 males, 4 females (alcohol) (UMSP) (IZAM); Caño Carichuano, 3.4 km SE Carbones del Guasare, 11°0.120'N, 72°17.100'W, el 70 m, 12–13.i.1994, Holzenthal, Cressa, Rincón, 2 males (pinned) (UMSP); Parque Nacional Perijá, Río Negro in Toromo, 10°3.060'N, 72°42.720'W, el 360 m, 15.i.1994, Holzenthal, Cressa, Rincón, 1 male, 1 female (pinned), 1 male, 1 female (alcohol) (UMSP); **TRINIDAD:** Arima River, Verdant Vale, 10°41'N, 61°18’ W, el 170 m, 19.vi.1993, N.E. Adams & W.N. Mathis, 6 males, 1 female (pinned), 11 females (alcohol) (NMNH); Yarra River, Filette (1km SE), 10°47'N, 61°21'W, 25.vi.1991, O.S. Flint, Jr., 6 males (pinned) (NMNH); Blue Basin Waterfall, 10°44'N, 61°32'W, el 120 m, 21.vi.1993, 5 males, 1 female (pinned), 2 males, 3 females (alcohol) (NMNH); Blue Basin River, 10°44'N, 61°32'W, el 100 m, 21.vi.1993, N.E. Adams & W.N. Mathis, 2 males, 12 females (alcohol) (NMNH); Tacarigua River, Caura Rec. area, 10°43'N, 61°17'W, 22.vi.1993, O.S. Flint & N.E. Adams, 2 males, 3 females (pinned), 4 males, 7 females (alcohol) (NMNH); below Maracas Falls, 10°44'N, 61°24'W, el 250 m, 18.vi.1993, N.E. Adams & W.N. Mathis, 1 female (pinned) (NMNH); **GUYANA:** Kumu, 25 km SE Lethem, 3°15.9'N, 59°43.6'W, 4–5.iv.1994, O.S. Flint, Jr., 1 male (pinned) (NMNH).

#### Additional material examined.

**COSTA RICA: San José:** Res. Biol. Carara, Río del Sur, 1.5 km (rd) S of Carara, 9°46.140'N, 84°31.860'W, el 160 m, 13.iii.1991, Holzenthal, Muñoz, Huisman, 16 males, 12 females (pinned) (UMSP). These specimens are somewhat intermediate with *Oecetis mexicana*, as discussed above, and it is possible that they have been misassigned.

#### Etymology.

This species is named *Oecetis constricta* because of the constricted apex of the phallobase in this species.

#### Remarks.

The series listed above as additional material examined and discussed in the diagnosis was from Carara in Costa Rica, near where other specimens typical of *Oecetis constricta* were found. It is possible that this population owes its intermediacy in genitalic characters to some degree of introgression from *Oecetis mexicana*. An alternate explanation for this intermediacy could be method of clearing that was used on these specimens. They were cleared by use of the lactic acid method in the later part of the study; the heat used in this method may have introduced some degree of distortion to the apex of the phallobase (causing it to appear U-shaped, rather than distinctly pinched or constricted). This was never a factor in the many specimens of *Oecetis constricta* or *Oecetis mexicana* cleared with cold (room temperature) KOH.

### Oecetis disjuncta

(Banks)

http://species-id.net/wiki/Oecetis_disjuncta

**Figure 8.**
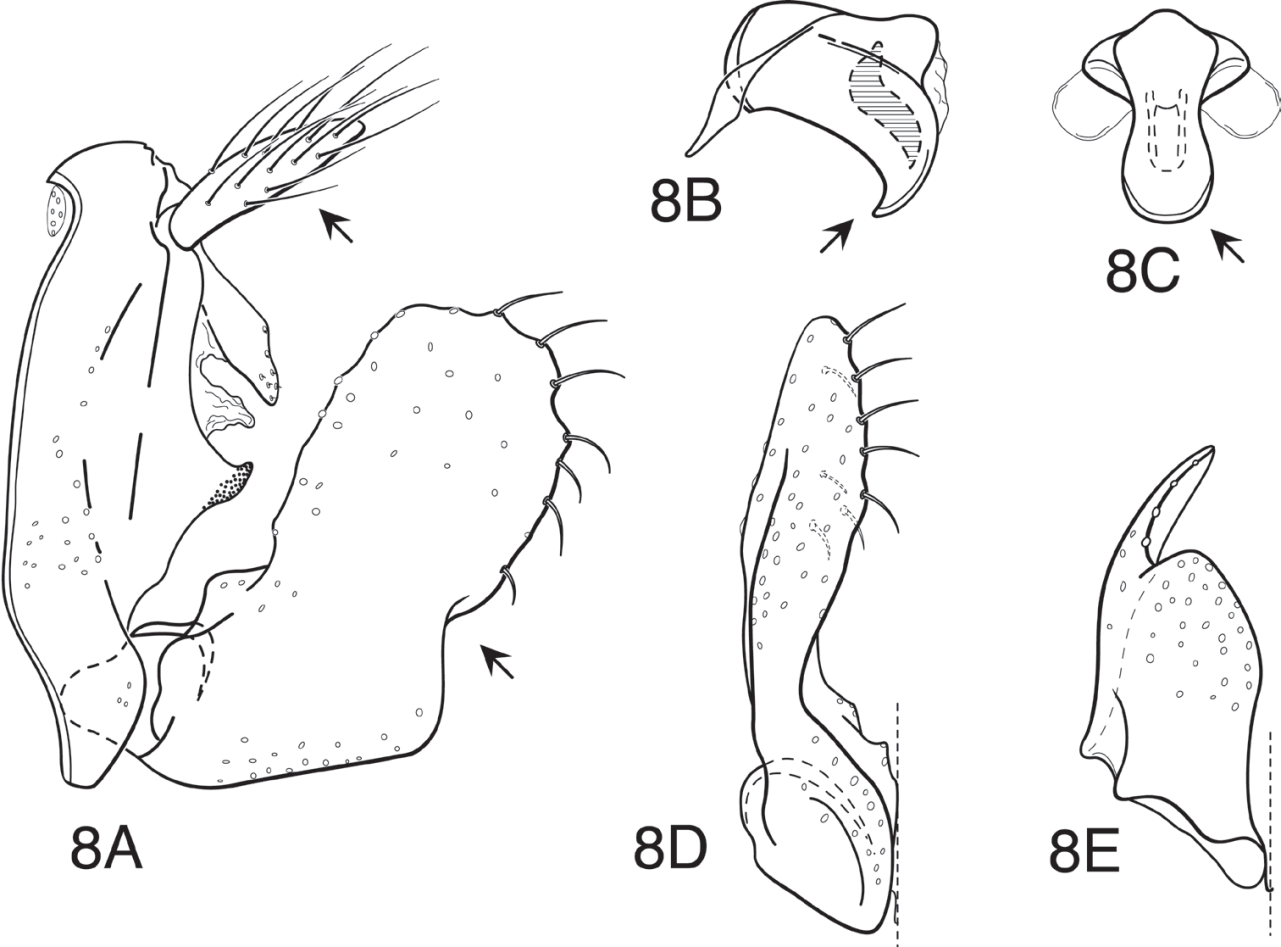
*Oecetis disjuncta* (Banks), Holotype, male genitalia: **A** lateral **B** phallic apparatus, lateral **C** phallic apparatus, caudal **D** inferior appendage, caudal **E** inferior appendage, ventral.

Figs 8 , 31 , 39 , Map 2

Oecetina disjuncta Banks, 1920: 351 (pl. 7, fig. 100) [holotype male, California, Arroyo Seco Canyon, San Gabriel Mts., Switzer’s Camp, June 17, MCZ type 10915].
Oecetodes disjuncta (Banks): Milne 1934: 17, 19.
Oecetis disjuncta (Banks): Ross 1938: 24; Denning 1956: 265 [as possible synonym of *Oecetis avara*]; Smith and Lehmkuhl 1980: 638 (Figs 1–2, 5, 7, 9–10, 12, 14, 16, 18, 21, 23, 25–26, 29–34, 37–38, 40–41, 43, 45, 46–48, 50) [adult, larva, pupa, case, distribution – Figs are of *Oecetis sordida* sp. n. and not *Oecetis disjuncta*]; Floyd 1995: 29 (fig. 22A-H, map 25) [larva, case, distribution – figure and all, or most, records undoubtedly of *Oecetis sordida*, and not *Oecetis disjuncta*].

#### Diagnosis.

This species differs from the form that has most often been attributed to it, here described as a new species, *Oecetis sordida*. The 2 are very similar and it is not surprising that Smith and Lehmkuhl (1980) came to the conclusion that the specimens they had examined from Saskatchewan represented this species after examining the holotype. They did note, however, the variation in the length of the preanal appendages, one useful diagnostic character for distinguishing the species. We have examined only a few specimens from California and Oregon that correspond to the holotype of *Oecetis disjuncta*, probably primarily due to the paucity of material from this region in the collections surveyed. Nevertheless, the character differences from *Oecetis sordida* seem to be consistent. It is true that the genitalia in these 2 forms are very similar, with the dorsal and ventral lobes of the inferior appendage merely “notched”, rather than with the ventral lobe distinctly projecting, and with the ventral margin of the phallobase strongly deflexed, and also with an asymmetrical phallic sclerite absent. However, the holotype of *Oecetis disjuncta*, which was described from California, is much lighter in color (a uniform pale yellowish-brown) than any specimens of *Oecetis sordida* (dark brown with a distinctly pigmented chord), is somewhat smaller in size, and has the preanal appendages distinctly more elongate. Additionally, *Oecetis disjuncta* lacks the distinctive rugose ventral apex of the phallobase that seems to consistently occur in *Oecetis sordida*. *Oecetis sordida* is nearly invariant in coloration and morphology throughout its very extensive range (southern Mexico and throughout the western United States, apparently as far east as Michigan). Despite the very limited material examined for *Oecetis disjuncta*, its separate species status is suggested by the distinct COI barcode difference of a specimen from Oregon identified as *Oecetis disjuncta*, as compared to *Oecetis sordida* and *Oecetis avara* (Fig. 58). Our decision to restrict the definition of *Oecetis disjuncta* to only those forms exactly resembling the holotype was made to stabilize the nomenclature of the taxon and to prevent its confusion with the form described here as *Oecetis sordida*. It should be pointed out that *Oecetis disjuncta* also closely resembles *Oecetis apache* sp. n., in the general structure of the genitalia, coloration, and lack of forewing pigmentation. The latter species differs in having the apex of phallobase distinctly V-shaped in caudal view (i.e., its ventral apex is strongly “keeled,” rather than U-shaped), and is also larger and has slightly broader forewings.

#### Adult.

Forewing length: male (9.7–10.2 mm). Color (holotype and California specimen) yellowish-brown, (Oregon specimens) light brown. Antennae whitish with indistinct, narrow annulations at intersection of antennomeres. Forewing spots absent or nearly so; chord nearly unpigmented in holotype, lightly pigmented in specimens from Oregon. Forewing chord with crossveins moderately spaced, diagonal or perpendicular, either nearly evenly spaced or with *s* and *r-m* veins slightly closer. Forewings moderately setose, nearly uniformly along length, setae along veins in apical part of forewing not denser than on wing membrane, not conspicuously laterally diverging. Fringe of setae along costal margin of forewing dense, short, not strongly projecting.

#### Male genitalia.

Segment IX very short, with elongate setae along posterolateral margin. Tergum X with narrow, deflexed mesal lobe, lobe short, tapering apically, apex with small sensilla; lobe continuous basoventrally with paired lateral membranous projections. Preanal appendage elongate, length about 5 times maximum width, simple in structure, apical setae elongate. Inferior appendage with prominent rounded dorsal lobe and scarcely projecting ventral lobe, separated from dorsal lobe by shallow, rounded notch; posterior margin of ventral lobe, as viewed ventrally (Fig. 8E), with rounded bend near base, lobes very weakly diverging; basomesal projection of appendage forming weakly to moderately developed projection with short, stiff setae; dorsal lobe with stout, mesally-curved setae on dorsal margin and ventrally-curved setae on mesal surface (entire surface nearly denuded in holotype). Phallobase relatively short, ventral apex strongly, angularly deflexed, bend close to apex, apex distinctly sclerotized, but not rugose; ventral apex, as viewed caudally, U-shaped (Fig. 8C). Phallotremal sclerite prominent, basally forming relatively large tubular collar, ventral margin projecting; asymmetrical lateral sclerite absent.

#### Material examined.

**USA: California:** Arroyo Seco Canon, San Gabriel Mts. Switzer’s camp, 17.vi--, male (holotype 10915) (MCZ); Turner, East Lake (locality not found), Rec. 21.vi.1883, 1 male (pinned) (NMNH); **Oregon:** Douglas Co., Umpqua National Forest, Steamboat Creek @ Forest Road 38, 5.4 mi NE Hwy 138, 43°22.583'N, 122°39.203'W, 485 m, 15.viii.2006, Holzenthal et al., 2 males (UMSP).

### Oecetis elata

Denning & Sykora

http://species-id.net/wiki/Oecetis_elata

Figs 9 , 43 , Map 3

Oecetis elatus [sic] Denning & Sykora, 1966: 1225 [holotype male: Mexico, Veracruz, Almilinta (locality not found), CAS].
Oecetis elata Denning & Sykora: Flint et al. 1999: 135.

#### Diagnosis.

**Figure 9.**
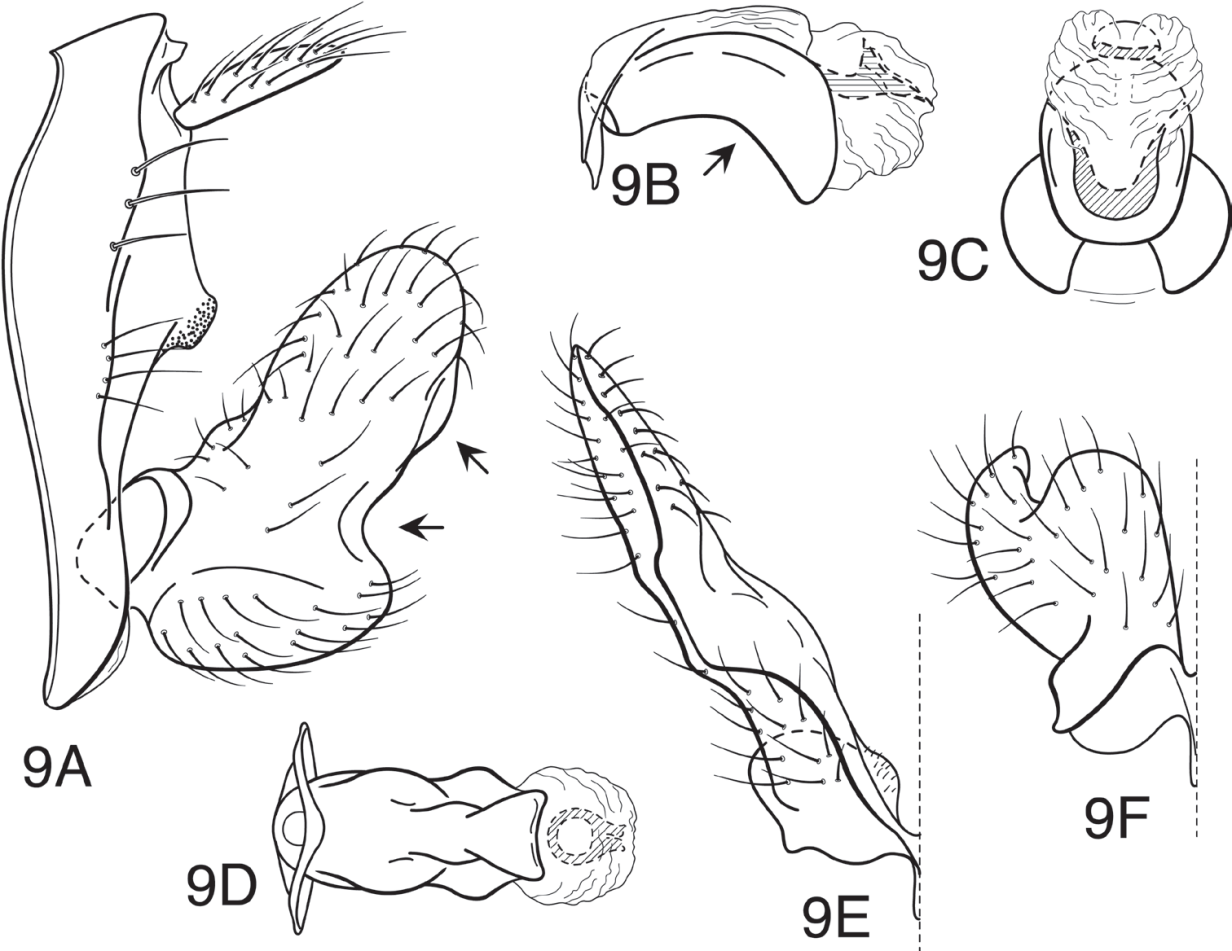
*Oecetis elata* Denning & Sykora, male genitalia: **A** lateral **B** phallic apparatus, lateral **C** phallic apparatus, caudal **D** phallic apparatus, ventral **E** inferior appendage, caudal **F** inferior appendage, ventral.

*Oecetis elata* is readily distinguished from any other species in the *Oecetis avara* group by its small size and dark coloration. As viewed through a stereomicroscope, the body color is almost black and the wings dark brown, giving the species a blackish or fuscous color as viewed generally. In genital morphology *Oecetis elata* is probably most similar to *Oecetis sordida*. However, the apex of the phallobase, while being distinctly and strongly deflexed, is flexed more nearly in the middle. Like *Oecetis sordida*, the basal sclerites anchoring the phallobase, the phallocrypt, are very heavily pigmented and distinctly visible (Fig. 9C). However, *Oecetis elata* is much smaller in size than *Oecetis sordida* and the 2 are not likely to be confused. The angular process on the dorsal lobe of the inferior appendage, as figured and mentioned in the original species description by Denning and Sykora, is somewhat misleading as a diagnostic character. The dorsal lobe of the inferior appendage is actually broadly rounded, as in other members of the *Oecetis avara* group, but the posterior margin is noticeably folded inward, sometimes abruptly enough to give the margin of the appendage, in lateral view, the appearance of being angular. This character, while evident in the type specimen, is not generally characteristic of the species. The apparent absence of a mesal lobe of tergum X (in material examined) is unusual for species in the *Oecetis avara* group and may also prove to be diagnostic.

#### Adult.

Forewing length: male (7.3–7.8 mm). Color medium brown (dark brown as viewed without magnification). Antennae light brown. Forewings without spots, veins of forewing chord not contrastingly pigmented, chord moderately spaced, *s* and *r-m* veins generally rather closely spaced, *m* widely spaced. Setation of forewings relatively uniform; setae along veins in apical part of forewing mostly apically directed, not or only slightly laterally diverging Fringe of setae along costal margin of forewing dense, short, not strongly projecting.

#### Male genitalia.

Segment IX very short, with elongate setae along posterolateral margin. Tergum X with mesal lobe absent (in specimens examined), but with short, paired lateral membranous projections (not figured). Preanal appendage moderately elongate, length about 4 times maximum width, simple in structure, some apical setae elongate. Inferior appendage with prominent rounded dorsal lobe and weakly projecting ventral lobe; dorsal lobe with posterior margin distinctly, but rather weakly, mesally incurved; projection of ventral lobe rounded apically, obtusely angled; ventral margins of ventral lobes, as viewed ventrally (Fig. 9F), not diverging at base, rounded apically; basomesal projection of appendage scarcely projecting, with few stiff setae; dorsal lobe with stout, mesally-curved setae on dorsal margin and stout, ventrally-curved setae on mesal surface. Phallic shield, surrounding phallobase, very distinctly sclerotized. Phallobase relatively short, strongly ventrally curved at about middle, apex distinctly sclerotized; ventral apex, as viewed caudally, broadly U-shaped. Phallotremal sclerite prominent, basally forming short tubular collar, ventral margin projecting, apex acute; asymmetrical lateral sclerite absent.

#### Material examined.

**MEXICO: Puebla:** L. Alchichicha, 19–20.vii.1965, Flint & Ortiz, 29 males (pinned) (NMNH).

### Oecetis houghtoni sp. n.

http://zoobank.org/71BA2802-19D7-448F-BDEB-52B6EDF09583

http://species-id.net/wiki/Oecetis_houghtoni

**Figure 10.**
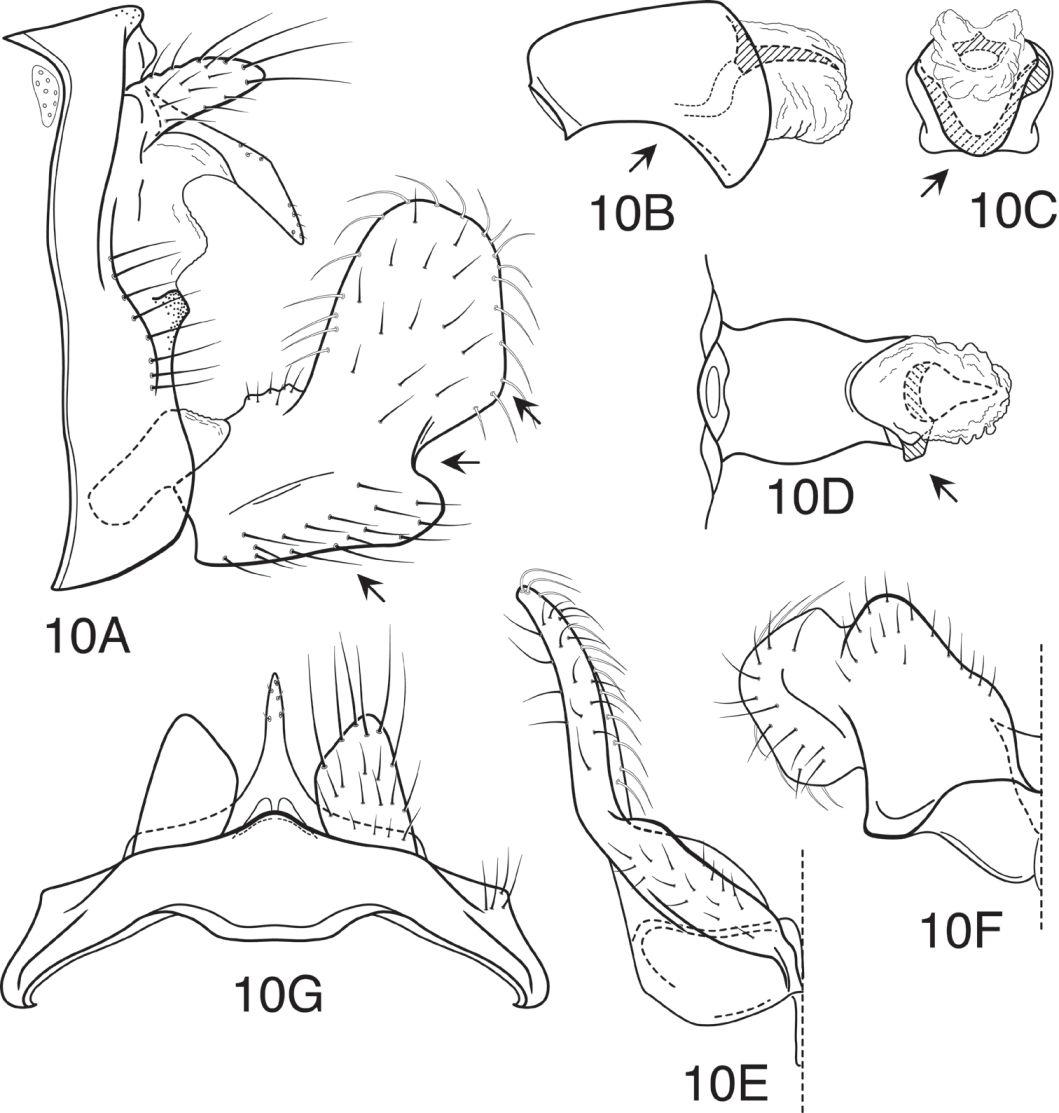
*Oecetis houghtoni* sp. n., male genitalia: **A** lateral **B** phallic apparatus, lateral **C** phallic apparatus, caudal **D** phallic apparatus, ventral **E** inferior appendage, caudal **F** inferior appendage, ventral **G** segment IX and tergum X, dorsal.

**Figures 11–14.**
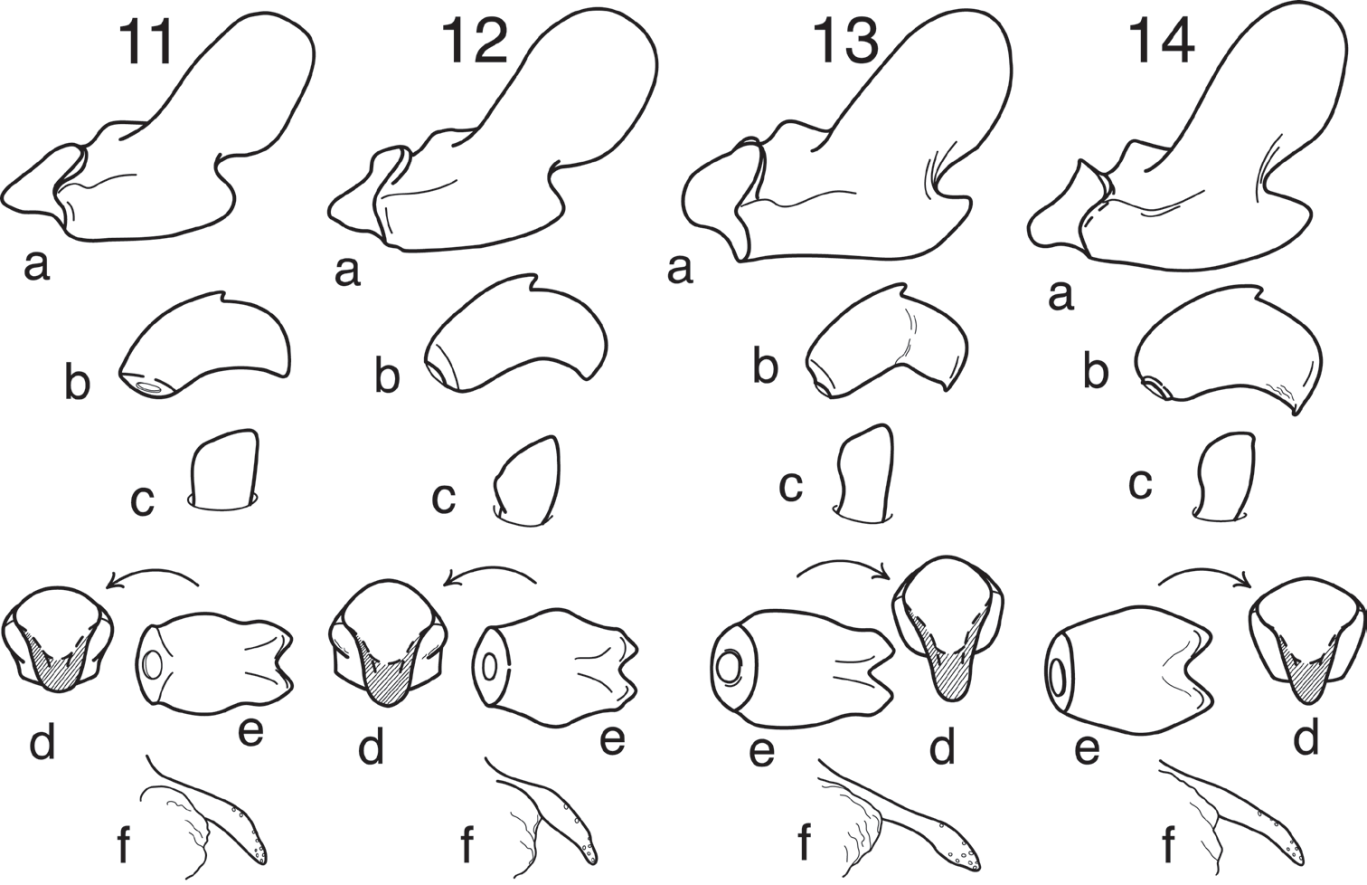
Variation in Minnesota specimens of *Oecetis houghtoni* sp. n., and *Oecetis avara* (Banks). **11–12** *Oecetis houghtoni* **13–14** *Oecetis avara*: **a** inferior appendage, lateral **b** phallobase, lateral **c** preanal appendage, dorsal **d** phallobase, caudal **e** phallobase, ventral **f** mesal process of tergum X, lateral.

Figs 10 , 11 , 12 , 34 , 42 , Map 2

#### Diagnosis.

*Oecetis houghtoni* is most similar to, and most likely to be confused with, *Oecetis avara*. At least in material from Minnesota it is distinctly darker in color, brownish, rather than yellowish or yellowish-brown, and has less distinct forewing spots (sometimes nearly absent). The 2 species can generally be identified by color attributes alone. However, there are other differences. In general, the ventral lobe of the inferior appendage is less projecting than in *Oecetis avara* and the angle it forms with the dorsal lobe tends to be somewhat rounded, without the “wrinkling” apparent in *Oecetis avara*, and the posterior margin of the dorsal lobe is somewhat angular. The latter character is subtle, but once noticed is very useful. The angle between the dorsal and ventral lobes is usually slightly acute or nearly at a right angle, and thus may be difficult to distinguish from the distinctly acute angle of *Oecetis avara*. The phallobase in *Oecetis houghtoni* is arched ventrally, but usually not as strongly deflexed apically as in *Oecetis avara* (this character is variable in both species). Also, the phallobase tends to be more uniformly sclerotized (not as sclerotized apically). In most specimens examined, the forewing chord is more narrowly spaced in *Oecetis houghtoni* than in *Oecetis avara*, often with the veins more or less diagonal (Fig. 34A), or with the *s* and *r-m* veins very closely spaced. *Oecetis houghtoni* also differs significantly from *Oecetis avara* in COI barcode sequence (Fig. 58). The possibility that this difference may represent unrecognized species diversity had already been commented on by Zhou et al. (2011).

#### Adult.

Forewing length: male (8.0–10.0 mm), female (6.2–8.3 mm). Color light brown (darker than *Oecetis avara*). Antennae whitish with indistinct, narrow annulations at intersection of antennomeres. Forewing spots very small and indistinct; spots at base of discal and thyridial cells and base of fork V usually evident; veins of forewing chord narrowly spaced, usually more or less diagonal, either evenly spaced (Fig. 34A) or *s* and *r-m* veins slightly closer; chord with crossveins lightly pigmented; spots at apices of major veins either absent or very indistinct (usually not evident). Setae along veins in apical part of forewing mostly apically directed, not or only slightly laterally diverging. Fringe of setae along costal margin of forewing dense, short, not strongly projecting.

#### Male genitalia.

Segment IX very short, with elongate setae along posterolateral margin. Tergum X with narrow, deflexed mesal lobe, apex of lobe tapered, with small sensilla; lobe continuous basoventrally with short, paired lateral membranous projections. Preanal appendage relatively short, length about 2 times maximum width (shorter, on average, than *Oecetis avara*), simple in structure, apical setae elongate. Inferior appendage with prominent rounded dorsal lobe and angularly projecting ventral lobe; projection of ventral lobe only moderately protruding, typically forming a somewhat acute angle with dorsal lobe, but varying to a somewhat obtuse angle, angle usually rounded, not noticeably “wrinkled’ at articulation; posterior margin of dorsal lobe characteristically angulate and somewhat mesally infolded; mesal margin of ventral lobe, as viewed ventrally (Fig. 10F), strongly bent near base; basomesal projection of appendage weakly rounded, with short, stiff setae; dorsal lobe with stout, mesally-curved setae on dorsal margin and stout, ventrally-curved setae on mesal surface. Phallobase very short, tubular basally, ventral margin curved, apex not strongly down curved, only moderately sclerotized; ventral apex, as viewed caudally, U-shaped (Fig. 10C). Phallotremal sclerite prominent, basally forming short tubular collar, ventral margin projecting, apex acute; asymmetrical lateral sclerite present.

#### Holotype.

**Male** (pinned), **USA: Minnesota:** Lake Co., Baptism River at State Hwy. 1, Eckbeck campground, 47°22.137'N, 91°13.767'W, 320 m, 24.vii.1996, Holzenthal & Huisman (UMSP) (UMSP000050387).

#### Paratypes.

**CANADA: Ontario:** Ottawa, Ottawa Champlain Rapids, 6.vii.1978, C.M. & O.S. Flint, Jr. 1 male (pinned) (NMNH); Ottawa River, 5 mi N Dunrobin, 45°28.1'N, 75°58.8'W, 25.vii.1996, C.M. & O.S. Flint, Jr., 1 male (pinned) (NMNH); Kearney, Magnetawan River, 23.viii.1983, O.S. Flint, Jr., 1 male (pinned) (NMNH); Killaloe, Brennans Creek, 18.viii.1983, O.S. Flint, Jr., 1 male (pinned) (NMNH); **USA: Arkansas:** Montgomery Co., Little Missouri River, Albert Pike, 28.viii.1980, E.J. Bacon, 15 males (alcohol) (UMSP); same locality, 19.ix.1980, E.J. Bacon, 1 male, 17 females (alcohol) (UMSP); same locality, 20.ix.1980, E.J. Bacon, 1 male, 6 females (alcohol) (UMSP); same locality, 24.ix.1981, E.J. Bacon, 53 males, 85 females (alcohol) (UMSP); **Michigan:** Cedar River, 25.viii.1937, L.R. Penner, 1 male (pinned) (UMSP); **Minnesota:** Aitkin Co., Snake river, Silver Star Road, 46°12.000'N, 93°15.000'W, 29.vi.2001, D.C. Houghton, 263 males, 287 females (alcohol) (UMSP); Aitkin Co., Swan River, nr. State Road 200, 47°00.5000'N, 93°15.500'W, 12.vii.2000, D.C. Houghton, 317 males, 302 females (alcohol) (UMSP); Aitkin Co., 15.vii.1938, D.G. Denning, 1 male (pinned) (UMSP); Anoka Co., Coon Creek, 13.vi.1937, D.G. Denning, 1 male, 3 females (pinned) (UMSP); Anoka Co., 27.vi.1938, D.G. Denning, 1 female (pinned) (UMSP); Becker Co., Pike Lake, Elbow Lake Road, 47°07.000'N, 95°31.333'W, 04.vii.2000, D.C. Houghton, 1 male (alcohol) (UMSP); Becker Co., Ottertail River, mi. 162, Hubbel Pond WMA, 16.viii.1982, N. Kirsch, 2 males, 7 females (alcohol) (UMSP); Beltrami Co., Shotley Bridge, County Road 111, 48°03.500'N, 94°31.667'W, 04.vii.2000, D.C. Houghton, 3 males, 1 female, (alcohol) (UMSP); Beltrami Co., Upper Red Lake, County Road 108, public access, 48°03.917'N, 94°42.167'W, 25.viii.2000, D.C. Houghton, 1 male, 1 female (alcohol) (UMSP); Carlton Co., Silver Creek, Jay Cooke State Park, 46°38.666'N, 92°21.000'W, 14.vii.2001, D.C. Houghton, 2 males, 2 females (alcohol) (UMSP); Cass Co., Big Rice Lake, State Road 06, 46°59.500'N, 93°57.333'W, 30.vi.2000, D.C. Houghton, 2 males (alcohol) (UMSP); Cass Co., Boy River, County Road 53, 47°04.667'N, 94°06.333'W, 30.vi.2000, D.C. Houghton, 319 males, 206 females (alcohol) (UMSP); Cass Co., Mabel Lake, Mabel Lake campground, 47°03.500'N, 94°04.000'W, 30.vi.2000, D.C. Houghton, 5 males, 7 females (alcohol) (UMSP); Cass Co., Shingobee River, County Road 83, 47°00.666'N, 94°40.000'W, 30.ix.2000, M.P. Monson, 11 males, 7 females (alcohol) (UMSP); Cass Co., Birch Bridge, County Road 132, 47°02.000'N, 93°52.000'W, 30.vi.2000, D.C. Houghton, 1 female (alcohol) (UMSP); Cass Co., Cass Lake, 30.vii.1935, R.H. Nagel, 1 female (alcohol) (UMSP); Cass Co., Cass Lake, 10.vii.1936, R.H. Daggy, 1 male (pinned) (UMSP); same locality, 19.vii.1936, R.H. Daggy, 1 female (pinned) (UMSP); Cass Co., 3-11.vii.1937, L.W. Orr, 1 male, 14 females (pinned) (UMSP); Clearwater Co., Iron Springs Bog Sci. Nat. Area, Sucker Creek, 47.253°N, 95.242°W, el 457 m, 13.viii.1988, M. Monson, 1 male (alcohol) (UMSP); same locality, 29.vi.1989, M. Monson, 1 female (alcohol) (UMSP); Clearwater Co., Itasca State Park, Mississippi River at County Road 117, 28.vi.1993, Holzenthal, 1 male, 1 female (pinned) (UMSP); Clearwater Co., Itasca State Park, 2.vii.1939, C.E. Nickel, 1 male (pinned) (UMSP); Cook Co., Temperance River, Sawbill Trail, 47°43.667'N, 90°52.834'W, 16.vii.2000, D.C. Houghton, 28 males, 40 females (alcohol) (UMSP); Cook Co., Superior National Forest, Temperance River at National Forest Campground, R.J. Blahnik, 1 male, 1 female (pinned) (UMSP); Crow Wing Co., Crow Wing Lake, State Road 371, 46°43.333'N, 94°21.500'W, 24.vi.2000, D.C. Houghton, 1 male (alcohol) (UMSP); Crow Wing Co., Little Pine River, Perry Lake Road, 46°37.000'N, 94°00.067'W, 20.viii.1999, D.C. Houghton, 25 males, 67 females (alcohol) (UMSP); Crow Wing Co., Pine River, County Road 11, 46°34.333'N, 94°02.000'W, 29.vi.2000, D.C. Houghton, 32 males, 3 females (pinned), 223 males, 120 females (alcohol) (UMSP) (NMNH); Douglas Co., Fairfield Creek, State Road 27, 45°52.333'N, 95°13.500'W, 23.vi.2000, D.C. Houghton, 1 male (alcohol) (UMSP); Douglas Co., Lake Carlos, Lake Carlos State Park, 45°59.500'N, 95°19.834'W, 23.vi.2000, D.C. Houghton, 3 males, 1 female (alcohol) (UMSP); Douglas Co., headwaters Long Prairie River, Lake Carlos State Park, 45°58.916'N, 95°19.67'W, 23.vi.2000, D.C. Houghton, 146 males & females (alcohol) (UMSP); Hubbard Co., Straight River, County Road 111, 48°52.333'N, 95°01.667'W, 03.vii.2000, D.C. Houghton, 142 males, 193 females (alcohol) (UMSP); Hubbard Co., Long Lake, County Road 06, public access, 46°51.500'N, 95°01.333'W, 03.vii.2000, D.C. Houghton, 1 female (alcohol) (UMSP); Hubbard Co., LaSalle Creek at County Road 40, 47°20.940'N, 95°09.900'W, el 425 m, 12.vi.1988, M. Monson, 17 males, 6 females (alcohol) (UMSP); same locality 25.vi.1988, M. Monson, 36 males 53 females (alcohol) (UMSP); same locality, 02.vii.1988, M. Monson, 31 males, 106 females (alcohol) (UMSP); same locality, 24.vii.1988, M. Monson, 127 males 67 females (alcohol) (UMSP); same locality, 26.vii.1988, M. Monson, 66 males 55 females (alcohol) (UMSP); same locality, 13.viii.1988, M. Monson, 113 males 42 females (alcohol) (UMSP); same locality 22.viii.1988, M. Monson, 17 males 34 females (alcohol) (UMSP); same locality, 10.ix.1988, M. Monson, 1 female (alcohol) (UMSP); same locality, 29.vi.1989, M. Monson, 162 males, 69 females (alcohol) (UMSP); same locality, 16.vii.1989, M. Monson, 108 males 97 females (alcohol) (UMSP); same locality, 27.vii.1989, M. Monson, 115 males, 35 females (alcohol) (UMSP); same locality, 13.viii.1989, M. Monson, 83 males, 49 females (alcohol) (UMSP); same locality 30.ix.1989, M. Monson, 1 male (alcohol) (UMSP); Itasca Co., Popple River, County Road 126, Chippewa National Forest, nr. Dora Lake, 47°43.546'N, 94°04.952'W, 10.vii.1999, D.C. Houghton, 1 male, 5 females (alcohol) (UMSP); Itasca Co., Bear Lake, headwaters Bear River, 47°40.634'N, 93°16.081'W, 14.vii.1999, D.C. Houghton, 2 females (alcohol) (UMSP); Itasca Co., Bear River, County Road 52, George Washington State Forest, 47°42.413'N, 93°15.424'W, 14.vii.1999, D.C. Houghton, 2 males, 3 females (pinned) (UMSP); Itasca Co., Deer Creek at County Road 230, 7 mi E Effie, 1 mi N Hwy. 1, 47°50.845'N, 93°29.333'W, 400 m, 29.vi.1996, Holzenthal et al., 26 males (pinned) (UMSP); Itasca Co., Big Fork River at Hwy. 1, 6.5 mi E Effie, 47°50.465'N, 93°30.156'W, 390 m, 28.vi.1996, Holzenthal et al., 10 males, 2 females (pinned) (UMSP); Itasca Co., outlet of Wabena Lake at County Road 49, ca. 11 mi N Grand Rapids, 411 m, 47.407°N, 93.485°W, R. Blahnik & F. Muñoz, 30 males, 3 females (pinned) (UMSP); Kittson Co., Caribou, 30.vi.1941, D.G. Denning, 1 male (alcohol) (UMSP); Koochiching Co., Black River, State Highway 11, 48°30.667'N, 93°48.000'W, 24.viii.2000, D.C. Houghton, 1 male, 2 females (alcohol) (UMSP); Koochiching Co., headwaters Black River, Fiero F.R., 48°25.888'N, 94°11.985'W, 11.vii.1999, D.C. Houghton, 2 males (alcohol) (UMSP); Koochiching Co., Black River, Indian Pines F.R., 48°27.986'N, 94°11.391'W, 11.vii.1999, D.C. Houghton, 2 males, 1 female (alcohol) (UMSP); Koochiching Co., Rainy River, confl. Little Fork River, State Highway 11, nr. International Falls, 48°31.174'N, 93°34.174'W, 12.vii.1999, D.C. Houghton, 64 males, 103 females (alcohol) (UMSP); Koochiching Co., Little Tamarac River, Lost River F.R., 48°04.634'N, 94°22.467'W, 13.vii.1999, D.C. Houghton, 3 males, 5 females (alcohol) (UMSP); Koochiching Co., Lost River, Lost River F.R., Pine Island State Forest, 48°05.320'N, 94°15.943'W, 13.vii.1999, D.C. Houghton, 1 male (alcohol) (UMSP); Koochiching Co., Big Fork River, State Highway 11, public access, Pine Island State Forest, nr. International Falls, 48°30.700'N, 93°42.754'W, el 244 m, 12.vii.1999, D.C. Houghton, 2 males (pinned) (UMSP); Lake Co., Gooseberry River, County Road 03, 47°10.000'N, 91°34.500'W, 11.vii.2001, D.C. Houghton, 87 males, 103 females (alcohol) (UMSP); Lake Co., W Branch Beaver River, County Road 3, 47°15.250'N, 91°22.834'W, 18.vii.1999, D.C. Houghton, 14 males, 2 females (alcohol) (UMSP); Lake Co., same data as holotype, 10 males (pinned) (UMSP); same locality, 30.vi.1991, R.J. Blahnik, 7 males (pinned) (UMSP); same locality, 11.viii.1991, Huisman & Holzenthal, 2 females (pinned) (UMSP); Lake of the Woods Co., Rapid River, County Road 98, 48°30.000'N, 94°39.500'W, 05.vii.2000, D.C. Houghton, 301 males 153 females (alcohol) (UMSP); Lake of the Woods Co., Zippel Bay State Park, 48°52.000'N, 94°50.000'W, 07.vii.2000, D.C. Houghton, 209 Males, 113 females (alcohol) (UMSP); Lake of the Woods Co., Moose Creek, County Road 01, 48°30.000'N, 94°36.000'W, 05.vii.2000, D.C. Houghton, 1 male 3 females (alcohol) (UMSP); Mahnomen Co., White Earth River, State Road 113, 47°10.667'N, 95°47.667'W, 04.vii.2000, D.C. Houghton, 583 males, 542 females (alcohol) (UMSP); Mahnomen Co., Little Elbow Creek, County Road 04, 47°12.000'N, 95°39.000'W, 04.vii.2000, D.C. Houghton, 3 males, 3 females (alcohol) (UMSP); Marshall Co., Middle River, County Road 04, 48°20.333'N, 96°49.000'W, 24.vii.2000, D.C. Houghton, 1 male (alcohol) UMSP); Mille Lacs Co., Lake Mille Lacs, Cove Bay, 46°06.667'N, 93°37.333'W, 28.vi.2000, D.C. Houghton, 1 male, 1 female (alcohol) (UMSP); Mille Lacs Co., Bradbury Bridge, County Road 103, 46°01.500'N, 93°41.500'W, 28.vi.2000, D.C. Houghton, 23 males, 30 females (alcohol) (UMSP); Mille Lacs Co., Rum River, Mille Lacs Kathio State Park, 46°07.000'N, 93°45.500'W, 28.vi.2000, D.C. Houghton, 287 males, 316 females (alcohol) (UMSP); Morrison Co., Platte River, County Road 35, 45°56.167'N, 94°14.917'W, 24.v.2000, D.C. Houghton, 81 males, 183 females (alcohol) (UMSP); Morrison Co., Pike Creek, Charles Lindberg State Park, 45°57.333'N, 94°23.500'W, 24.vi.2000, D.C. Houghton, 52 males, 27 females (alcohol) (UMSP); Morrison Co., Mississippi River, Charles Lindberg State Park, 45°57.000'N, 94°23.333'W, 24.vi.2000, D.C. Houghton, 1 male, 3 females (alcohol) (UMSP); Murray Co., Smith Lake, Lake Shetek State Park, 44°05.841'N, 95°41.473'W, 305 m, 01.viii.1999, D.C. Houghton, 1 female (pinned) (UMSP); Pennington Co., Thief River Falls, 20.viii.1941, D.G. Denning, 1 male, 20 females (alcohol) (UMSP); same locality, 18.vii.1941, D.G. Denning, 11 females (alcohol) (UMSP); Pine Co., Sand Creek, County Road 30, 46°07.667'N, 92°37.500'W, 28.vi.2001, D.C. Houghton, 35 males, 47 females (alcohol) (UMSP); Pine Co., Little Sand Creek, 1 mi E Cloverdale, 16.vii.1971, 6 males (alcohol) (UMSP); same locality, 8.viii.1971, 1 male (alcohol) (UMSP); same locality, 6.vii.1971, 1 male (alcohol) (UMSP); Pine Co., L. Pine Creek, State Road 18, 46°12.000'N, 92°59.500'W, 29.vi.2001, D.C. Houghton, 5 males, 17 females (alcohol) (UMSP); Pine Co., Big Pine Lake, State Road 18, 46°13.000'N, 93°01.500'N, 29.vi.2001, D.C. Houghton, 1 male, 1 female (alcohol) (UMSP); Pine Co., Kettle River, Banning State Park, 46°10.000'N, 92°50.000'W, 28.vi.2001, D.C. Houghton, 8 males, 4 females (alcohol) (UMSP); Pine Co. Kettle River, 9.viii.1991, L.J. Luedeman, 10 males, 11 females (alcohol) (UMSP); Polk Co., Red Lake River, US Route 75, 47°46.666'N, 96°38.000'W, 23.vii.2000, D.C. Houghton, 5 females (alcohol) (UMSP); Polk Co., Crookston, 7.vii.1935, D.G. Denning, 1 male, 2 females (pinned) (UMSP); same locality, 18.vii.1935, D.G. Denning, 1 female (pinned) (UMSP); same locality, 3.vii.1936, D.G. Denning, 1 female (pinned) (UMSP); same locality, 27.vii.1936, D.G. Denning, 1 female (pinned) (UMSP); same locality, 29.vi.1937, D.G. Denning, 1 male, 1 female (pinned) (UMSP); same locality, 4.vii.1937, D.G. Denning, 2 females (pinned) (UMSP); Ramsey Co., Lake Johanna, 6.vii.1937, D.G. Denning, 1 female (pinned) (UMSP); Ramsey Co., St. Paul, University golf course, A.A. Granovsky, 2 females (pinned) (UMSP); Ramsey Co., St. Paul, University Farm, 14.vii.1936, A.A. Granovsky, 1 female (pinned) (UMSP); Rock Co., un. spr., Mound Lakes, Blue Mound State Park, 43°43.000'N, 96°10.175'W, 07.vi.2000, D.C. Houghton, 1 male, 1 female (alcohol) (UMSP); Roseau Co., Roseau River, Winner F.R., 48°38.000'N, 95°29.000'W, 08.vii.2000, D.C. Houghton, 219 males, 167 females (alcohol) (UMSP); Roseau Co., Roseau River, Winner F.R., nr. Beltrami Island State Forest, 48°38.950'N, 95°28.967'W, 31.viii.1999, D.C. Houghton, 545 males, 858 females (alcohol) (UMSP); Roseau Co., Hayes Lake, Hayes Lake State Park, 48°38.083'N, 95°31.583'W, 08.vii.2000, D.C. Houghton, 3 males 3 females (alcohol) (UMSP); Roseau Co., Hansen Creek, Winner F.R., 48°38.000'N, 95°27.500'W, 08.vii.2000, D.C. Houghton, 4 males, 21 females (alcohol) (UMSP); Roseau Co., W Branch Warroad River, County Road 5, Beltrami Island State Forest, 48°44.867'N, 95°19.916'W, 31.viii.1999, D.C. Houghton, 1 female (alcohol) (UMSP); Saint Louis Co., Cloquet River, Bear Lake Road, 47°12.750'N, 91°56.500'W, 17.vii.2000, D.C. Houghton, 544 males, 513 females (alcohol) (UMSP); Saint Louis Co., Bear Lake, Cedar Bay campground, 47°12.500'N, 91°55.500'W, 17.vii.2000, D.C. Houghton, 3 males, 1 female (alcohol) (UMSP); Saint Louis County, Lake Cloquet River, Pequaywan Road, 47°06.833'N, 90°59.083'W, 17.vii.2000, D.C. Houghton, 146 males, 152 females (alcohol) (UMSP); Saint Louis Co., Smith Lake Spillway, Pequaywan Road, 47°08.000'N, 91°55.667'W, 17.vii.2000, D.C. Houghton, 41 males, 58 females (alcohol) (UMSP); St. Louis Co., Vermillion River, State Road 24, 48°07.400'N, 92°31.300'W, 20.vii.1999, D.C. Houghton, 1 male, 11 females (alcohol) (UMSP); St. Louis Co., Vermillion River, F.R. 491, 48°15.750'N, 92°33.500'W, 28.viii.2000, D.C. Houghton, 27 males, 21 females (alcohol) (UMSP); Saint Louis Co., Duck Creek, Echo Trail, 48°06.500'N, 92°03.667'W, 15.vii.2000, D.C. Houghton, 1 male (alcohol) (UMSP); St. Louis Co., St. Louis River, Jay Cooke State Park, 46°38.500'N, 92°21.000'W, 14.vii.2001, D.C. Houghton, 11 males, 32 females (alcohol) (UMSP); St. Louis Co., Coyote Creek, Pequaywan Road, 47°11.500'N, 91°52.000'W, 17.vii.2000, D.C. Houghton, 1 female (alcohol) (UMSP); St. Louis Co., Jenkins Creek, Town Line Road, 47°22.333'N, 91°57.333'W, 14.vii.2000, D.C. Houghton, 3 females (alcohol) (UMSP); St. Louis Co., Cadotte Lake, Cadotte Lake campground, 47°22.667'N, 91°54.917'W, 14.vii.2000, D.C. Houghton, 3 females (alcohol) (UMSP); Stearns Co., St. Cloud, 25.vii.1938, D.G. Denning, 3 males, 6 females (pinned) (UMSP); Todd Co., Birch Lake Creek, County Road 2, 46°47.666'N, 94°47.833'W, 16.vi.2000, D.C. Houghton, 1 male, 1 female (alcohol) (UMSP); Wadena Co., Shell River, Shell City Landing campground, 46°47.500'N, 94°56.500'W, 03.vii.2000, D.C. Houghton, 31 males, 34 females (alcohol) (UMSP); Yellow Medicine Co., Minnesota River, Mile 243.9, T115N, R38W, S28, 14.viii.1980, N. Potthoff, 2 males, 1 female (alcohol) (UMSP); **Mississippi:** Clark Allen Branch, Rt. 11, N Enterprise, 12.v.1988, C.M. & O.S. Flint, Jr., 2 males (pinned) (NMNH); Lauderdale, Chunky R., Dunn’s Falls Pk., 15 mi S Meridian, 13.v.1988, C.M. & O.S. Flint, Jr. 1 male (pinned) (NMNH).

#### Etymology.

We take pleasure in naming this species *Oecetis houghtoni* after Dr. Dave Houghton, Hillsdale College, Michigan, whose extensive survey work on the caddisflies of Minnesota provided the strongest evidence that this species is distinct from *Oecetis avara*.

#### Remarks.

While *Oecetis houghtoni* can be separated from *Oecetis avara* in the upper Midwest, USA, based on the characters listed, we were not always sure about specimens from other localities, due to the variation encountered. Once better understood, it is possible that this variation will be found to fall within the 2 species. However, it is also possible that additional undescribed species are represented. In particular, a form similar to *Oecetis houghtoni* in color and development of the forewing chord seems to be widespread in the Eastern United States and Canada, but differs in that the ventral lobes of the inferior appendages are very obtusely angled (Fig. 28A). This is possibly the form illustrated by Ross (1944) as *Oecetis avara*. We are not sure whether this form is referable to *Oecetis houghtoni* or represents yet another species. This form is discussed in more detail in the section on unassigned material following the species descriptions, but is not included among the paratype material. A somewhat similar form also occurs on the west coast of the United States.

### Oecetis maritza sp. n.

http://zoobank.org/F5F48488-90DB-485D-8714-274B18657F07

http://species-id.net/wiki/Oecetis_maritza

**Figure 15.**
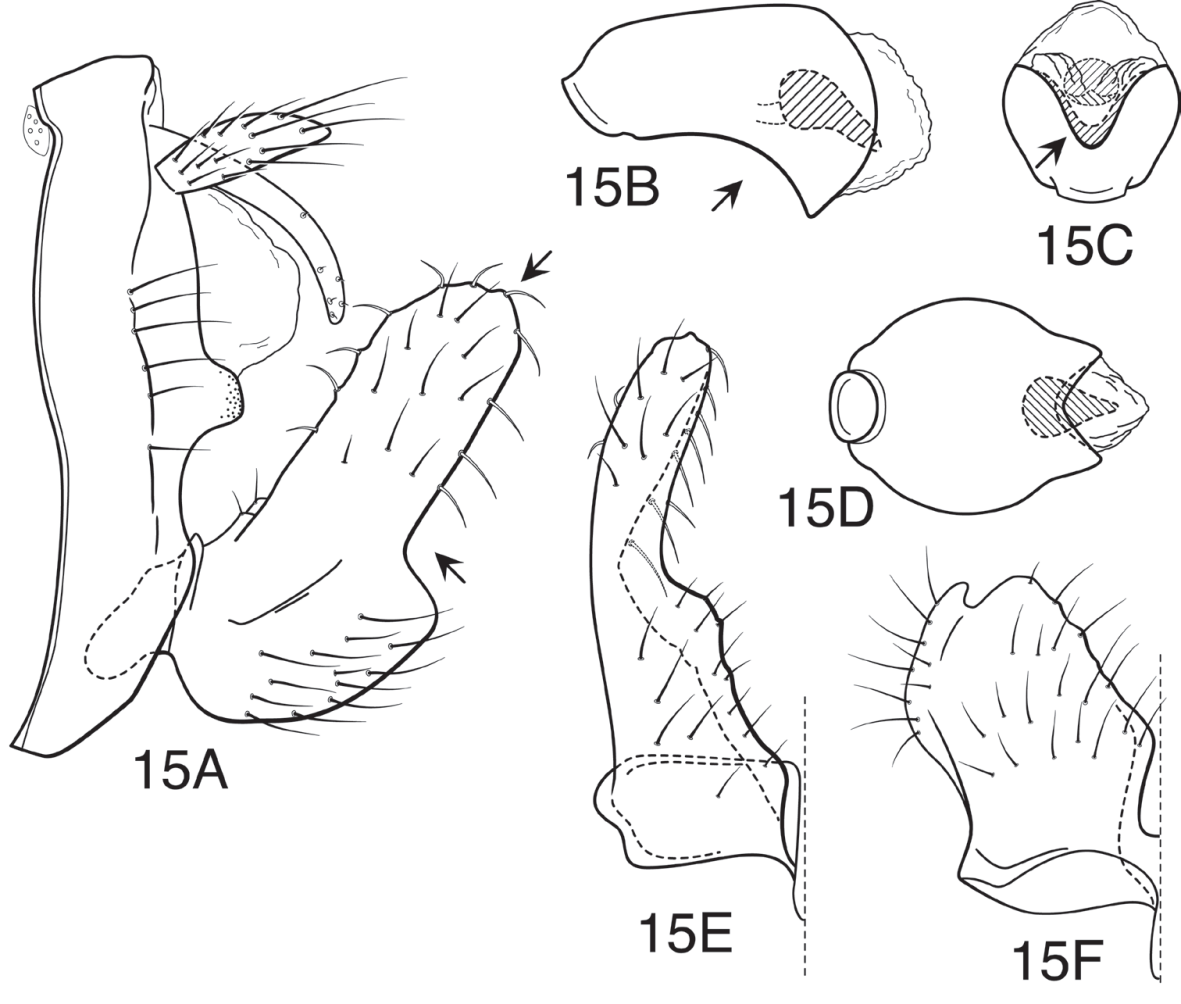
*Oecetis maritza* sp. n., male genitalia: **A** lateral **B** phallic apparatus, lateral **C** phallic apparatus, caudal **D** phallic apparatus, ventral **E** inferior appendage, caudal **F** inferior appendage, ventral.

Figs 15 , 53 , Map 3

#### Diagnosis.

*Oecetis maritza* is most similar to *Oecetis mexicana* sp. n., especially in the general form of the genitalia, with the ventral lobe obtusely angled (or forming a nearly right angle with the dorsal lobe), and in the general shape of the phallobase, with the ventral margin arched and apex U-shaped in caudal view. It is most easily distinguished by its smaller size and darker coloration (compare Figs 50 and 53). There are several minor genitalic differences. The dorsal lobe of the inferior appendage is relatively narrow throughout, and an asymmetrical phallic sclerite seems to be absent. In the shape of the dorsal lobe of the inferior appendage and absence of an asymmetrical phallic sclerite, *Oecetis maritza* resembles *Oecetis patula* sp. n. The latter differs in its larger size, light yellowish color, and especially in having the apex of the phallobase very broad in caudal view. Although *Oecetis maritza* is known from only 2 specimens (1 with the genitalia lost after being examined and illustrated), they are different enough from *Oecetis mexicana*, which also occurs in Costa Rica, to warrant their recognition as a distinct species.

#### Adult.

Forewing length: male (6.0 mm). Color yellowish-brown (darker than *Oecetis mexicana* sp. n.). Antennae whitish with indistinct, narrow annulations at intersection of antennomeres. Forewing spots small, brown, distinct, but not strongly contrasting with overall color of wing, spots at base discal and thyridial cells and base of fork V largest; veins of forewing chord very widely, almost evenly, spaced; chord pigmented; apical spots present, but not conspicuous. Setae on forewing membranes short, scant, decumbent, setae along veins in apical part of forewing semi-prostrate, laterally diverging. Fringe of setae along costal margin of forewing dense, very elongate, distinctly projecting.

#### Male genitalia.

Segment IX very short, with elongate setae along posterolateral margin. Tergum X with narrow, deflexed mesal lobe, lobe more or less uniform in width, narrowing apically, with small sensilla; lobe continuous basoventrally with projecting lateral membranous projections. Preanal appendage moderately elongate, length about 3 times maximum width, simple in structure, apical setae elongate. Inferior appendage with dorsal lobe narrow, uniform in width, rounded apically, ventral lobe angularly projecting; projection of ventral lobe forming obtuse angle with dorsal lobe; posterior margin of ventral lobe, as viewed ventrally (Fig. 15F), angularly bent near base, apices of paired lobes moderately diverging; basomesal projection of appendage very weakly developed, scarcely projecting, with several short, stiff setae; dorsal lobe with slightly thickened, mesally-curved setae on dorsal margin and stout, ventrally-curved setae on mesal surface. Phallobase relatively short; as viewed laterally, expanded apically, ventral margin distinctly arched, apex acute; as viewed ventrally, bulbously rounded in middle; ventral apex, as viewed caudally, U-shaped. Phallotremal sclerite prominent, tubular basally, ventral margin projecting, gradually narrowed, acute apically; asymmetrical lateral sclerite absent.

#### Holotype.

**Male** (pinned), **COSTA RICA: Guanacaste:** Parque Nacional Guanacaste, Estación Maritza, Río Tempisquito, 10°57.480'N, 85°29.820'W, 550 m, 13–15.vii.1992, F. Muñoz (UMSP) (INBIOCRI000513479).

#### Paratypes.

**COSTA RICA: Guanacaste:** Parque Nacional Guanacaste, Estación Patilla, Río Orosi, 10°59.460'N, 85°25.460'W, 700 m, 22-25.v.1990, Holzenthal & Blahnik, 1 male (pinned, missing genitalia) (UMSP).

#### Etymology.

This species is named *Oecetis maritza*, the name used as a noun in apposition, for Estación Maritza in Guanacaste National Park, where the type specimen was collected.

### Oecetis metlacensis

Bueno-Soria

http://species-id.net/wiki/Oecetis_metlacensis

**Figures 16–17.**
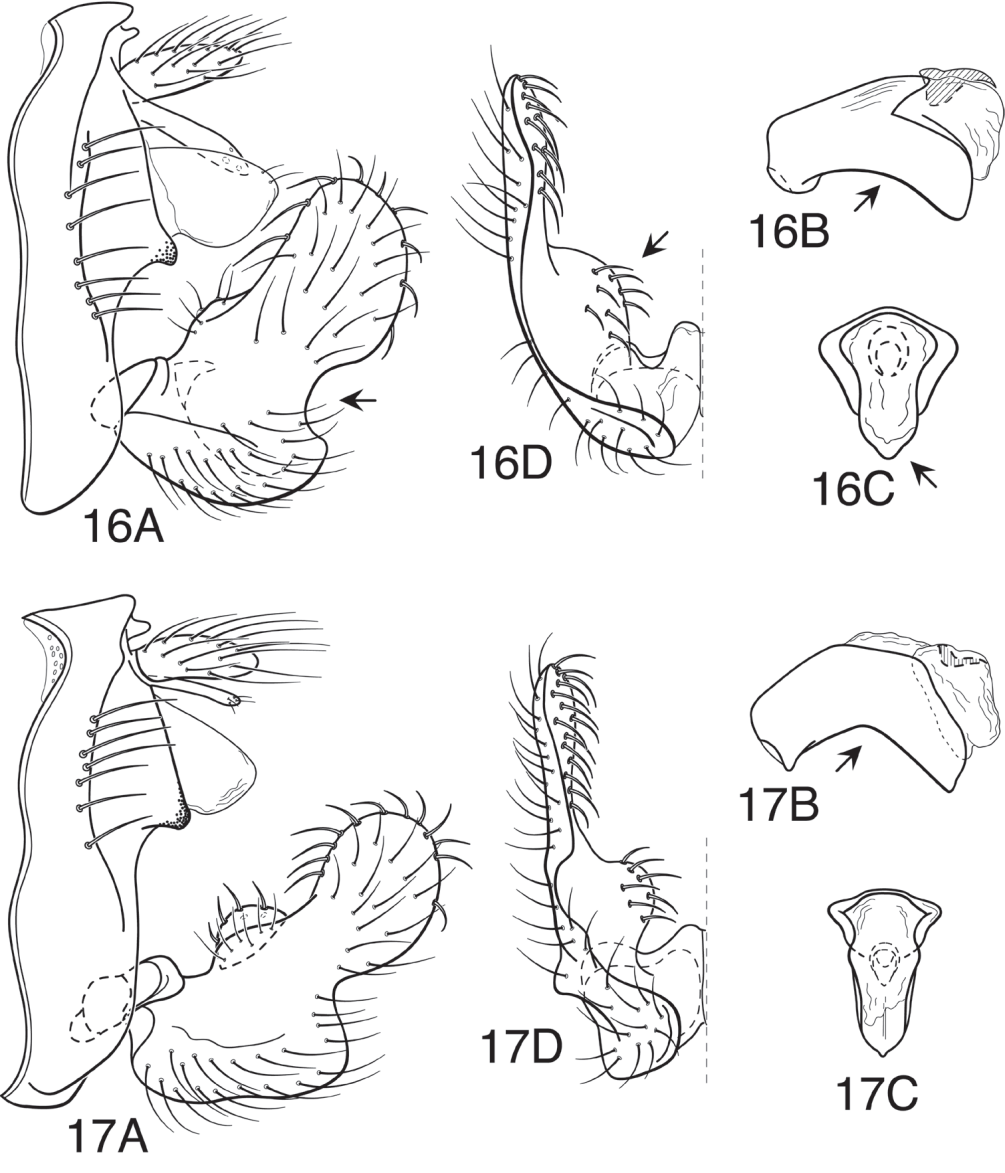
*Oecetis metlacensis* Bueno-Soria, male genitalia. **16** Paratype, Mexico: **A** lateral **B** phallic apparatus, lateral **C** phallic apparatus, caudal **D** inferior appendage, caudal **17** Specimen from Costa Rica: **A** lateral **B** phallic apparatus, lateral **C** phallic apparatus, caudal **D** inferior appendage, caudal.

Figs 16 , 17 , 36 , Map 5

Oecetis metlacensis Bueno-Soria 1981: 109, Figs 5, 6 [holotype male, Mexico, Veracruz, 5 km from Puente Villa Unión (IBUNAM)]; Houghton 2001: 91 [distribution – Arizona (specimens reassigned here to *Oecetis apache* sp. n.)].

#### Diagnosis.

*Oecetis metlacensis* is most readily diagnosed by its relatively large size (although this seems to be somewhat variable in specimens examined from Mexico), coloration (yellowish-brown with small forewing spots), shape of the inferior appendage, and by the V-shaped ventral apex of the phallobase, in caudal view. It is most similar to *Oecetis uncata* sp. n., *Oecetis apache* sp. n., and *Oecetis angulata* sp. n., all of which are in about the same size range and have the apex of the phallobase V-shaped in caudal view. *Oecetis uncata* has the ventral lobes of the inferior appendage more distinctly projecting and angled, and also has more distinct forewing spots, as well as a phallobase that is angled more apically. *Oecetis apache* differs in having the dorsal lobe of the inferior appendages more robust and the ventral lobe less defined, as well as in lacking any forewing spots at all. Its forewings are also somewhat wider. *Oecetis angularis* is lighter in color, has more distinct forewing spots, and has the phallobase less distinctly angled in lateral view. *Oecetis metlacensis* is also similar in size and general genitalic features to both *Oecetis disjuncta* and *Oecetis sordida* sp. n., but both of these species have the apex of the phallobase rounded or U-shaped, as viewed caudally.

#### Adult.

Forewing length: male (9.7–13.0 mm), female (6.9–10.0 mm). Color yellowish-brown. Antennae whitish with indistinct, narrow annulations at intersection of antennomeres. Forewing spots small, indistinct; spots at base of discal and thyridial cells and base of fork V largest, other spots very small; veins of forewing chord with *m* widely spaced, *s* and *r-m* veins relatively close; chord with very small spots at juncture of major veins; apical spots, at apices of major veins, not or scarcely evident. Forewings rather densely setose apically (causing apex to appear darker as viewed without magnification), setae along veins in apical part of forewing moderately elongate, semi-prostrate, laterally diverging. Fringe of setae along costal margin of forewing dense, short, not strongly projecting.

#### Male genitalia.

Segment IX very short, with elongate setae along posterolateral margin. Tergum X with narrow, deflexed mesal lobe, lobe short, very short in specimens from Costa Rica, tapering apically, apex with small sensilla; lobe continuous basoventrally with paired lateral membranous projections. Preanal appendage moderately elongate, length about 3 times maximum width, simple in structure, apical setae elongate. Inferior appendage with prominent, short rounded dorsal lobe and weakly to moderately projecting ventral lobe, separated from dorsal lobe by broadly rounded emargination; basomesal projection of appendage forming distinct rounded projection with short, stiff setae; dorsal lobe with stout, mesally-curved setae on dorsal margin, anteroventrally directed setae on anterior margin, and ventrally-curved setae on mesal surface. Phallobase moderately elongate and tubular basally, strongly, subangularly deflexed, more angularly in specimens from Costa Rica; ventral apex, as viewed caudally, V-shaped (distinctly keeled). Phallotremal sclerite prominent, basally forming relatively large tubular collar, ventral margin somewhat projecting, apex acute; asymmetrical lateral sclerite absent.

#### Material examined.

**MEXICO: Veracruz:** Río Tacolapan, Rt. 180, Km 551, 25-26.vii.1966, Flint & Ortiz, 2 males, 1 female (Paratypes, pinned), 1 male, 15 females (alcohol) (NMNH); Puente Tacolapan, E Tehada, 4.xii.1975, C.M. & O.S. Flint, Jr., 1 male (Paratype, pinned) (NMNH); Río Jamapa, 6 km N Coscomatepec, 2.v.1981, C.M. & O.S. Flint, Jr., 1 male (pinned) (NMNH); **COSTA RICA: Puntarenas:** Río Bellavista, ca. 1.5 km NW Las Alturas, 8°57.060'N, 82°50.760'W, el 1400 m, 18.ii.1986, Holzenthal, Morse, Fasth, 2 males, 3 females (alcohol) (UMSP); same location, 15–17.vi.1986, Holzenthal, Heyn, Armitage, 6 males (alcohol) (UMSP); same location, 8–9.iv.1987, Holzenthal, Hamilton, Heyn, 13 males, 11 females (alcohol) (UMSP); same location, 2–3.viii.1987, Holzenthal, Morse, Clausen, 7 males, 6 females (alcohol) (UMSP); same location, 10–11.viii.1990, Holzenthal, Blahnik, Muñoz, 16 males, 13 females (alcohol) (UMSP); same location, 16–17.iii.1991, Holzenthal, Muñoz, Huisman, 33 males, 24 females (alcohol) (UMSP); same location, 23.ix.1991, F. Muñoz & F. Quesada, 3 males (pinned) (UMSP); trib to Río Bellavista in Las Alturas (road to quarry), 8°57.120'N, 82°50.880'W, el 1480 m, 13–14,viii.1990, Holzenthal, Blahnik, Muñoz, 3 males (alcohol) (UMSP); Río Cotón in Las Alturas, 8°56.280'N, 82°49.560'W, el 1360 m, 18.ii.1991, Holzenthal, Muñoz, Huisman, 7 males, 6 females (alcohol) (UMSP); same location, 12.viii.1990, Holzenthal, Blahnik, Muñoz, 4 males, 5 females (alcohol) (UMSP); Zona Protectora Las Tablas, Río Cotón, Sitio Cotón, 8°56.460'N, 82°47.220'W, el 1460 m, 15.iv.1989, Holzenthal & Blahnik, 2 males, 6 females (alcohol) (UMSP); Río Guineal, ca 1 km (air) E Finca Helechales, 9°4.560'N, 83°5.520'W, el 840 m, 4.viii.1987, Holzenthal, Morse, Clausen, 29 males, 17 females (alcohol) (UMSP); Río Singri, ca. 2 km (air) S Finca Helechales, 9°3.420'N, 83°4.920'W, el 720 m, 21.ii.1986, Holzenthal, Morse, Fasth, 24 males, 30 females (alcohol) (UMSP). **San José:** Río Chirripó Pacífico, 9.5 km NE Rivas, 9°28.200'N, 83°35.460'W, el 1370 m, 23.ii.1986, Holzenthal, Morse, Fasth, 4 males, 2 females (alcohol) (UMSP).

#### Remarks.

Specimens of *Oecetis metlacensis* from Costa Rica are slightly different from those in Mexico. They agree in size and general color with the largest specimen examined from Mexico (Río Jamapa, Veracruz – Fig. 36). Paratype specimens examined from Mexico were distinctly smaller (but similar in coloration), accounting for the large size variance in the description of the species. Material examined from Mexico was limited, and thus it is difficult to assess the significance of the size variation observed in Mexican specimens. Compared to the paratype specimens from Mexico, the Costa Rican specimens have the apex of the phallobase slightly more elongate (Fig. 17B), the ventral lobe of the inferior appendage slightly more produced (Fig. 17A), the basomesal process of the inferior appendage more distinctly rounded (Figs 17A, 17D), and the mesal process of tergum X shorter (Fig. 17A). Mexican specimens were variable for most of these characters, especially in the development and angulation of the phallobase, without much evidence that this was anything more than intraspecific variation. A close side-by-side comparison reveals that specimens from Mexico and Costa Rica are very similar, especially when the variation among Mexican specimens is taken into account. In the absence of specimens from the intervening area between Mexico and Costa Rica and evidence to the contrary, this variation is here considered intraspecific.

### Oecetis mexicana sp. n.

http://zoobank.org/7C017C27-4505-403D-BE3F-2AA94E5E0120

http://species-id.net/wiki/Oecetis_mexicana

**Figures 18–20.**
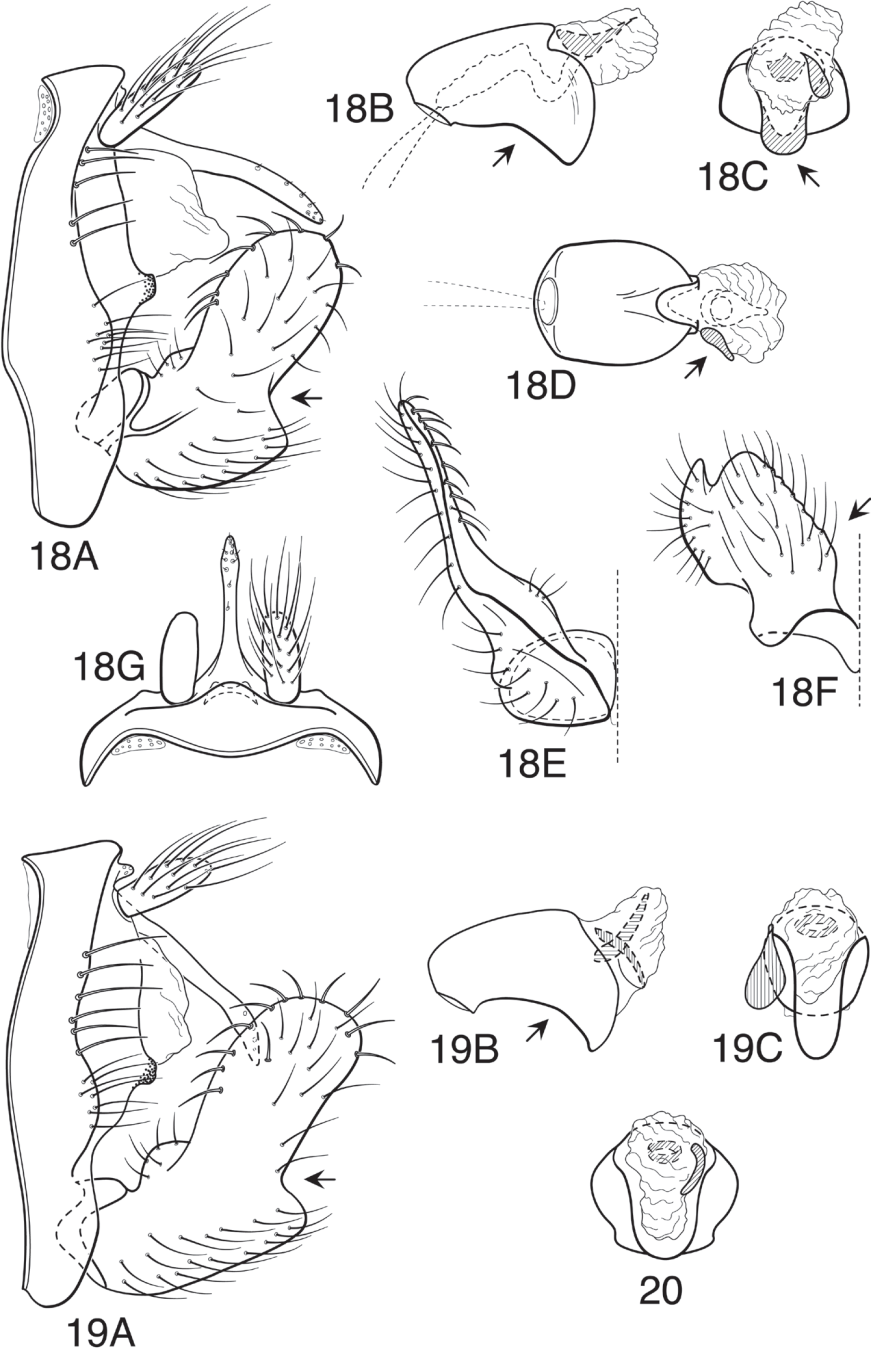
*Oecetis mexicana* sp. n., male genitalia **18** Specimen from Chiapas, Mexico: **A** lateral **B** phallic apparatus, lateral **C** phallic apparatus, caudal **D** phallic apparatus, ventral **E** inferior appendage, caudal **F** inferior appendage, ventral **G** segment IX and tergum X, dorsal **19** Specimen from Darien, Panama: **A** lateral **B** phallic apparatus, lateral **C** phallic apparatus, caudal **20** Specimen from Canal Zone, Panama: phallic apparatus, caudal.

Figs 18 , 19 , 20 , 50 , Map 4

#### Diagnosis.

This is one of the most widespread species in the Neotropical material formerly included within *Oecetis avara*. Although common in Mexico it also extends into Panama and even to Ecuador, but seems to be somewhat less common in the southern part of its distribution, where it is often replaced by other species in the *Oecetis avara* group. Taken as a whole, the species is more variable than most of the species of the *Oecetis avara* group from Central and South America. *Oecetis mexicana* has the same generalized genitalic structures as *Oecetis avara* and *Oecetis houghtoni* sp. n. including a short phallobase, arched ventrally and with a U-shaped ventral apex (as viewed caudally) and an asymmetric apical phallic sclerite. It differs primarily in its smaller size, yellow coloration and prominent, rounded forewing spots, and by the obtuse to right angled ventral lobe of the inferior appendages (Figs 18A and 19A). In general form of the genitalia, it comes close to some specimens from eastern USA, currently unassigned to species (see discussion of unassigned material at end of species descriptions), which also have inferior appendages with the ventral margin obtusely angled (Fig. 28A). These, however, are much darker in color and have only indistinct forewing spots. The most similar species to *Oecetis mexicana*, within the zone of sympatry, are *Oecetis constricta* and *Oecetis campana*, which also have inferior appendages that are obtusely angled and are similar in size. *Oecetis constricta* is separated by having the apex of the phallobase distinctively “pinched,” as viewed caudally and usually has the phallobase more angularly bent, as well as being slightly smaller in size. In one population assigned to *Oecetis constricta* from Carara in Costa Rica (but not included in the paratype series), specimens lacked the “pinched” apex of that species and thus were similar to *Oecetis mexicana*. These specimens were discussed in the diagnosis for *Oecetis constricta*. *Oecetis mexicana* also closely resembles *Oecetis campana* sp. n., which differs primarily in that the ventral margin of the phallobase is nearly straight rather than arched or angled (see the diagnosis for *Oecetis campana* for other minor differences).

#### Adult.

Forewing length: male (6.5–8.0 mm), female (5.8–7.5 mm). Color yellowish, palps very slightly darker. Antennae pale yellow with indistinct, narrow annulations at intersection of antennomeres. Forewing spots distinct and large; spots at base of discal and thyridial cells largest, spot at base of fork V nearly as large; veins of forewing chord widely and usually nearly evenly spaced, with crossveins nearly perpendicular, or *s* and *r-m* veins slightly closer; chord with small spots at juncture of major veins, those at opposite ends of chord larger; apical spots, at apices of major veins prominent, pigmentation extending beyond veins. Setae along veins in apical part of forewing elongate, semi-prostrate, laterally diverging. Fringe of setae along costal margin of forewing moderately dense, elongate, suberect.

#### Male genitalia.

Segment IX very short, with elongate setae along posterolateral margin. Tergum X with narrow, deflexed mesal lobe, lobe relatively elongate, uniform in width or somewhat lobulate apically, apex with small sensilla; lobe continuous basoventrally with short paired lateral membranous projections. Preanal appendage moderately elongate, length about 3 times maximum width, simple in structure, apical setae elongate. Inferior appendage with prominent rounded dorsal lobe and angularly projecting ventral lobe; ventral lobe moderately projecting, forming approximately right to distinctly obtuse angle with dorsal lobe, posterior margin of ventral lobe, as viewed ventrally (Fig. 18F), with distinctly angular bend near base; basomesal projection very weakly developed, scarcely or not protruding, with short, stiff setae; dorsal lobe with stout, mesally-curved setae on dorsal margin and stout, ventrally-curved setae on mesal surface. Phallobase, as viewed laterally, short and tubular, distinctly curved or “arched” ventrally (not angular); apex, as viewed caudally, with ventral margin U-shaped, not keeled ventrally (Fig. 18C), rarely narrowly U-shaped (Fig. 19C), apex not ridged or “burred.” Phallotremal sclerite prominent, basally forming short tubular collar, ventral margin projecting, apex acute; asymmetrical lateral sclerite present, usually distinctly protruding, located on right side, or sometimes on left.

#### Holotype.

**Male** (pinned), **MEXICO: Chiapas:** Río Tulija, 48 km S Palenque, 17.v.1981, C.M. & O.S. Flint, Jr. (NMNH) (UMSP000208191).

#### Paratypes.

**MEXICO: Chiapas:** same data as holotype, 8 males, 4 females (pinned), 3 males, 1 female (alcohol) (NMNH); Río Contento, 7 km N Ocosingo, 20.v.1981, C.M. & O.S. Flint, Jr., 6 males, 6 females (pinned), 13 males, 4 females (alcohol) (NMNH); Cascada Misolja, 20 km S Palenque, 17-18.v.1981, C.M. & O.S. Flint, Jr., 1 male (pinned) (NMNH); Río Lacanja, 22 km N Ocosingo, 19.v.1981, C.M. & O.S. Flint, Jr., 3 males, 2 females (pinned) (NMNH); **Oaxaca:** Río Valle Nacional, Chiltepec, 25.v.1981, C.M. & O.S. Flint, Jr., 6 males, 6 females (alcohol) (NMNH); Ao. Choapan, Bethania, 31 km S Tuxtepec, 24.v.1981, C.M. & O.S. Flint, Jr., 5 males, 4 females (pinned) (NMNH); **San Luis Potosí:** Palitla, 5.vi.1966, O.S. Flint, 2 males, 7 females (alcohol) (NMNH); 25 mi N Tamazunchale, el 400 m, 3–4.viii.1963, Duckworth & Davis, 1 male (alcohol) (NMNH); Huichhuayan, 4.vi.1967, O.S. Flint, Jr., 2 males (pinned) (NMNH); **Tamaulipas:** Río Corona, 18 mi N Ciudad Victoria, 13.iii.1982, J.E. Gillaspy, 1 male, 1 female (alcohol) (NMNH); **Veracruz:** nr. El Encero, Rt. 140, km 347, 22.vii.1965, Flint & Ortiz, 1 male (pinned) (NMNH); **BELIZE: Cayo:** Río Privassión, 14.vii.1973, Y. Sedman, 1 male, 1 female (alcohol) (NMNH); **HONDURAS:** Río Humuya, NW Comayagua, 3.viii.1967, O. S. Flint, Jr., 1 male (pinned) (NMNH); **Olancho:** Río Agua Amarilla, Pacayal, 5 km S El Carbon, 27.vii.1989, O.D. L. Lentz & Lopez, 1 male (alcohol) (NMNH); **NICARAGUA: Zelaya:** Cerro Saslaya, 13°44'N, 85°01'W, el 700 m, --iv.1996, J. M. Maes & J. Hernandez, 3 males, 15 females (alcohol) (NMNH); **COSTA RICA: Alajuela:** Río Pizote, ca. 5 km N Dos Ríos, 10°56.880'N, 85°17.460'W, el 470 m, 9.iii.1986, Holzenthal & Fasth, 33 males, 9 females (alcohol) (UMSP); Río Pizote, ca. 5 km (air) S Brasilia, 10°58.320'N, 85°20.700'W, el 390 m, 12.iii.1986, Holzenthal & Fasth, 8 males, 15 females (alcohol) (UMSP); **Guanacaste:** Parque Nacional Guanacaste, El Hacha, Quebrada Pedregal, 10°58.980'N, 85°32.340'W, el 300 m, 27.vii.1987, Holzenthal, Morse, Clausen, 1 male (pinned) (UMSP); Río Aguacate, 0.5 km E Aguacate, 10°33.900'N, 84°56.340'W, el 590 m, 16.ii.1992, Holzenthal, Muñoz, Kjer, 2 males, 2 females (pinned) (UMSP); Río Mena, 4.2 km W Santa Cecilia, 11°3.540'N, 85°26.880'W, el 260 m, 11.iii.1986, Holzenthal & Fasth, 4 males (alcohol) (UMSP); **Heredia:** Quebrada Ceiba, 6 km E Cháves, 10°22.920'N, 83°55.320'W, el 50 m, 2.vii.1992, F. Muñoz, 3 males (pinned) (INBIO); Río Sarapiquí, 300 m S Chilamate, 10°26.160'N, 84°3.600'W, el 40 m, 30.vi.1991, F. Muñoz, 3 males, 2 females (pinned) (INBIO); Río Bijagual, 3.5 km S Chilamate, 10.436°N, 84.060°W, el 40 m, 1.vii.1992, F. Muñoz, 3 males (pinned) (INBIO); Est. Biol. La Selva, Quebrada Sura, 10°26.220'N, 84°0.600'W, el 50 m, 20–21.vi.1986, Holzenthal, Heyn, Armitage, 3 males (alcohol) (UMSP); **Limón:** E.A.R.T.H., Río Dos Novillos, 10°13.200'N, 83°35.460'W, el 20 m, 3.ii.1992, Holzenthal, Muñoz, Kjer, 12 males, 7 females (pinned) (UMSP); same location, 26.vi.1992, Contreras & Muñoz, 3 males (pinned) (UMSP); E.A.R.T.H., Río Parismina, 10°14.880'N, 83°34.200'W, el 5 m, 4.ii.1992, Holzenthal, Muñoz, Kjer, 19 males, 2 females (pinned) (UMSP); E.A.R.T.H., Río Destierra, Pozo Azul, 10°12.480'N, 83°34.440'W, el 15 m, 5.ii.1992, Holzenthal, Muñoz, Kjer, 20 males, 5 females (pinned) (UMSP) (INBIO); same location, 27.vi.1992, F. Muñoz, 5 males (pinned) (INBIO); **Puntarenas:** Río Singri, ca 2 km (air) S Finca Helechales, 9°3.420'N, 83°4.920'W, el 720 m, 21.ii.1986, Holzenthal, Morse, Fasth, 1 male (alcohol) (UMSP); **PANAMA: Canal Zone:** Pipeline Rd., --vii.1967, W. Wirth, 1 male, 1 female (pinned) (NMNH); **Darien:** Río Tuira, between El Real & Río Pucuro, 16.ii.1985, J. Louton, 2 males (pinned), 1 male, 12 females (alcohol) (NMNH); Río Tuira at Río Pacuro, 16–17.ii.1985, J. Louton, 5 males, 2 females (pinned) (NMNH); Río Tuira, at Boca de Cupe, 18.ii.1985, J. Louton, 1 male (alcohol) (NMNH); **San Blas:** Río Carti Grande, nr. coast, 2.iii.1985, Flint & Louton, 12 males (pinned), 24 males, 28 females (alcohol) (NMNH); **ECUADOR: Los Ríos:** Quevedo, 11.v.1975, Spangler, Gurney, Langley & Cohen, 2 males, 8 females (alcohol) (NMNH); Quevedo, 56 km N, Río Palenque Biol. Sta., el 250 m, 18–29.vii.1976, J. Cohen, 7 males, 1 female (alcohol) (NMNH); **VENEZUELA: Lara:** Parque Nactional Terepaima, Quebrada San Antonio, 9°51.754'N, 69°13.098'W, el 631 m, 17.vi.2001, Holzenthal, Blahnik, Paprocki, Cressa, 1 male (pinned) (UMSP).

#### Etymology.

This species is named *Oecetis mexicana* for Mexico, the country of origin of the holotype specimen, where this species seems to be especially abundant and widespread.

### Oecetis patula sp. n.

http://zoobank.org/96E8DC7F-AC78-40D6-A184-8D7D98BDED8F

http://species-id.net/wiki/Oecetis_patula

**Figure 21.**
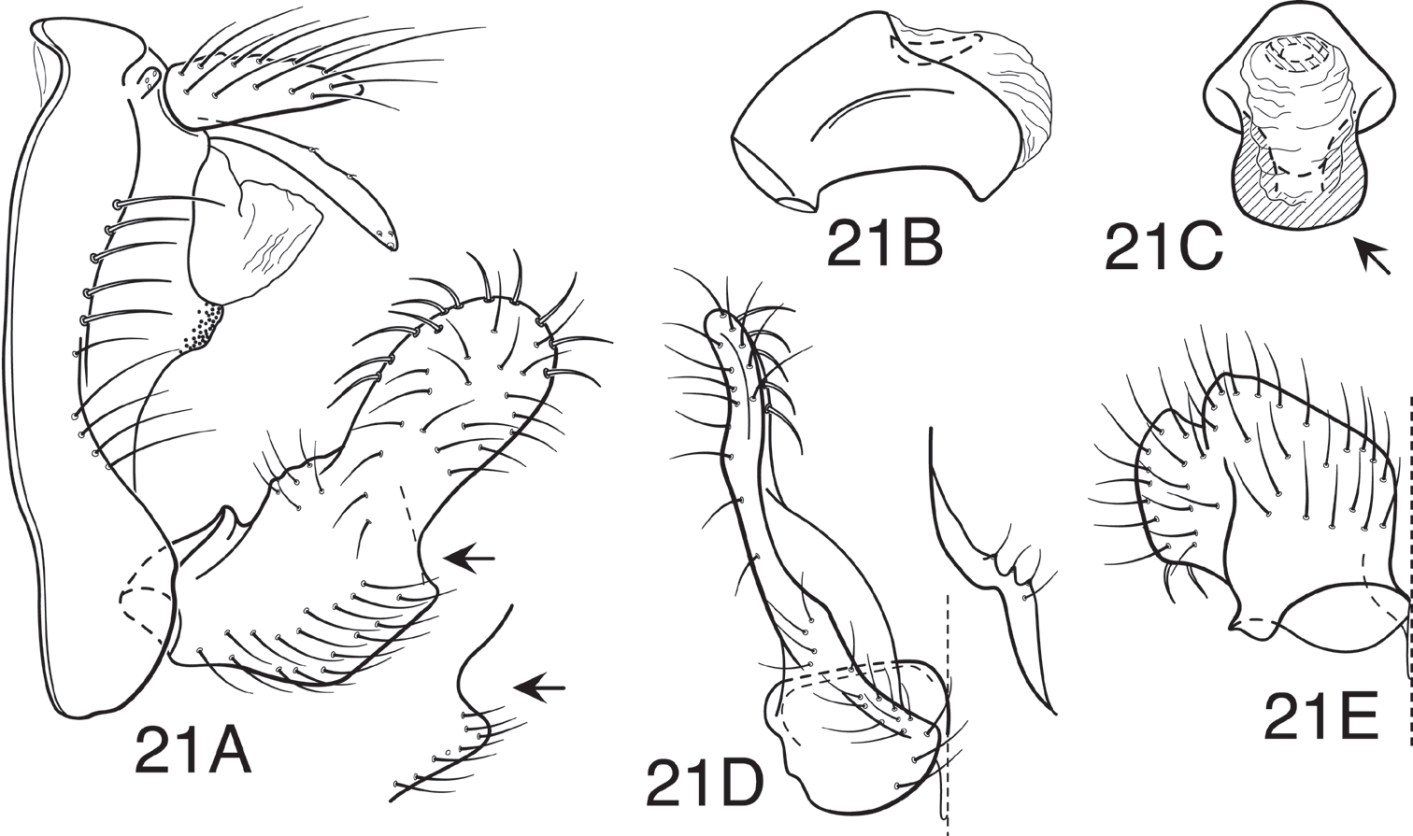
*Oecetis patula* sp. n., male genitalia: **A** lateral; inset, variation in inferior appendage **B** phallic apparatus, lateral **C** phallic apparatus, caudal **D** inferior appendage, caudal; inset, variation in development of basomesal process **E** inferior appendage, ventral.

Figs 21 , 49 , Map 3

#### Diagnosis.

The most distinctive diagnostic character of *Oecetis patula* is the shape of the apex of the phallobase. In this species the apex of the phallobase is distinctly downturned and has its lateral margins explanate, causing it to appear wide in caudal view (more broadly U-shaped than *Oecetis mexicana* sp. n.). In the general shape of the inferior appendage, with the dorsal lobe relatively narrow and the basomesal process very weakly developed, it perhaps compares most closely to *Oecetis maritza* sp. n. Both species seem to lack an asymmetrical phallic sclerite, which is always present in *Oecetis mexicana*. *Oecetis maritza* differs in its smaller size, darker overall coloration, and in having a phallobase that is more narrowly U-shaped in caudal view. In general features *Oecetis patula* compares most closely to, and is most likely to be confused with, *Oecetis mexicana*, which is similar in size and general coloration, but has the apex of the phallobase more narrowly U-shaped in caudal view and always possesses an asymmetrical phallic sclerite. Also, in most of the specimens of *Oecetis patula* examined, the forewings spots were distinctly smaller than in *Oecetis mexicana*. However, in a couple of the specimens from Nicaragua the spots were relatively large, but the spots were then irregular in shape, V-shaped rather than distinctly rounded.

#### Adult.

Forewing length: male (8.1–8.5 mm), female (6.5 mm). Color pale yellowish, (slightly paler than *Oecetis mexicana*). Antennae pale yellow with indistinct, narrow annulations at intersection of antennomeres. Forewing spots small, but distinct (smaller than those of *Oecetis mexicana*); spots at base of discal and thyridial cells and base of fork V largest, sometimes forming deltoid spots in angle formed by fork; veins of forewing chord widely and usually nearly evenly spaced, with crossveins nearly perpendicular, or *s* and *r-m* veins slightly closer; chord with spots at juncture of major veins, those at opposite ends of chord larger, or chord itself sometimes pigmented; apical spots, at apices of major veins either small or large, with pigmentation extending beyond veins. Setae along veins in apical part of forewing elongate, semi-prostrate, laterally diverging. Fringe of setae along costal margin of forewing moderately dense, suberect.

#### Male genitalia.

Segment IX very short, with elongate setae along posterolateral margin. Tergum X with narrow, deflexed mesal lobe, lobe moderately elongate, uniform in width or somewhat lobulate apically, apex with small sensilla; lobe continuous basoventrally with short, paired lateral membranous projections. Preanal appendage moderately elongate, length about 3 times maximum width, simple in structure, apical setae elongate. Inferior appendage with relatively narrow, apically rounded dorsal lobe and angularly projecting ventral lobe; ventral lobe very weakly projecting, forming approximately right (Fig. 21A inset) to distinctly obtuse angle (Fig. 21A) with dorsal lobe, posterior margin of ventral lobe, as viewed ventrally (Fig. 21E), with distinctly angular bend near base; basomesal projection very weakly developed, scarcely (Fig. 21D inset) or not protruding (Fig. 21D), with short, stiff setae; dorsal lobe with stout, mesally-curved setae on dorsal margin and stout, ventrally-curved setae on mesal surface. Phallobase, as viewed laterally, short and tubular, distinctly curved or “arched” ventrally; apex, as viewed caudally, with ventral margin very broadly U-shaped, not keeled ventrally (Fig. 21C), apex not ridged or “burred.” Phallotremal sclerite relatively prominent, basally forming short tubular collar, ventral margin projecting, apex acute; asymmetrical lateral sclerite apparently absent.

#### Holotype.

**Male** (pinned), **GUATEMALA: Baja Verapaz:** Rt 5, Km 156, Puente Las Burras, 22–24.vi.1966, Flint & Ortiz (NMNH) (UMSP000208338).

#### Paratypes.

**NICARAGUA: Jinotega:** Cerro Kilambé, stream nr. camp, 13°35.216'N, 85°42.722'W, el 1200 m, 13.vii.2001, Chamorro, Maes, Hernandez, Sanger, 5 males, 1 female (pinned) (UMSP).

#### Etymology.

This species is named *Oecetis patula* from the Latin word *patulus*, meaning open or spread out, and referring to the wide ventral apex of the phallobase of the species.

### Oecetis protrusa sp. n.

http://zoobank.org/DC821434-D45B-4B66-B535-98747DF2BB7D

http://species-id.net/wiki/Oecetis_protrusa

**Figure 22.**
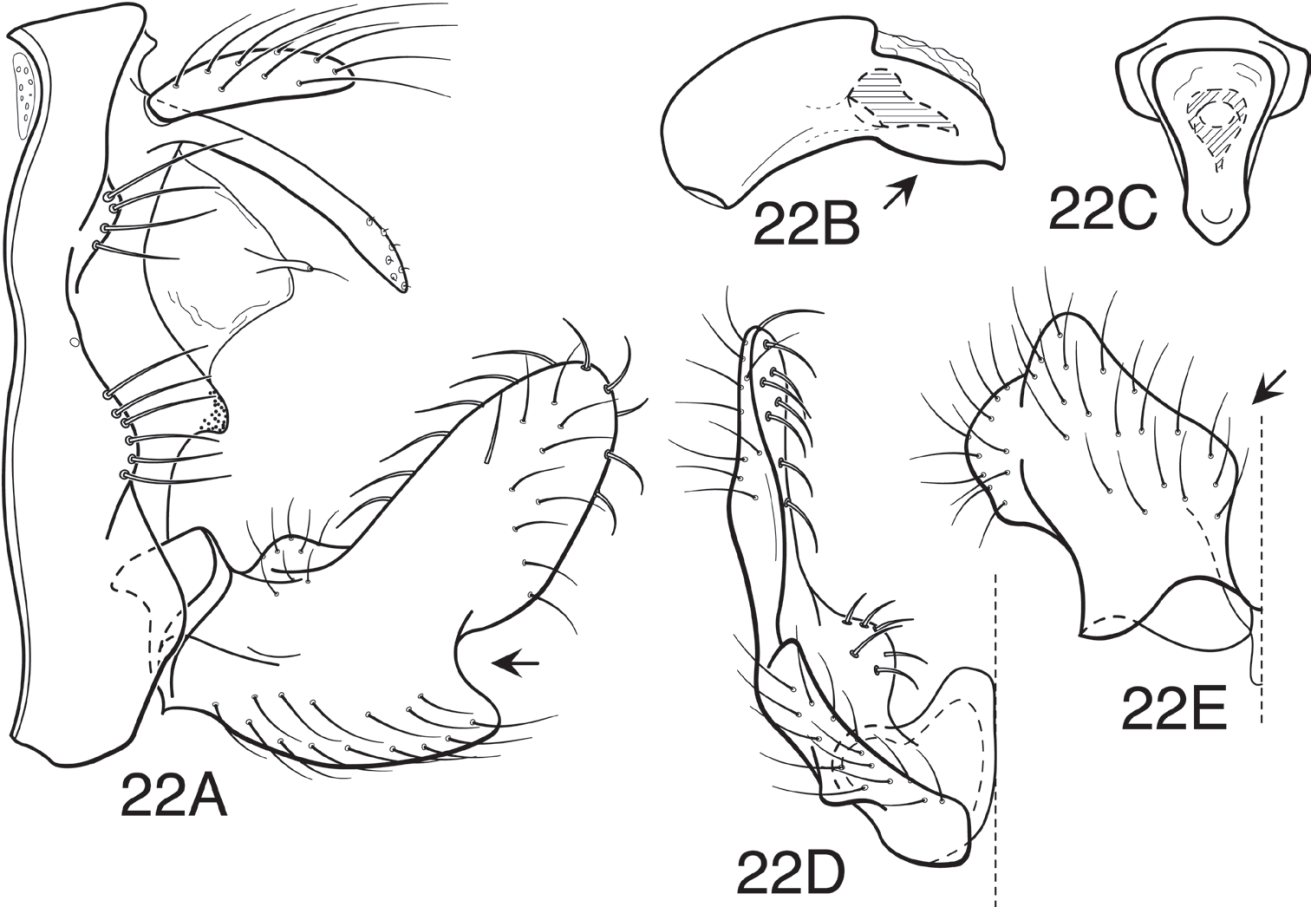
*Oecetis protrusa* sp. n., male genitalia: **A** lateral **B** phallic apparatus, lateral **C** phallic apparatus, caudal **D** inferior appendage, caudal **E** inferior appendage, ventral.

Figs 22 , 55 , Map 1

#### Diagnosis.

*Oecetis protrusa* sp. n. is most reliably diagnosed by its size, yellowish color, large, conspicuous forewing spots (often with those at the base of the discal and thyridial cells larger than those in Fig. 55), and particularly by the shape of its phallobase, which has its apex protruding and its ventral margin somewhat convexly bulged preapically, often with the extreme apex slightly recurrent. This is a subtle character, but evident once it is recognized. Additionally, the inferior appendages have the ventral lobe prominent and acutely angled with respect to the dorsal lobe. *Oecetis protrusa* is perhaps most likely to be confused with *Oecetis verrucula* sp. n., or *Oecetis mexicana* sp. n., which are similar in size and frequently co-occur with *Oecetis protrusa*. Like *Oecetis verrucula*, the ventral lobes of the inferior appendages are acutely angled, but unlike *Oecetis verrucula*, these are very distinctly, angularly diverging basally, as viewed ventrally (compare Figs 22E and 26E). *Oecetis verrucula* additionally differs by having a differently shaped, shorter phallobase with a small ventral “wart,” and is also slightly darker in color, with smaller, usually somewhat oval, forewing spots. *Oecetis mexicana* is very similar in color, particularly in having large, rounded forewing spots, but differs in that the ventral lobe of the inferior appendages is either obtusely angled or nearly at a right angle and is less divergently bent at its base, as well as in having a phallobase that is merely arched ventrally, without a convex bulge. In sorted, sympatric series, it is evident (on comparison) that *Oecetis mexicana* is very slightly darker in color and larger in size than *Oecetis protrusa*, but the differences are admittedly subtle. In a large series from Guácimo, Limón, Costa Rica (Escuela de Agricultura de la Región Tropical Húmeda), where *Oecetis protrusa* and *Oecetis mexicana* occurred sympatrically and were about equally abundant, the length of the phallobase in *Oecetis protrusa* was somewhat shorter than usual and the convex ventral bulge less distinctively developed. Cleared specimens, however, were easily sorted and the inferior appendages were typical of the 2 species. On account of the slightly shorter phallic length of these specimens, and preapical bulge on the phallobase, they could possibly be confused with *Oecetis tumida* sp. n. However, the inferior appendage of that species is quite different in form, with the ventral lobe much shorter and less angularly projecting.

#### Adult.

Forewing length: male (7.0–8.0 mm), female (6.0–6.5 mm). Color pale yellowish (paler than *Oecetis mexicana*). Antennae pale yellow with indistinct, narrow annulations at intersection of antennomeres. Forewing spots very large and distinct; spots at base of discal and thyridial cells largest, that of discal cell nearly round, spot at base of fork V nearly as large; veins of forewing chord widely and usually nearly evenly spaced, with crossveins nearly perpendicular, or *s* and *r-m* veins slightly closer; chord with distinct spots at juncture of major veins, those at opposite ends of chord larger; apical spots, at apices of major veins prominent, pigmentation extending beyond veins. Setae along veins in apical part of forewing elongate, semi-prostrate, laterally diverging. Fringe of setae along costal margin of forewing moderately dense, suberect.

#### Male genitalia.

Segment IX very short, with elongate setae along posterolateral margin. Tergum X with narrow, deflexed mesal lobe, lobe relatively elongate, uniform in width or somewhat lobulate apically, apex with small sensilla; lobe continuous basoventrally with short, paired lateral membranous projections. Preanal appendage moderately elongate, length about 3 times maximum width, simple in structure, apical setae elongate. Inferior appendage with prominent dorsal lobe, usually somewhat narrowed and rounded apically, and angularly projecting ventral lobe; ventral lobe distinctly projecting, forming acute angle with dorsal lobe; mesal margin of ventral lobe, as viewed ventrally (Fig. 22E), with distinctly angular bend near base; basomesal projection moderately developed, forming small rounded projection with short, stiff setae; dorsal lobe with stout, mesally-curved setae on dorsal margin and stout, ventrally-curved setae on mesal surface. Phallobase, as viewed laterally, moderately elongate, ventral margin distinctly protruding, with noticeable preapical convex bulge, apex usually slightly ridged or burred; as viewed caudally, with apex narrowly U-shaped, not keeled ventrally. Phallotremal sclerite prominent, basally forming short tubular collar, ventral margin projecting, apex acute; asymmetrical lateral sclerite probably always present, but only slightly protruding and not very evident, located on right side.

#### Holotype.

**Male** (pinned), **COSTA RICA: Heredia:** Parque Nacional Braulio Carrillo, Est. Magsasay, Río Peje, 10°24.120'N, 84°3.000'W, el 130 m, 25–26.viii.1990, Holzenthal, Blahnik, Huisman (UMSP) (UMSP000207778).

#### Paratypes.

**MEXICO: Chiapas:** 7.8 mi E Pichucalco, 27–28.vii.1966, Flint & Ortiz, 4 males, 3 females (pinned) (NMNH); same location, 7.xii.1975, C.M. & O.S. Flint, Jr., 6 males (pinned) (NMNH); Río Lacanja, 22 km N Ocosingo, 19.v.1981, C.M. & O.S. Flint, Jr., 2 males (pinned) (NMNH); Cascada Misolja, 20 km S Palenque, 17–18.v.1981, C.M. & O.S. Flint, Jr., 5 males, 2 females (pinned), 8 males, 34 females (alcohol) (NMNH); Río Tulija, 48 km S Palenque, 17.v.1981, C.M. & O.S. Flint, Jr., 2 males (pinned), 5 males, 2 females (alcohol) (NMNH); **Tabasco:** Río Puyacatengo, E Teapa, 28–29.vii.1966, Flint & Ortiz, 15 males, 6 females (alcohol) (NMNH); **Veracruz:** Los Tuxtlas area, “Los Tuxtlas” Biological Station, 31 km NE Catemaco, Río Palma, above LaPalma, 7–14.v.1981, C.M. & O.S. Flint, Jr., 3 males, 3 females (pinned) (NMNH); Los Tuxtlas area, “Los Tuxtlas” Biological Station, 31 km NE Catemaco, Río Palma, below LaPalma, 5.v.1981, C.M. & O.S. Flint, Jr., 4 males (pinned), 4 males, 12 females (alcohol) (NMNH); Río Tacolapan, Rt. 180, km 551, 25–26.vii.1966, Flint & Ortiz, 2 males (alcohol) (NMNH); **GUATEMALA: Alta Verapaz**, Río Cahabon, Chibite, 3.i.1989, B. C. Kondratieff, 7 males (alcohol) (NMNH); **NICARAGUA:** Puente Quinama, E Villa Somoza, 29.vii.1967, O. S. Flint, Jr., 1 male (pinned) (NMNH); **COSTA RICA:** Las Canas, Río Cordobicí, 26.vii.1967, O. S. Flint, Jr., 2 males (pinned), 1 male, 1 female (alcohol) (NMNH); Las Canas, 13.vii.1965, P. J. Spangler, 7 males (alcohol) (NMNH); 10 mi NW Liberia, 25.vii.1965, P. J. Spangler, 32 males, 12 females (pinned), 1 male, 2 females (alcohol) (NMNH); Pacuare, Río General, 1.vii.1967, Flint & Ortiz, 8 males, 2 females (pinned) (NMNH); 9 mi NW Esparta, 22.vii.1965, P. J. Spangler, 2 males, 1 female (alcohol) (NMNH); **Alajuela:** Río Pizote, ca 5 km (air) S Brasilia, 10°58.320'N, 85°20.700'W, el 390 m, 12.iii.1986, Holzenthal & Fasth, 2 males (pinned), 20 males, 15 females (alcohol) (UMSP); Río Pizote, ca 5 km N Dos Ríos, 10°56.880'N, 85°17.460'W, el 470 m, 9.iii.1986, Holzenthal & Fasth, 2 males, 1 female (pinned), 64 males, 116 females (alcohol) (UMSP); Reserva Forestal San Ramon, Río San Lorencito and tribs., 10°12.960'N, 84°36.420'W, el 980 m, 30.iii.–i.iv.1987, Holzenthal, Hamilton, Heyn, 1 male (alcohol) (UMSP); Laguna Río Cuarto & trib., 2.8 km (road) N Río Cuarto, 10°21.420'N, 84°12.900'W, el 400 m, 13.ii.1992, Holzenthal, Muñoz, Kjer, 1 male, 3 females (pinned) (UMSP); **Cartago:** Reserva Tapantí, Quebrada Segunda @ admin. Building, 9°45.660'N, 83°47.220'W, el 1250 m, 9–10.v.1990, Holzenthal & Blahnik, 2 males (alcohol) (UMSP); **Guanacaste:** Colorado, 31.iii.1988, W. E. Steiner, J. M. Hill J. M. Swearingen, & J. M. Mitchell, 2 males, 2 females (NMNH); Río Tizate, 7.2 km NE Canas Dulces, 10°46.380'N, 85°26.940'W, el 275 m, 28.vi.1986, Holzenthal, Heyn, Armitage, 2 males (pinned), 50 males, 75 females (alcohol) (UMSP); Río Mena, 4.2 km W Santa Cecelia, 11°3.540'N, 85°26.880'W, el 260 m, 11.iii.1986, Holzenthal & Fasth, 1 male (pinned), 9 males, 11 females (alcohol) (UMSP); Quebrada Garcia, ENE Quebrada Grande, 10°51.720'N, 85°25.680'W, el 470 m, 8.iii.1986, Holzenthal & Fasth, 7 males (pinned), 7 males, 8 females (alcohol) (UMSP); Río Los Ahogados, 11.3 km ENE Quebrada Grande, 10°51.900'N, 85°25.380'W, el 470 m, 7.iii.1986, Holzenthal & Fasth, 1 male (pinned), 7 males, 3 females (alcohol) (UMSP); Parque Nacional Guanacaste, Maritza, Río Tempisquito Sur, 10°57.000'N, 85°28.800'W, el 600 m, 30.viii.1990, Huisman & Quesada, 6 males, 10 females (alcohol) (UMSP); Río Tempisquito, ca 3 km S route 1, 10°47.400'N, 85°33.120'W, el 75 m, 6.iii.1986, Holzenthal & Fasth, 9 males, 2 females (alcohol) (UMSP); Río Aguacate, 0.5 km E Aguacate, 10°33.900'N, 84°56.340'W, el 590 m, 16.ii.1992, Holzenthal, Muñoz, Kjer, 1 male (pinned) (UMSP); **Heredia:** same data as holotype, 10 males, 36 females (pinned), 5 males, 7 females (alcohol) (UMSP); Río Bijagual, on road to Magsasay, 10°24.480'N, 84°4.560'W, el 140 m, 12.ii.1986, Holzenthal, Morse, Fasth, 2 males, 1 female (alcohol) (UMSP); Río Bijagual, 3.5 km S Chilamate, 10°26.160'N, 84°3.600'W, el 40 m, 1.vii.1992, F. Muñoz, 9 males (pinned) (UMSP); Río Sarapiquí, 300 m S Chilamate, 10°27.060'N, 84°3.840'W, el 40 m, 30.vi.1992, F. Muñoz, 11 males, 1 female (pinned) (UMSP); Est. Biol. La Selva, Río Puerto Viejo, 10°26.400'N, 84°0.720'W, el 30 m, 10–11.ii.1986, Holzenthal, 3 males, 1 female (pinned) (UMSP); same location, 19.vi.1986, Holzenthal, Heyn, Armitage, 4 males, 6 females (alcohol) (UMSP); Est. Biol. La Selva, Quebrada El Salto, 10°25.620'N, 84°0.300'W, el 50m, 10.ii.1986, Holzenthal, 2 males (pinned) (UMSP); Est. Biol. La Selva, Quebrada Sura, 10°26.220'N, 84°0.600'W, el 50 m, 20–21.vi.1986, Holzenthal, Heyn, Armitage, 5 males (alcohol) (UMSP); Río Sarapiquí, 7 km W Puerto Viejo, 10°27.120'N, 84°4.020'W, el 50 m, 11.ii.1986, Morse & Fasth, 16 males, 2 females (pinned), 29 males, 14 females (alcohol) (UMSP); Quebrada Ceibo, 6 km E Cháves, 10°22.920'N, 83°55.320'W, el 50 m, 2.vii.1992, F. Muñoz, 1 male (pinned) (UMSP); **Limón:** Reserva Biol. Hitoy-Cerere, Río Cerere, 9°40.260'N, 83°1.680'W, el 90 m, 23–24.iii.1987, Holzenthal, Hamilton, Heyn, 1 male, 1 female (pinned) (UMSP); E.A.R.T.H., Río Parismina, 10°14.880'N, 83°34.200'W, el 5 m, 4.ii.1992, Holzenthal, Muñoz, Kjer, 55 males, 5 females (pinned), 12 males, 3 females (alcohol) (UMSP) (INBIO); E.A.R.T.H., Río Destierra, Pozo Azul, 10°12.480'N, 83°34.440'W, el 15 m, 5.ii.1992, Holzenthal, Muñoz, Kjer, 23 males, 13 females (pinned), 6 males, 11 females (alcohol) (UMSP); same location, 27.vi.1992, F. Muñoz, 5 males, 1 female (pinned) (INBIO); E.A.R.T.H., Río Dos Novillos, 10°13.200'N, 83°35.460'W, el 20 m, 3.ii.1992, Holzenthal, Muñoz, Kjer, 19 males, 10 females (pinned) (UMSP); Res. Biol. Barbilla, Río Dantas, 15 km (road) S Pacuarito, 9°59.640'N, 83°26.580'W, el 300 m, 27–30.i.1992, Holzenthal, Muñoz, Kjer, 2 males (pinned) (UMSP); **Puntarenas:** Río Jaba at rock quarry, 1.4 km (air) W Las Cruces, 8°47.400'N, 82°58.200'W, el 1150 m, 15.iii.1991, Holzenthal, Muñoz, Huisman, 3 males, 4 females (pinned) (UMSP); Río Jaba, 2.4 km (air) NW San Vito, 8°49.920'N, 82°59.460'W, el 970 m, 13.vi.1986, Holzenthal, Heyn, Armitage, 1 male, 7 females (alcohol) (UMSP); Río Negro, ca 10 km (air) SSW Las Alturas, 8°57.000'N, 82°51.060'W, el 1035 m, 17.ii.1986, Holzenthal, Morse, Fasth, 5 males, 1 females (pinned) (UMSP); Río Guineal, ca. 1 km (air) E Finca Helechales, 9°4.560'N, 83°5.520'W, el 840 m, 4.viii.1987, Holzenthal, Morse, Clausen, 2 males (alcohol) (UMSP); Río Singri, ca. 2 km (air) S Finca Helechales, 9°3.420'N, 83°4.920'W, el 720 m, 21.ii.1986, Holzenthal, Morse, Fasth, 2 males (alcohol) (UMSP); Río Rincón, 6.5 km (air) S Rincón, 8°38.280'N, 83°28.800'W, el 20 m, 7.iv.1987, Holzenthal, Hamilton, Heyn, 1 male (alcohol) (UMSP); Río Cotón in Las Alturas, 8°56.280'N, 82°49.560'W, el 1360 m, 12.viii.1990, Holzenthal, Blahnik, Muñoz, 2 males, 3 females (pinned) (UMSP); Río Bellavista, ca 1.5 km NW Las Alturas, 8°57.060'N, 82°50.760'W, el 1400 m, 10–11.viii.1990, Holzenthal, Blahnik, Muñoz, 1 male, 1 female (pinned) (UMSP); Río Ceibo, route 2, ca 6 km W rd to Buenos Aires, 9°8.940'N, 83°22.620'W, el 250 m, 20.ii.1986, Holzenthal, Morse, Fasth, 1 male (pinned), 114 males, 116 females (alcohol) (UMSP); Res Biol. Carara, Río Carara, 4.3 km (rd) E Costanera Sur, 9°48.600'N, 84°34.320'W, el 20 m, 12.iii.1991, Holzenthal, Muñoz, Huisman, 1 male, 1 female (alcohol) (UMSP); Río Cataracta, 5.5 km S Brujo (Carara Zona Sur), 9°3.840'N, 83°16.320'W, el 120 m, 6–7.vii.1992, F. Muñoz, 4 males, 1 female (pinned) (UMSP); Río Platanar, Salitre, 6.5 km NE Buenos Aires, 9°11.700'N, 83°16.860'W, el 450 m, 8–9.vii.1992, F. Muñoz, 17 males, 13 females (pinned) (UMSP); **San José:** P. N. Braulio Carrillo, Est. Carrillo, Quebrada El Molinete, 10°9.600'N, 83°57.780'W, el 700 m, 22–28. viii.1986, I. & A. Chacón, 21 males, 7 females (pinned) (UMSP); Res. Biol., Carara, Río Carara in Carara, 9°46.680'N, 84°31.860'W, el 200 m, 14.iii.1991, Holzenthal, Muñoz, Huisman, 3 males (alcohol) (UMSP); Res. Biol. Carara, Río del Sur, 1.5 km (rd) S Carara, 9°46.140'N, 84°31.860'W, el 160 m, 13.iii.1991, Holzenthal, Muñoz, Huisman, 3 males (pinned), 1 male (alcohol) (UMSP); Río Negro, 3.5 km SE jct. route 239, 9°40.800'N, 84°23.640'W, el 230 m, 21.iii.1986, Holzenthal & Fasth, 14 males, 10 females (alcohol) (UMSP); Río General, 1 mi S San Isidro, 1.vii.1967, P.J. Spangler, 1 male (alcohol) (NMNH); **PANAMA:** El Valle, 15.vii.1967, O.S. Flint, Jr., 1 male (pinned) (NMNH); **Canal Zone:** Pipeline Rd., Río Agua Salud, 8–12.vii.1967, Flint & Ortiz, 4 males (pinned) (NMNH); Gamboa, Río Agua Salud, --vii.1967, W.W. Wirth, 1 male (alcohol) (NMNH); Río Agua Salud, 30.iii.1965, S. & W.D. Duckworth, 1 male, 3 females (alcohol) (NMNH); **San Blas:** Río Carti Grande, 2 km W Nusagandi, 5.iii.1985, Flint & Louton, 1 male (pinned) (NMNH); **COLOMBIA: Magdalena:** Municipio de Santa Marta, Río Minca en Minca, 11°8.584'N, 74°6.967'W, el 570 m, 9.xii.1997, F. Muñoz-Q, et al., 3 males, 1 female (pinned) (UMSP); **Tolima:** Armero, nr Guayabal, 2–10.ii.1977, E.L. Peyton, 7 males, 3 females (pinned), 42 males, 18 females (alcohol) (NMNH); **Valle:** Municipio de Buenaventura, Río Escalerte, frente a casa de “AcuaValle”, ca. 15 km SE Cordoba, 3°49.633'N, 76°52.250'W, el 200 m, 1.xii.1997, F. Muñoz-Q. et al., 1 male, 1 female (pinned) (UMSP); **ECUADOR: Loja:** Macara, 13.viii.1977, L.E. Peña G., 1 male (pinned) (NMNH); Río Puyango, el 300 m, 17–18.viii.1977, L.E. Peña G., 1 male (pinned), 1 male (alcohol) (NMNH); **Pichincha:** Santo Domingo de los Colorados, 14 km E, 5.vii.1975, Langley & Cohen, 1 male, 1 female (pinned) (NMNH).

#### Etymology.

This species is named *Oecetis protrusa* for the protruding ventral apex of the phallobase in this species.

### Oecetis sordida sp. n.

http://zoobank.org/157D0A4E-303D-4DB0-8B8D-A46A782C7CD9

http://species-id.net/wiki/Oecetis_sordida

**Figure 23.**
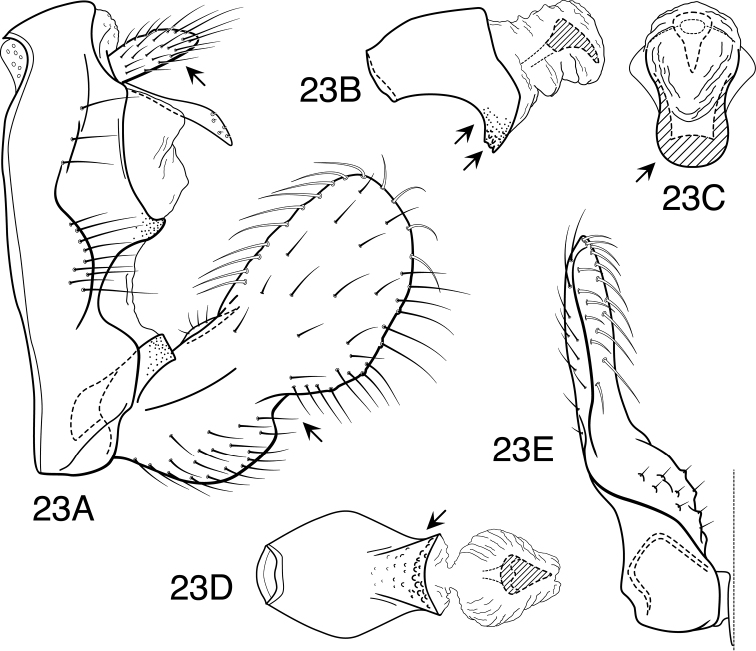
*Oecetis sordida* sp. n., male genitalia: **A** lateral **B** phallic apparatus, lateral **C** phallic apparatus, caudal **D** phallic apparatus, ventral **E** inferior appendage, caudal.

Figs 23 , 38 , Map 2

Oecetis disjuncta (Banks): Smith and Lehmkuhl 1980: 638 (Figs 1–2, 5, 7, 9–10, 12, 14, 16, 18, 21, 23, 25–26, 29–34, 37–38, 40–41, 43, 45, 46–48, 50); Floyd 1995: 29 (Figs 22A-H, 25) [larval description and distribution, at least in part].

#### Diagnosis.

This is the species that has been generally referred to in the literature under the name *Oecetis disjuncta*. As compared to *Oecetis disjuncta*, which is known to us by only a few specimens from California and Oregon, *Oecetis sordida* has a very wide distribution, occurring in southern Mexico and over much of the western part of the United States, with records as far east as Michigan (Smith and Lehmkuhl 1980). The species shows little variation in size, coloration, or genital morphology over this wide range. As defined here, the species can be diagnosed from other species in the *Oecetis avara* group by its large size, dark brown color, evidently pigmented forewing chord, and by the distinctly deflexed or down-turned apex of the phallobase, which is U-shaped as viewed caudally and has a diagnostic warty ridge (or at least a roughened, rugose texture), preapically on its ventral margin, much as that described for *Oecetis verrucula* sp. n. Although the overall genitalic morphology of *Oecetis sordida* is very similar to *Oecetis disjuncta*, particularly in the shape of the inferior appendages and the angularly bent phallobase, it differs in having much shorter preanal appendages (half the overall length of those in *Oecetis disjuncta*), and also by its darker coloration (dark brown with a distinctly pigmented forewing chord, instead of yellowish-brown with a forewing chord unpigmented or only weakly pigmented), and by the presence of the warty ridge on the phallobase, as discussed above, which is absent in *Oecetis disjuncta*. *Oecetis sordida* is also somewhat similar to *Oecetis apache*, sp. n., *Oecetis metlacensis*, and *Oecetis uncata* sp. n. especially in size and the general structure of the inferior appendages and phallobase. However all of these species have the apex of the phallobase V-shaped in caudal view, rather than U-shaped, and all are also much lighter in color, yellowish, yellowish-brown, or light brown, rather than dark brown. *Oecetis sordida* also occurs in sympatry with *Oecetis houghtoni* sp. n. The 2 could be confused due to their relatively similar coloration and presence in both of a pigmented forewing chord (variably developed in *Oecetis houghtoni*). However, *Oecetis houghtoni* is smaller in size, lighter in color, and lacks either a distinctly angled phallobase or preapical wart on the structure. Additionally, it possesses an asymmetric phallic sclerite, absent in *Oecetis sordida*.

#### Adult.

Forewing length: male (11.0–12.5 mm), female (9.8–10.6 mm). Color medium to dark brown, legs slightly paler, palps grayish brown, overall appearance somewhat “hoary” due to intermixed paler setae. Antennae very pale brown with indistinct, narrow annulations at intersection of antennomeres. Forewing spots small and indistinct, spots at base of discal and thyridial cells and base of fork V most noticeable, more or less V-shaped (confined to base of fork); veins of forewing chord distinctly pigmented and relatively widely spaced, *s* and *r-m* veins usually slightly closer; apical spots, at apices of major veins absent or very inconspicuous. Setae along veins in apical part of forewing semi-prostrate, laterally diverging, not conspicuous due to relatively dense setae on membrane. Fringe of setae along costal margin of forewing short, dense, decumbent.

#### Male genitalia.

Segment IX very short, with elongate setae along posterolateral margin. Tergum X with narrow, deflexed mesal lobe, lobe very short, tapering apically, apex with small sensilla; lobe continuous basoventrally with paired lateral membranous projections. Preanal appendage relatively short, length about 2 1/2 times maximum width, simple in structure, apical setae elongate. Inferior appendage with very prominent rounded dorsal lobe and scarcely projecting ventral lobe, separated from dorsal lobe by shallow, rounded notch; posterior margin of ventral lobe, as viewed ventrally, with rounded bend near base, lobes very weakly diverging; basomesal projection of appendage forming very weakly developed projection with short, stiff setae; dorsal lobe with very stout, ventrally-projecting setae on anterior margin, longer, finer setae on posterior margin and mesally-curved setae on dorsal margin, mesal surface with stout, ventrally curved setae. Phallobase relatively short, ventral apex strongly, angularly deflexed, bend close to apex, apex distinctly sclerotized, ventral surface with distinct rugose region preapically (Figs 23B, D), rugose area often somewhat raised as bur or wart; ventral apex, as viewed caudally, broadly U-shaped (Fig. 23C). Phallotremal sclerite prominent, basally forming relatively large tubular collar, ventral margin projecting; asymmetrical lateral sclerite absent.

#### Holotype.

**Male** (pinned): **USA: South Dakota:** Lawrence Co., Black Hills National Forest, Boxelder Creek, 1.8 mi W Nemo, 44°11.846'N, 103°32.024'W, el 1470 m, 11.viii.1994, Holzenthal & Huisman (UMSP) (UMSP000065014).

#### Paratypes.

**MEXICO: Chihuahua:** Riito, Hwy 16, 10 mi E Yepachic, 23.vi.1987, B. Kondratieff & R.W. Baumann, 1 male (pinned) (NMNH); **Durango:** 10 mi. W El Salto, 9000 ft. 1.vii.1964, J.E.H. Martin, 1 male (pinned) (NMNH); **Oaxaca:** 1 mi NE of Ixtlan de Juarez, 13.viii.1967, O.S. Flint, Jr., 1 male (pinned) (NMNH); **Mexico:** Temascaltepec, Real de Arriba, 2.vi.1990, A. Rojas, 2 males (pinned) (UMSP); **USA: Arizona:** Oak Cr. Canyon, 18.vi.1968, Flint & Menke, 1 male (pinned) (NMNH); Apache Co., Little Colorado R., Greer, 34°01'N, 109°45'W, el 2530 m, 13.viii.1995, C.M. & O.S. Flint, Jr., 1 male (pinned) (NMNH); Apache Co., W Fork Black River, F.R. 68 @ West Fork Campground, 33°46.676'N, 109°24.291'W, el 2438 m, 18.vi.1999, D.C. Houghton, 38 males (pinned) (UMSP); Apache Co., W. Fork L. Colorado River, end of F.R. 575, nr. Greer, 33°59.627'N, 109°27.890'W, el 2713 m, 16.vi.1999, D.C. Houghton, 1 female (pinned) (UMSP); Apache Co., S. Fork L. Colorado R., F.R. 560 @ South Fork Campground, 34°04.690'N, 109°24.629'W, el 2347 m, 27.vi.1999, D.C. Houghton, 2 males (pinned) (UMSP); Apache Co., W. Fork Black R., F.R. 68 @ West Fork Campground, 33°46.676'N, 109°24.291'W, 2438 m, 18.vi.1999, D.C. Houghton, 5 males (pinned) (UMSP); same except 19.vi.1999, D.C. Houghton, 21 males (alcohol) (UMSP); Apache Co., Apache-Sitgreaves National Forest, San Francisco River, Luna Lake spillway., FR. 570, nr. Luna Lake campground, 33°49.678'N, 109°04.855'W, el 2408 m, 28.vi.1999, D.C. Houghton, 1 female (alcohol) (UMSP); Greenlee Co., Apache-Sitgreaves National Forest, Blue R. @ F.R. 281, 7 km SE Upper Blue Campground, 33°39.807'N, 109°06.507'W, el 1768 m, 20.vi.1999, D.C. Houghton, 34 males (alcohol), 2 males (pinned) (NMNH); Greenlee Co., Apache-Sitgreaves National Forest, Blue River @ F.R. 475, Blue Range Primitive Area, 33°17'N, 109°11'W, el 1280 m, 21.vi.1999, D.C. Houghton, 1 male (pinned), 1 male (alcohol) (UMSP); **Minnesota:** Lake Co., shore Lake Superior, at Split Rock, 1.vii.1935, D.G. Denning, 1 male (pinned) (UMSP); Lake Co., W. Fork Split Rock River, County Road 03, 47°14.333'W, 91°29.000'W, 21.vii.2001, D.C. Houghton, 5 males (alcohol) (UMSP); Cook Co., Cascade Creek, Cascade River S.P., Superior Hiking Trail, 47°42.500'N, 90°31.666'W, 9.vii.2001, D.C. Houghton, 1 male (alcohol) (UMSP); Washington Co., Valley Creek, Belwin Station., 44°55.000'N, 92°47.667'W, 15.vii.2001, D.C. Houghton, 2 males (alcohol) (UMSP); St. Louis Co., shore Lake Superior at Split Rock, 1.vii.1935, D.G. Denning, 1 male (pinned) (UMSP); **South Dakota:** same data as holotype, 1 male (pinned) (UMSP); **Montana:** Beaverhead Co., Beaverhead National Forest, Pettengill Cr., 9.7 mi S Wise River, 45°40.843'N, 113°03.606'W, el 1930 m, 6.viii.2006, Holzenthal, Blahnik, Robertson, Huisman, 1 male (pinned) (UMSP); Beaverhead Co., Beaverhead National Forest, Big Hole River, W of Divide on State Hwy 43, 45°45.400'N, 112°46.850'W, 25.vi.2002, R.J. Blahnik, 11 males, 6 females (pinned) (UMSP); **Wyoming:** Sublette Co., Half Moon Lake, 42°56'N, 109°45'W, el 2315 m, 24.vii.1995, C.M. & O.S. Flint, Jr., 1 male (pinned) (NMNH); Sublette Co., Bridger Teton National Forest, Half Moon Lake, 42°55.857'N, 109°44.462'W, el 2130 m, 24.vii.1995, Holzenthal, et al., 1 female (pinned) (UMSP).

#### Etymology.

This species is named *Oecetis sordida* from the Latin word *sordidus*, used in Botanical and Zoological literature to describe a dull, dirty, or muddy hue, and referring here to the dark color of the wings of this species.

### Oecetis tumida sp. n.

http://zoobank.org/8A57E9CC-856F-4D31-B012-896287F0924C

http://species-id.net/wiki/Oecetis_tumida

**Figure 24.**
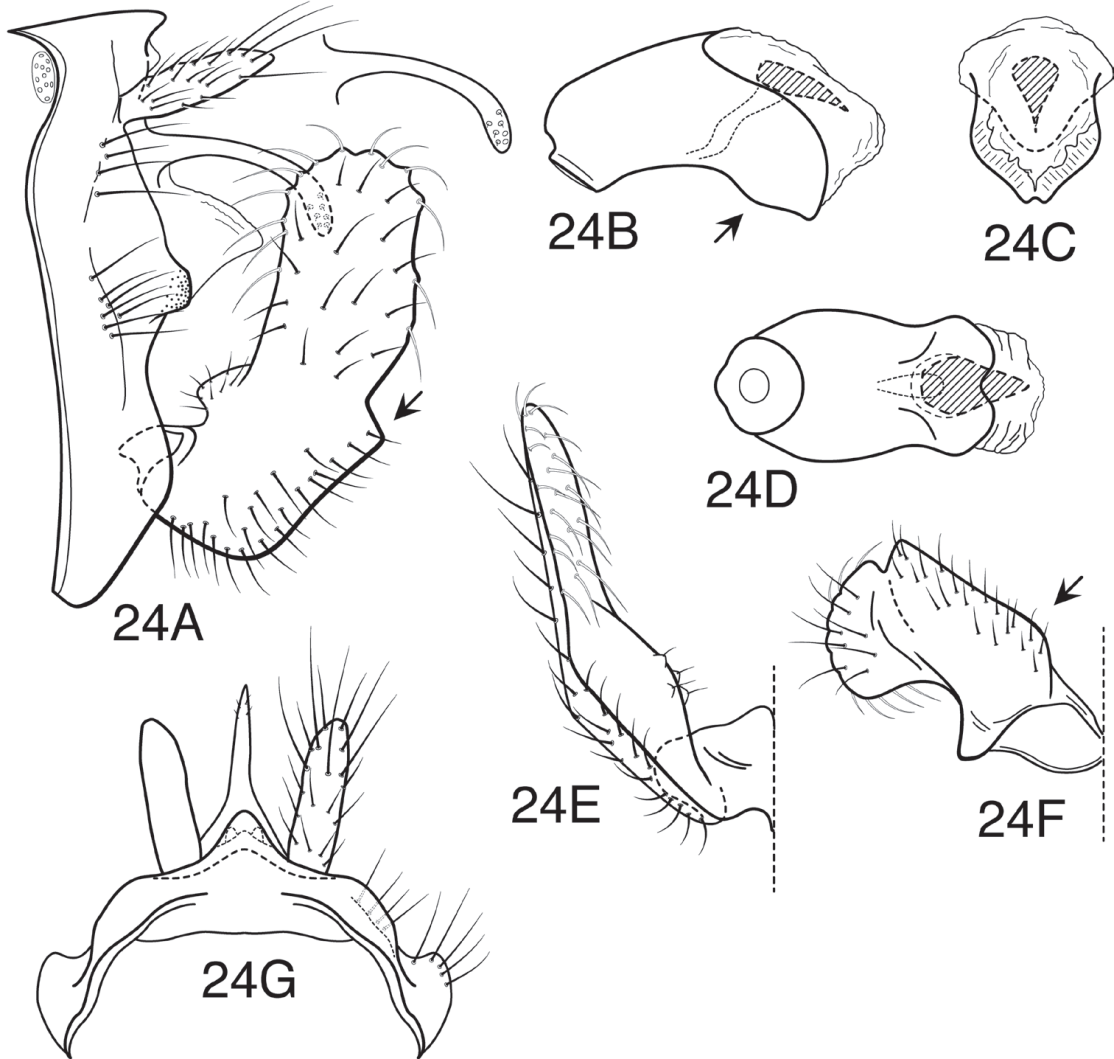
*Oecetis tumida* sp. n., male genitalia: **A** lateral **B** phallic apparatus, lateral **C** phallic apparatus, caudal **D** phallic apparatus, ventral **E** inferior appendage, caudal **F** inferior appendage, ventral **G** egment IX and tergum X, dorsal.

Figs 24 , 48 , Map 1

#### Diagnosis.

*Oecetis tumida* is more or less intermediate in characters between *Oecetis angularis* sp. n. and *Oecetis protrusa* sp. n., agreeing with the former in the general shape of the inferior appendages, and with the latter in having the ventral apex of the phallobase somewhat convexly bulged preapically. The holotype specimen is from a population that is sympatric with *Oecetis protrusa*. In general, though, the phallobase is shorter and more bulbous apically than in *Oecetis protrusa*. In addition to the genitalic differences, the specimens were slightly larger than the co-occurring specimens of *Oecetis protrusa*. The species is most likely to be confused with *Oecetis angularis*, in part due to the similarity in color and size of the 2 species, but especially due to the shape of the inferior appendages, which have an obtusely angled ventral lobe that is very weakly, but angularly projecting. The convex preapical bulge on the phallobase in *Oecetis tumida*, and the absence of a distinctly V-shaped apex, in caudal view, should distinguish them.

#### Adult.

Forewing length: male (9.0–10 mm) Color pale yellowish to slightly brownish-yellow (similar to *Oecetis mexicana*); forewing spots distinct, spots at base of discal and thyridial cells large or moderately large, other spots smaller; veins of forewing chord widely spaced, *r* and *r-m* veins slightly closer; chord with small spots at juncture of major veins, spots at opposite ends of chord slightly larger; spots at apices of major veins small but distinct, pigmentation largely confined to vein. Setae along veins in apical part of forewing elongate, semi-prostrate, laterally diverging. Fringe of setae along costal margin of forewing moderate in length, somewhat projecting, not conspicuously erect.

#### Male genitalia.

Segment IX very short, with elongate setae along posterolateral margin. Tergum X with narrow, deflexed mesal lobe, lobe relatively elongate, somewhat lobulate (expanded) apically, apex with small sensilla; lobe continuous basoventrally with short paired lateral membranous projections. Preanal appendage moderately elongate, length about 3 times maximum width, simple in structure, apical setae elongate. Inferior appendage with rounded dorsal lobe and angularly projecting ventral lobe; ventral lobe very weakly projecting, forming approximately right to distinctly obtuse angle with dorsal lobe, posterior margin of ventral lobe, as viewed ventrally (Fig. 24F), with distinctly angular bend near base; basomesal projection weakly developed, forming short rounded projection with short, stiff setae; dorsal lobe with stout, mesally-curved setae on dorsal margin and stout, ventrally-curved setae on mesal surface. Phallobase, as viewed laterally, relatively short, distinctly inflated apically; as viewed laterally with weakly developed, convex bulge preapically on ventral margin; as viewed caudally, with apex inflated, broadly U-shaped or V-shaped, not keeled ventrally (Fig. 24C). Phallotremal sclerite prominent, basally forming short tubular collar, ventral margin projecting, apex acute; asymmetrical lateral sclerite apparently absent.

#### Holotype.

**Male** (pinned), **COSTA RICA: Limón:** Res. Biol. Barbilla, Río Dantas, 15 km (road) S Pacuarito, 9°59.640'N, 83°26.580'W, el 300 m, 27–30.i.1992, Holzenthal, Muñoz, Kjer (UMSP) (INBIOCRI000372044).

#### Paratypes.

**COSTA RICA: Guanacaste:** Quebrada Garcia, 10. 6 km ENE Quebrada Grande, 10.862°N, 85.428°W, 470 m, 8.iii.1986, Holzenthal & Fasth, 1 male, 1 female (alcohol) (NMNH); Río Los Ahogados, 11.3 km ENE Quebrada Grande, 10.865°N, 85.423°W, el 470 m, 7.iii.1986, Holzenthal & Fasth, 1 male, 2 females (alcohol) (UMSP); **Limón:** same data as holotype, 1 male (pinned) (UMSP); **Puntarenas:** Río Singri, ca. 2 km (air) S Finca Helechales, 9°3.420'N, 83°4.920'W, el 720 m, 21.ii.1986, Holzenthal, Morse, Fasth, 1 male (alcohol) (UMSP).

#### Etymology.

This species is named *Oecetis tumida*, after the Latin word *tumidus*, meaning swollen, and referring to the apex of the phallobase in this species, which is distinctly swollen and usually has a slight, but noticeable, preapical ventral bulge.

### Oecetis uncata sp. n.

http://zoobank.org/8176D160-6595-4A6C-AC05-C0817346BC07

http://species-id.net/wiki/Oecetis_uncata

**Figure 25.**
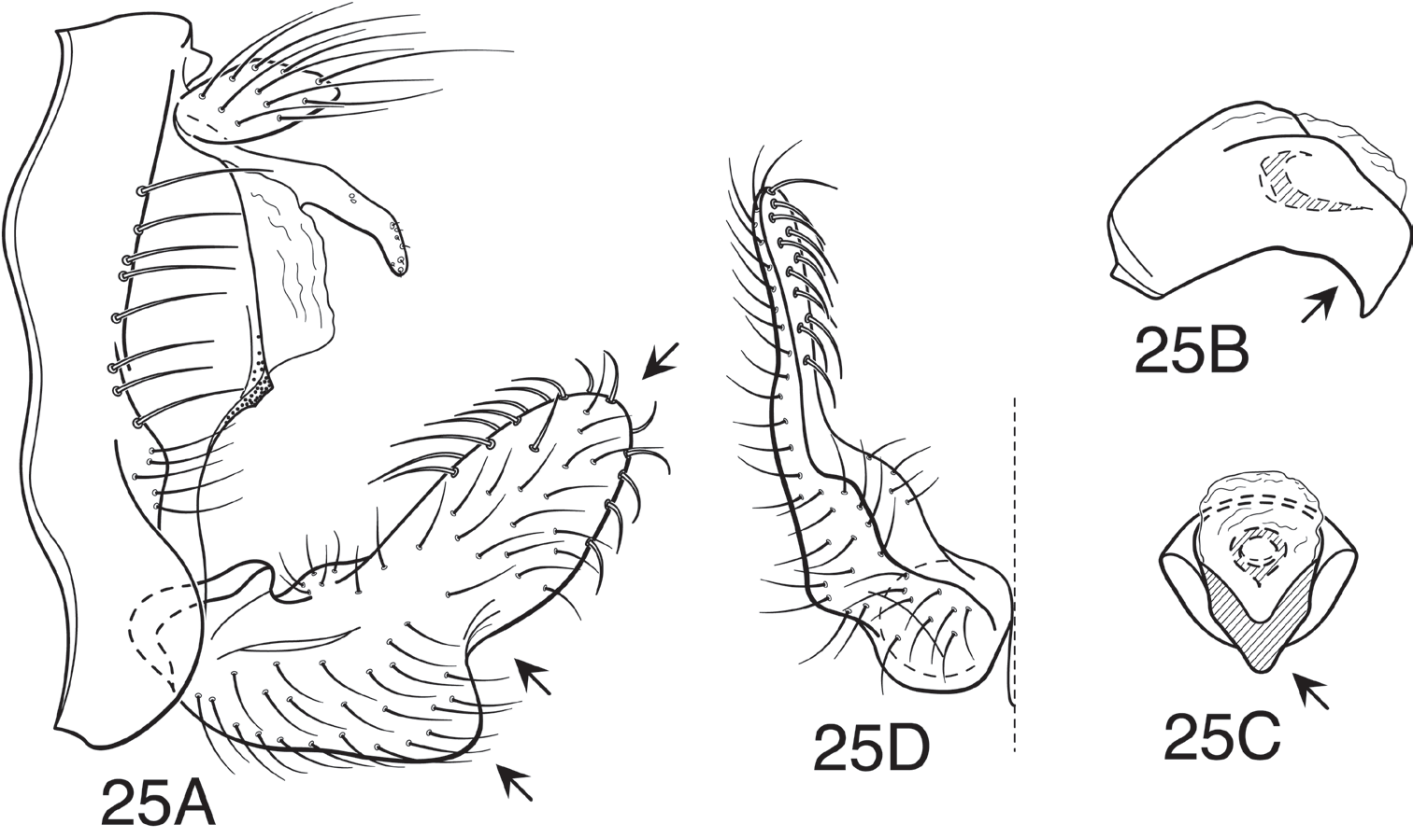
*Oecetis uncata* sp. n., male genitalia: **A** lateral **B** phallic apparatus, lateral **C** phallic apparatus, caudal **D** inferior appendage, caudal.

Figs 25 , 35 , Map 5

#### Diagnosis.

*Oecetis uncata* sp. n. is larger than any of the other species of the *Oecetis avara* group with which it is sympatric, with the exception of *Oecetis metlacensis*, which is similar in both size and general color (not always as distinct as in Figs 35 and 36). Both species also have the apex of the phallobase V-shaped, as viewed caudally. However, despite the general similarity in color and genitalia, the forewing spots of *Oecetis uncata* are more rounded and prominent in size than in *Oecetis metlacensis* and there are several genitalic differences. In general, the populations of *Oecetis metlacensis* recorded from Costa Rica and Mexico, despite their several differences (see remarks section under *Oecetis metlacensis*), resemble each other more closely than either do *Oecetis uncata*. From *Oecetis metlacensis*, *Oecetis uncata* can be distinguished by the shape of the phallobase and also by the inferior appendages of the male. The phallobase is deflected or downturned more apically in *Oecetis uncata* than in *Oecetis metlacensis* and the inferior appendage has a ventral lobe that is more distinctly angled and projecting, and has a less prominent basomesal process. At present *Oecetis uncata* is known from only a restricted area in Costa Rica.

#### Adult.

Forewing length: male (11.8–13.0 mm), female (10.0–10.5 mm). Color brownish-yellow (slightly darker than *Oecetis mexicana*, similar to *Oecetis metlacensis*); forewing spots distinct, spots at base of discal and thyridial cells and base of fork V largest, moderate in size, slightly ovate, other spots small; veins of forewing chord widely spaced, *r* and *r-m* veins usually slightly closer; chord with small spots at juncture of major veins; spots at apices of major veins small but distinct, pigmentation largely confined to vein. Setae along veins in apical part of forewing elongate, semi-prostrate, laterally diverging. Fringe of setae along costal margin of forewing relatively short, dense, not conspicuously erect.

#### Male genitalia.

Segment IX very short, with elongate setae along posterolateral margin. Tergum X with narrow, deflexed mesal lobe, lobe moderate in length, nearly uniform in width, tapering apically, apex with small sensilla; lobe continuous basoventrally with short, paired lateral membranous projections. Preanal appendage relatively short, length about 2 times maximum width, simple in structure, apical setae elongate. Inferior appendage with prominent rounded dorsal lobe and angularly projecting ventral lobe; dorsal lobe somewhat narrowed dorsally, ventral lobe distinctly projecting, forming approximately right to somewhat obtuse angle with dorsal lobe, apex of ventral lobe rounded, not angular; mesal margin of ventral lobe, as viewed ventrally, only weakly bent near base, apices not strongly diverging; basomesal projection weakly developed, forming short rounded projection with short, stiff setae; dorsal lobe with stout ventrally curved setae on anterior margin, mesally-curved setae on dorsal margin and stout, ventrally-curved setae on mesal surface. Phallobase, as viewed laterally, relatively short, apex very angularly bent or hooked; apex, as viewed caudally, with ventral margin distinctly V-shaped, only weakly keeled ventrally (Fig. 25C). Phallotremal sclerite prominent, basally forming short tubular collar, ventral margin projecting, apex acute; asymmetrical lateral sclerite absent.

#### Holotype.

**Male** (pinned), **COSTA RICA: Cartago:** Reserva Tapantí, Río Dos Amigos & falls, ca. 6 km (rd) NW tunnel, 9°42.240'N, 83°46.980'W, el 1500 m, 23.iii.1991, Holzenthal, Muñoz, Huisman (UMSP) (UMSP000091730).

#### Paratypes.

**COSTA RICA: Cartago:** same location as holotype, 20.vi.1992, F. Muñoz, 1 male, 1 female (pinned) (UMSP); Reserva Tapantí, Río Grande de Orosi, 9°41.160'N, 83°45.360'W, el 1650 m, 18–21.iii.1987, Holzenthal, Hamilton, Heyn, 4 males (pinned) (UMSP); same location, 8–9.vii.1986, Holzenthal, Heyn, Armitage, 10 males, 10 females (pinned); (UMSP); same location, 15–16.vii.1987, Holzenthal, Morse, Clausen, 2 males (pinned) (UMSP); same location, 7–8.vi.1988, C.M. & O.S. Flint, Holzenthal, 6 males (NMNH); Refugio de la Fauna Tapantí, Río Grande de Orosi at Puente Dos Amigos, el 1600 m, 20.vi.1992, Contreras & Muñoz, 1 male (pinned) (UMSP); **San José:** Río Parrita Chiquito, rt. 12, 6.5 km SW jct. rt. 2, 9°42.180'N, 83°58.200'W, el 1980 m, 10.iv.1987, Holzenthal, Hamilton, Heyn, 1 male (pinned) (UMSP).

#### Etymology.

This species is named *Oecetis uncata* from the Latin word *uncus*, a hook, and referring here to the hooked apex of the phallobase of this species.

### Oecetis verrucula sp. n.

http://zoobank.org/7C5F98FC-0131-40A1-B9DB-A022D04F937E

http://species-id.net/wiki/Oecetis_verrucula

**Figure 26.**
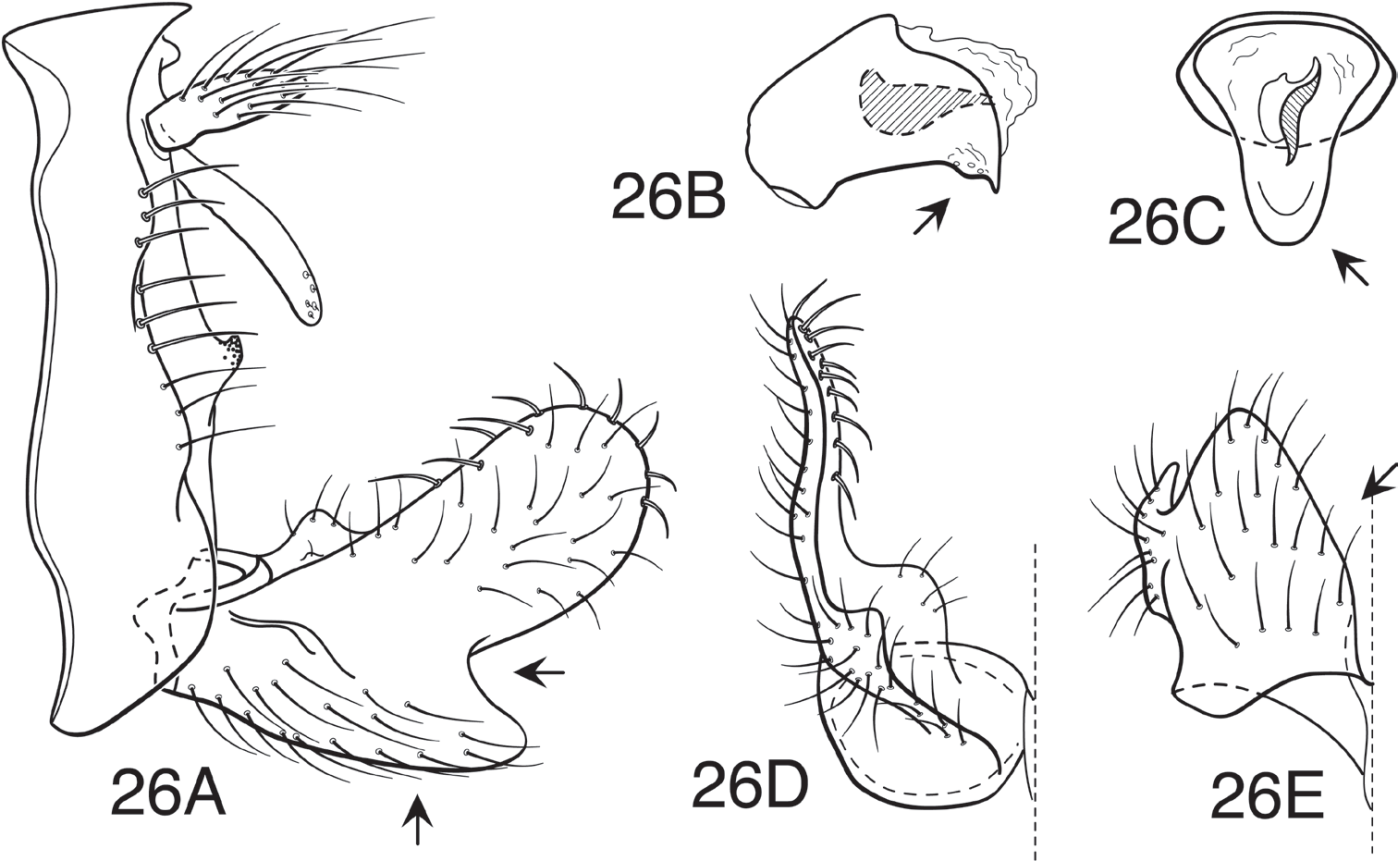
*Oecetis verrucula* sp. n., male genitalia: **A** lateral **B** phallic apparatus, lateral **C** phallic apparatus, caudal **D** inferior appendage, caudal **E** inferior appendage, ventral.

Figs 26 , 44 , 57 , Map 5

#### Diagnosis.

This species is nearly identical in color and in the shape of the inferior appendages to *Oecetis avara*. Character similarities include, particularly, a prominent ventral lobe of the inferior appendage, which is very distinctly acutely angled with respect to the dorsal lobe, and also the general shape of the phallobase, which is short and strongly arched ventrally. The only definitive diagnostic character separating this species from *Oecetis avara* is the presence in *Oecetis verrucula* of a small preapical “rugose wart” on the ventral surface of the phallobase. *Oecetis avara* lacks this character, but may have the extreme apex of the phallobase slightly burred or ridged. A character state similar to that in *Oecetis verrucula* occurs in *Oecetis sordida* sp. n., which is otherwise quite different in morphology. Considering the relatively minor nature of the difference, it is possible that *Oecetis verrucula* should be considered a variant of *Oecetis avara*. However, because the range of variability in *Oecetis avara* still needs to be assessed, and also because the character state in *Oecetis verrucula* is consistent over a wide distribution in which it is nearly invariant, we thought it best to assign this form to its own species.

This species is most readily diagnosed by its coloration, yellowish with a tinge of brown, darker than *Oecetis mexicana* sp. n., and with relatively small and generally somewhat oval forewing spots, by the very prominent, acutely angled ventral lobe of the inferior appendage, and by the shape and structure of the phallobase, which has a small preapical “wart” on its ventral surface. The last character is considered diagnostic. Species that are nearly similar in size and occur in sympatry, *Oecetis angularis*, *Oecetis protrusa*, or *Oecetis mexicana*, are likely to have the forewing spots distinctly rounded and the inferior appendages, as viewed ventrally, to be more distinctly angled basally (compare Fig. 26E with Figs 3E, 22E, and 18F). Among these, *Oecetis verrucula* is most likely to be confused with *Oecetis protrusa*, because both have the inferior appendage acutely angled. *Oecetis protrusa* additionally differs from *Oecetis verrucula* in that the apex of the phallobase is distinctively shaped and somewhat protruding. *Oecetis mexicana* and *Oecetis angularis* both have the ventral lobe of the inferior appendage less protruding and not acutely angled. *Oecetis angularis* also differs by having the apex of the phallobase V-shaped in caudal view.

#### Adult.

Forewing length: male (7.8–8.5 mm), female (6.2–7.2 mm). Color generally pale yellowish-brown (darker than *Oecetis mexicana*), legs somewhat paler. Antennae whitish with indistinct, narrow annulations at intersection of antennomeres. Forewing spots small or very small; spots at base of discal and thyridial cells and base of fork V largest, these usually distinct and somewhat ovate, other spots small and indistinct; veins of forewing chord moderately spaced, usually with *s* and *r-m* relatively closely spaced, *m* more widely spaced; chord with small spots at juncture of major veins; apical spots, at apices of major veins, indistinct, but usually evident. Setae along veins in apical part of forewing elongate, prostrate, laterally diverging. Fringe of setae along costal margin of forewing dense, short, not strongly projecting.

#### Male genitalia.

Segment IX very short, with elongate setae along posterolateral margin. Tergum X with narrow, deflexed mesal lobe, lobe relatively elongate, nearly uniform in width, or slightly tapered apically, apex with small sensilla; lobe continuous basoventrally with short, paired lateral membranous projections. Preanal appendage moderately elongate, length 2 ½–3 times maximum width, simple in structure, apical setae elongate. Inferior appendage with rounded dorsal lobe and angularly projecting ventral lobe; ventral lobe elongate and strongly projecting, forming acute angle with dorsal lobe, posterior margin of ventral lobe, as viewed ventrally (Fig. 26E), with mesal margin only weakly bent near base; basomesal projection distinct, forming rounded projection with short, stiff setae; dorsal lobe with stout, mesally-curved setae on dorsal margin and stout, ventrally-curved setae on mesal surface. Phallobase, as viewed laterally, short, tubular, ventral margin rather strongly arched or curved, not angular, preapically with distinctive rugose wart (Fig. 26B); as viewed caudally, with apex U-shaped (Fig. 26C). Phallotremal sclerite prominent, basally forming short tubular collar, ventral margin projecting, apex acute; asymmetrical lateral sclerite present, usually on right side.

#### Holotype.

**Male** (pinned), **COSTA RICA: Guanacaste:** Hda. Tempisquito (Pelón de la Altura), 1 km NE km 265, rt. 1, 10°50.400'N, 85°33.600'W, el 100 m, 18.vii.1987, Holzenthal, Morse, Clausen (UMSP) (UMSP000064973).

#### Paratypes.

**MEXICO: Chiapas:** nr. Pijijiapan, 5.vii.1965, P.J. Spangler, 1 male, 11 females (alcohol) (NMNH); **Oaxaca:** Rancho San Pablo, 17 km E Tehuantepec, 23.v.1981, C.M. & O.S. Flint, Jr., 3 males, 2 females (pinned) (NMNH); nr. Tehuantepec, Rt. 190, km 808, 8–9.vi.1967, Flint & Ortiz, 1 male (pinned) (NMNH); **Sonora:** Alamos, 17.iii.1961, D. Beers, 2 males (alcohol) (NMNH); **Veracruz:** La Gloria Cardel, --i.1938, J. Camelo G., 1 male, 1 female (alcohol) (NMNH); **GUATEMALA:** Río Matapa, 10 km SE Esquintla, 5–6.iii.1970, E. J. Fee, 5 males, 3 females (alcohol) (NMNH); **Suchitepequez:** Río Sis, 22 km S Finca La Maquina, 11.vi.1966, O.S. Flint, 1 male (pinned), 10 males, 1 female (alcohol) (NMNH); **HONDURAS:** Nacaome, 4.viii.1967, O. S. Flint, 5 males (pinned), 34 males, 2 females (alcohol) (NMNH); El Zamorano, 16.iv.1966, G.F. Freytag, 1 male, 3 females (alcohol) (NMNH); St. Barb., Casas Viejas, el 250 m, 20.v.1966, J.M. Natta, 23 males, 8 females (alcohol) (NMNH); Pespire, 1.viii.1967, O.S. Flint, Jr., 2 males, 15 females (alcohol) (NMNH); Río Humuya, NW Comayagua, 3.viii.1967, O.S. Flint, Jr., 1 males, 4 females (alcohol) (NMNH); Comayagua, Hotel los Remos, south side Lago Yojoa, 15.xi.1987, L. Knutson, 2 males, 9 females (alcohol) (NMNH); **EL SALVADOR:** Quezaltepeque, 11.ii.1965, S. & W.D. Duckworth, 1 male, 3 females (alcohol) (NMNH); **NICARAGUA: Granada:** Isla Zapatera, Costa La Perra, 11°45.883'N, 85°50.974'W, el 52 m, 18.vii.2001, Chamorro, Ruiz, Martinez, 5 males, 1 female (pinned) (UMSP); Reserva Silvestre Privada Domitila, stream, 11°42.261'N, 85°56.948'W, el 79 m, 26.vii.2001, Chamorro & López, 1 male, 3 females (pinned) (UMSP); **Leon:** between La Leona & Izapa, 30.iv.1995, J.M. Maes, 1 male, 2 females (alcohol) (NMNH); **Rivas:** Río Las Lajas, ca. 2 km NW de El Genizaro, 11°21.53'N, 85°47.579'W, 1.vii.2000, L. Chamorro & J. Lacayo, 2 males (pinned) (UMSP); **COSTA RICA:** Río Seco, NW Esparta, 23.vii.1967, O.S. Flint, Jr., 3 males, 3 females (pinned), 1 male, 1 female (alcohol) (NMNH); 9 mi NW Esparta, 22.vii.1965, P.J. Spangler, 1 male, 2 females (alcohol) (NMNH); Las Canas, 13.vii.1965, P.J. Spangler, 8 males, 4 females (alcohol) (NMNH); Las Canas, Río Cordobicí, 26,vii.1967, O.S. Flint, Jr., 1 male (alcohol) (NMNH); **Guanacaste:** Colorado, 31.iii.1988, W. E. Steiner, J. M. Hill, J. M. Swearingen, J. M. Mitchell, 1 male (pinned) (NMNH); Parque Nacional Santa Rosa, Quebrada San Emilio, 10°51.720'N, 85°36.600'W, el 300 m, 27.vi.1986, Holzenthal, Heyn, Armitage, 1 male (pinned) (UMSP); Parque Nacional Santa Rosa, Río Poza Salada, 10°47.940'N, 85°39.120'W, el 10 m, 24.vii.1987, Holzenthal, Morse, Clausen, 1 male (pinned) (UMSP); Río Tempisquito, ca 3 km S route 1, 10°47.400'N, 85°33.120'W, el 75 m, 6.iii.1986, Holzenthal & Fasth, 14 males, 10 females (pinned), 240 males, 137 females (alcohol) (UMSP) (INBIO); Río Tizate, 7.2 km NE Canas Dulces, 10°46.380'N, 85°26.940'W, el 275 m, 28.vi.1986, Holzenthal, Heyn, Armitage, 1 male, 3 females (pinned), 6 males, 4 females (alcohol) (UMSP); same data as holotype, 6 males (pinned) 8 males, 2 females (alcohol) (UMSP).

#### Etymology.

This species is named *Oecetis verrucula*, as a diminutive of the Latin word *verruca*, a wart, and referring to the small preapical wart on the phallobase that is distinctive of this species.

### Species *incertae sedis*

*Oecetis marquesi* was probably placed by Chen (1993) in the *Oecetis avara* group because of the general shape of its inferior appendages. However, these are not exactly characteristic of the *Oecetis avara* group and also lack the primary character used to define the group: stout mesal setae on the dorsal lobe. The only other character Chen used to define the *Oecetis avara* group was the possession of “earlike” appendages on the female genitalia, as discussed and illustrated by Ross (1944) for *Oecetis avara*. While these are found in *Oecetis marquesi*, we are unsure about the overall utility of this character in defining the group, especially since similar appendages were also figured by Ross (1944) for *Oecetis eddlestoni*, which Chen placed in the subgenus *Quaria*. Other characters for placing the species are also ambiguous. *Oecetis marquesi* has the *s* and *r-m* veins of the forewing staggered, as in species of the *Oecetis avara* group, rather than linearly arranged, as in species of the *Oecetis punctata* group. As featured in the type description, a very short mesal lobe of tergum X is present, which would be typical of species of the *Oecetis avara* group; however, the character appears to be variable and was absent in 2 of 3 male specimens examined (including the one illustrated). The dorsal lobe of the inferior appendages has a mesally curved process with thickened setae, more or less typical of members of the *Oecetis punctata* group. Because of these character discrepancies, we have chosen to consider the species *incertae sedis* within the genus, pending a more thorough treatment of other species groups.

#### Oecetis marquesi

Bueno-Soria

http://species-id.net/wiki/Oecetis_marquesi

Fig. 27

Oecetis marquesi Bueno-Soria 1981: 113, fig. 7 [holotype male, MEXICO, Nuevo León, Monterrey, Potrero Redondo (IBUNAM)].

##### Diagnosis.

This species has a distinctive appearance, with a very angulate projection on the posterior margin of each of the inferior appendages (more or less conforming to a ventral lobe), that makes it unlikely that it could be confused with any other described species of New World *Oecetis*.

##### Adult.

Forewing length: male (9.8–10.2 mm), female (8.2 mm). Color brownish-yellow, legs pale yellowish. Antennae yellowish-white with indistinct, narrow annulations between segments. Forewings nearly immaculate, spots very small and indistinct; those at base of discal and thyridial cells and base of fork V generally evident (on close inspection). Veins of forewing chord widely spaced, *s* and *r-m* veins slightly closer; chord very weakly, not contrastingly, pigmented. Setae along veins in apical part of forewing elongate, semi-prostrate, laterally diverging. Fringe of setae along costal margin of forewing relatively short, dense, not conspicuously erect.

##### Male genitalia.

Segment IX very short, with elongate setae along posterolateral margin. Tergum X with or without narrow, deflexed mesal lobe; if present, very short, with several tiny apical sensilla; short, paired lateral membranous projections present in either case. Preanal appendage moderately elongate, length about 3 times maximum width, simple in structure, apical setae elongate. Inferior appendage dorsally projecting, with acute, angular, spine-like projection on posterior margin at past midlength; anterior margin with rounded, mesally-curved projection with short, stiff setae; mesal surface of appendage with fine setae, but without thickened, curved setae. Phallobase, as viewed laterally, short, tubular, moderately curved ventrally; apex, as viewed caudally, with ventral margin U-shaped. Phallotremal sclerite forming short tubular collar; asymmetrical lateral sclerite present on right side, very prominent and distinct.

**Figure 27.**
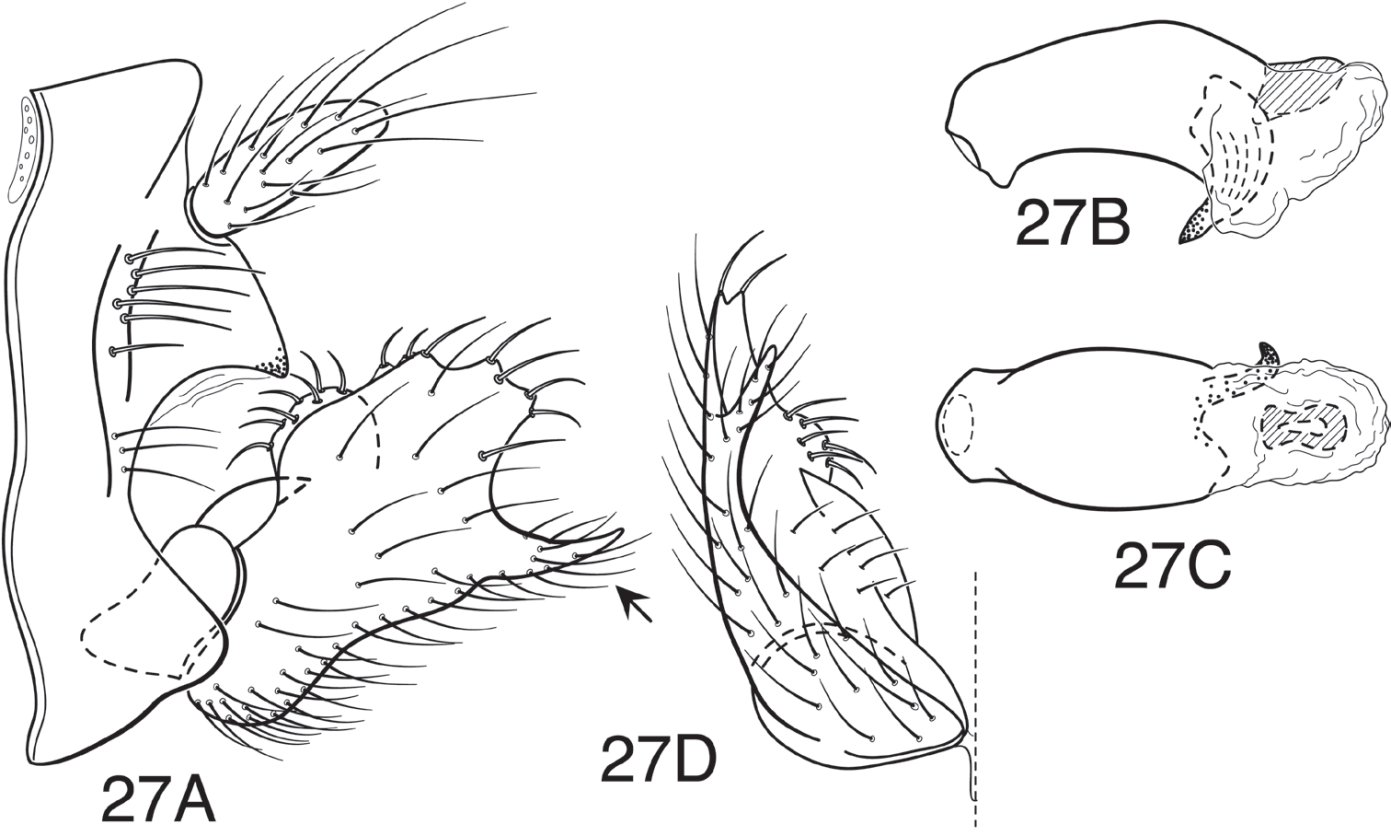
*Oecetis marquesi* Flint, male genitalia: **A** lateral **B** phallic apparatus, lateral **C** phallic apparatus, dorsal **D** inferior appendage, caudal.

##### Material examined.

**MEXICO: Chiapas:** 2.9 mi S Jitotal, 11.viii.1967, O.S. Flint, Jr., 1 male paratype (pinned) (NMNH); **Veracruz:** Rio Jamapa, 6 km N Coscomatepec, 2.v.1981, C.M & O.S. Flint, Jr., 1 male, 1 female (pinned) (NMNH); same locality, 26.v.1981, C.M. & O.S. Flint, Jr., 1 male (pinned) (NMNH).

### Undetermined *Oecetis avara* group material

***Oecetis avara* group material nr. *Oecetis houghtoni***

Figs 28, 45

**Figure 28.**
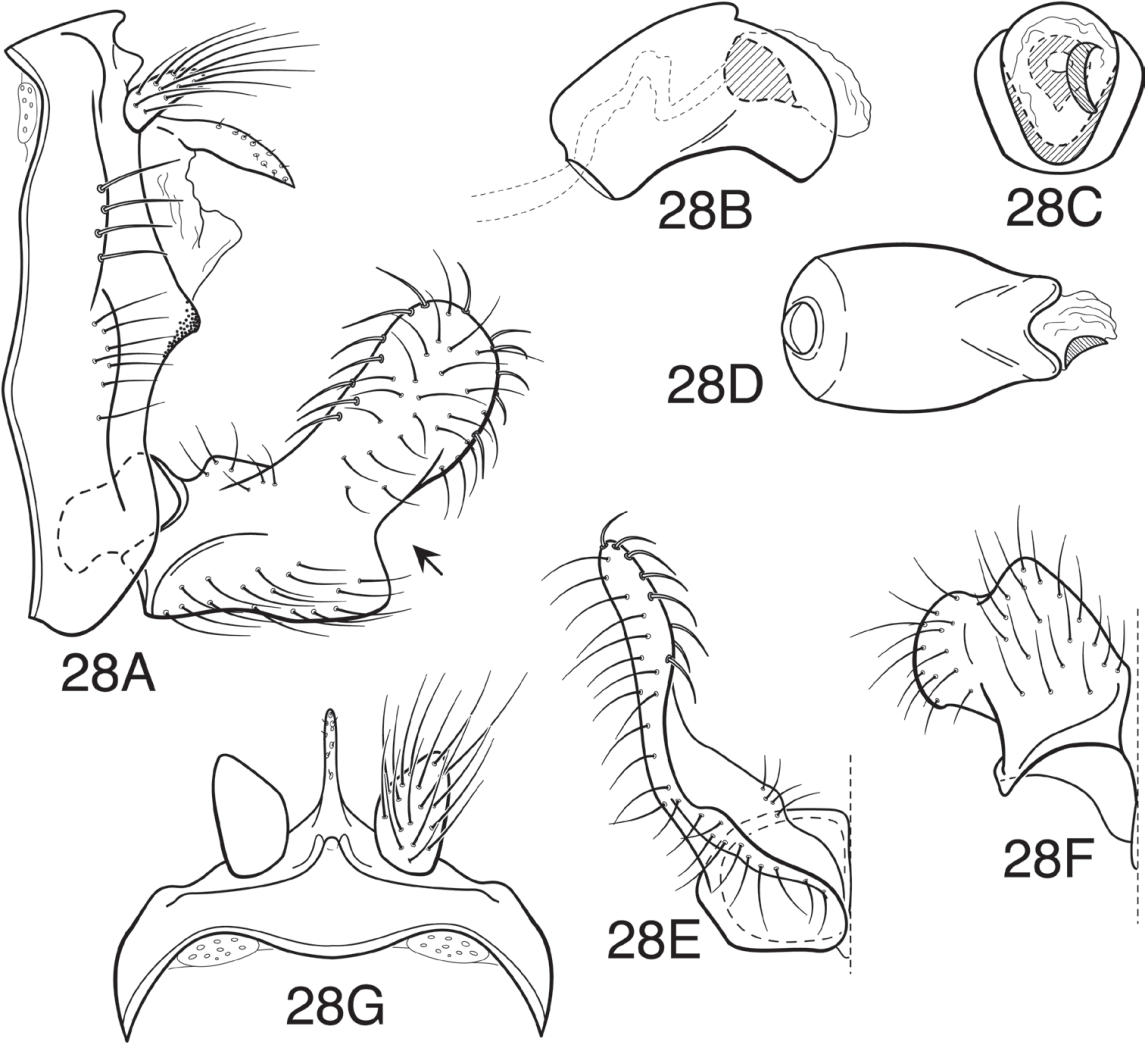
*Oecetis avara* group variant, nr. *Oecetis houghtoni*, Tennessee specimen: **A** lateral **B** phallic apparatus, lateral **C** phallic apparatus, caudal **D** phallic apparatus, ventral **E** inferior appendage, caudal **F** inferior appendage, ventral **G** segment IX and tergum X, dorsal.

**Diagnosis.** Some specimens from eastern United States and Canada differed from *Oecetis houghtoni* in having an obtusely angled ventral lobe of the inferior appendage and without a distinctly angled posterior margin of the dorsal lobe (Fig. 28A). These specimens vary somewhat in coloration and spacing of the chord of the forewing, but most have weak spotting on the forewing and narrowly spaced chord, as is typical of *Oecetis houghtoni*. Some of the specimens are darker than *Oecetis houghtoni*, while others, as for example those from Virginia, were lighter in color. Despite the color variation, there is little to distinguish the genitalia of these specimens. Figure 45 is of a dark colored specimen from Tennessee. No COI barcode sequences were obtained for this form. However, the cluster of sequences of *Oecetis* in the phylogram (Fig. 58) that were submitted from sources other than Minnesota (and which are slightly diverged from those of the Minnesota specimens of *Oecetis houghtoni*) may represent this form, since the geographic distribution of these specimens seems to be similar to those identified with this morphotype.

**Material examined. CANADA: New Brunswick:** Otter Bridge Rd., 15 mi W Renous, 46°52.5'N, 66°01.9'W, 16.vii.1996, C.M. & O.S. Flint, Jr., 1 male (NMNH); Canoose Str., 14 mi NW St. Stephen on Rt. 745, 45°22.4'N, 67°21.4'W, 28-30.vi.1996, C.M. & O.S. Flint, Jr., 1 male (NMNH); **USA: Maine:** Ashland, 31.vii.--, 1 male (NMNH); **Maryland:** Jackson’s Is. 22.vi.1902, H.S. Barber, 1 male (NMNH); **Tennessee:** Wayne Co., Buffalo R., Rt. 48, 45°N, 87.78°W, 14.vi.1992, C.M. & O.S. Flint, Jr., 1 male (NMNH); **Virginia:** Culpepper Co., Hazel River, Boston, 9-10.vii.1982, O.S. Flint, Jr., 3 males (NMNH); Hanover Co., South Anna River, falls at Rt. 657, 12.vi.1978, O.S. Flint, Jr., 1 male (NMNH); **West Virginia:** Pendleton Co., 5 mi SE Franklin, 27.vi.1968, J.F.G. & T.M. Clark, 1 male (NMNH); Pendleton Co., Smoke Hole Camp, 28–29.viii.1963, R. & O. Flint, 1 male (NMNH).

We examined a couple of specimens identified as *Oecetis avara* from the west coast of the United States (not figured) that were more or less similar in characters to the specimens from the eastern United States, as discussed above, in that the ventral lobe of the inferior appendages was relatively weakly developed and very obtusely angled. The phallobase in these specimens was even less strongly arched than in those from the East, and thus not at all like *Oecetis avara*. Additionally, the specimens were characterized by relatively dark wings with distinct, but diffuse spots, especially at the base of the discal and thyridial cells.

**Material examined.** **USA: California:** beside Klamath R., nr. Hamburg, 6.ix.1968, A.B. Gurney, 1 male (NMNH); **Oregon:** Baker Co., Baker, 2.viii.1967, J. H. Baker, 1 male (NMNH).

### Texan and southwestern material of *Oecetis avara* group

**Figures 29–32.**
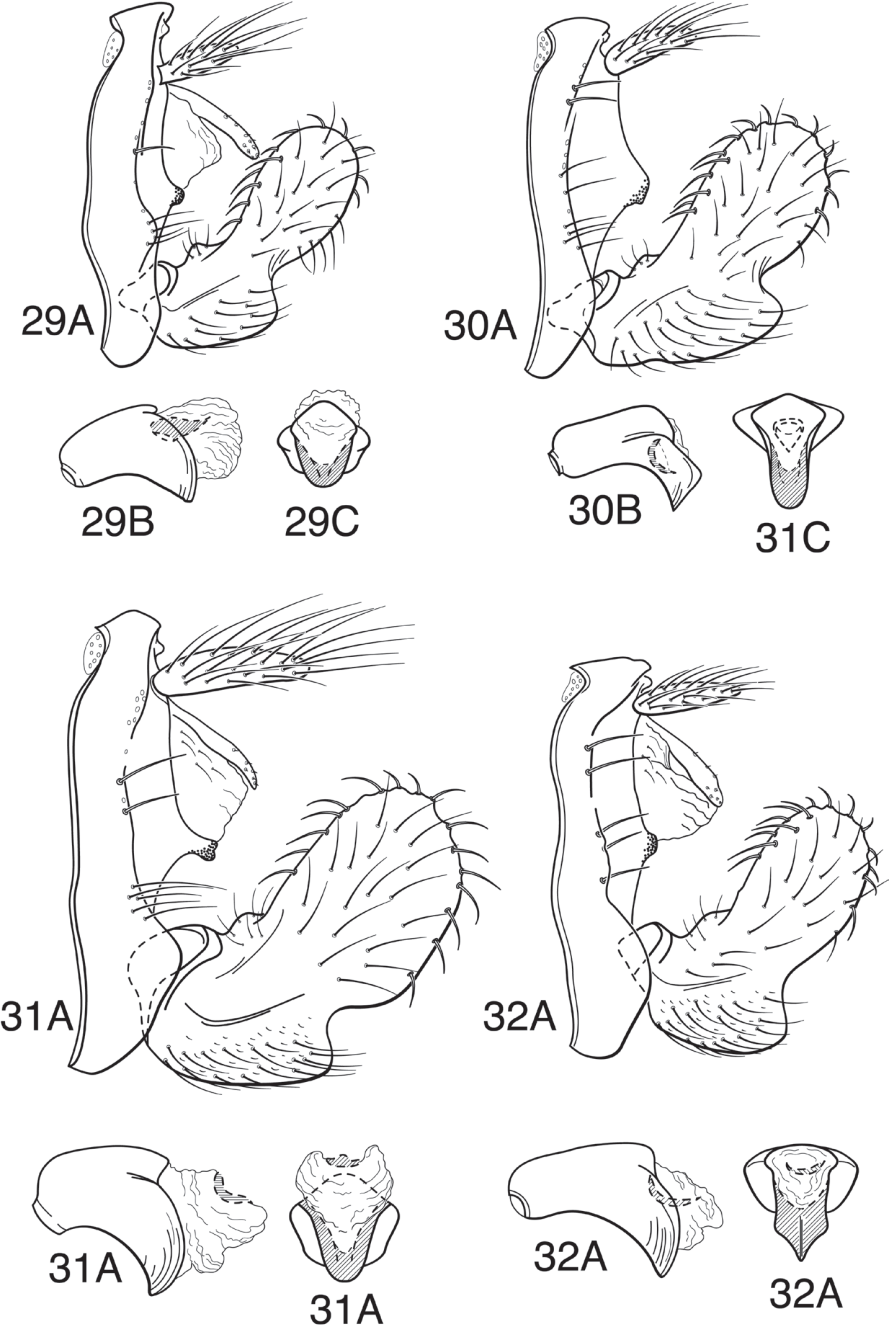
Forms and species with reduced or absent forewing spotting from SW United States. **29** *Oecetis variant* from Graham Co., Arizona **30** *Oecetis variant* from Huachuca Mts., Arizona **31** *Oecetis disjuncta* (Banks), California **32** *Oecetis apache* sp. n., New Mexico: **A** lateral **B** phallic apparatus, lateral C phallic apparatus, caudal.

Material identified as *Oecetis avara* from Texas, USA, through the southwest is especially variable. Most of the specimens are light in color. Spotting on the forewing is prominent in most specimens examined from Texas, as in *Oecetis avara*, but genitalic characters are very intermediate between *Oecetis avara* and *Oecetis houghtoni*. In the southwest, the spotting on the forewing seems to become smaller, or less evident, or even absent in some localities. Figures 29–32 show specimens from Arizona, New Mexico, and California, drawn to the same scale. All of these are approximately the same yellowish color and have spotting on the forewing either absent or greatly reduced. Figures 31 and 32 represent *Oecetis disjuncta* from California, and *Oecetis apache* sp. n. from New Mexico, respectively. Figures 29 and 30 are from Arizona and probably represent 2 additional species, neither of which is readily identified as *Oecetis houghtoni* or *Oecetis avara*. However, they have the same relative character differences: ventral lobe of the inferior appendages either weakly or strongly developed, and phallobase either weakly or more strongly curved. The character development is more extreme than that in either *Oecetis houghtoni* or *Oecetis avara*. As alternative interpretations of the data, we consider the possibilities that these may either represent regional variants of those species or additional new species.

### COI barcode data

A number of specimens in the *Oecetis avara* group were submitted to the Barcode of Life (BoL) initiative (Guelph, Ontario, Canada) from the University of Minnesota Insect Collection (UMSP). Some species were not submitted because they were represented by only a few specimens (*Oecetis acciptrina* sp. n.; *Oecetis agosta* sp. n.; *Oecetis maritza* sp. n.; and *Oecetis tumida* sp. n.) or were not represented by specimens in the UMSP collection (*Oecetis campana* sp. n., *Oecetis elata* Denning & Sykora, and *Oecetis marquesi* Bueno-Soria). Other species were submitted but did not yield any sequence data (*Oecetis angularis* sp. n.; *Oecetis mexicana* sp. n.; and *Oecetis uncata* sp. n.). In total, about half of the species of the *Oecetis avara* group produced COI barcode sequences, or partial barcode sequences. Several species of the *Oecetis punctata* group were also included as outgroup taxa. Specimens identified only as *Oecetis* were submitted to the BoL initiative from sources other than the UMSP collection and were originally identified as *Oecetis avara*. The specimens were not examined during the study and the sequences are included for whatever value they add to the overall study. The phylogram presented (Fig. 58) is a NJ (neighbor joining) construct of the available taxa produced by using a K2P model (as generally recommended by the Barcode of Life initiative and representing a default method of analysis). It should be noted that taxonomic conclusions were based on morphological, rather than molecular, criteria. The phylogram is presented for the ancillary value it adds to the paper. In general, the phylogram is useful in corroborating the taxonomic conclusions reached, with sequences for the available species clustering together and separated from other species by moderate barcode distances. Because of the limited sequence data available, a more rigorous analysis of the data was not justified. We would caution against an over interpretation of the phylogram; in particular, it should not be construed as a phylogeny of the taxa.

### Key to *Oecetis avara* group males

For the most part, the most diagnostic characters for identifying species in the *Oecetis avara* group are related to the structure of the phallobase and inferior appendages. Forewing markings, coloration, and size, while providing useful adjunct characters, are less reliable. While color and size characters are sometimes used in the key, it should be kept in mind that they were based on specimens examined and may not reflect the total variation for a species. Distributions given are based on the known ranges of the species. The most relevant genitalic characters for identifying the various species are pointed out with small arrows in the accompanying illustrations and discussed in the diagnoses. The character considered most diagnostic for a given species is the one listed 1st in a couplet; additional characters are considered ancillary. However, all of the characters in the key should be considered before a determination is made and species indentified in the key should be closely compared to illustrations and also against the diagnoses provided for the individual species. Even so, it is likely that considerable difficulty will be experienced by the novice in identifying species until the subtle differences between them are fully appreciated.

**Table:** 

1	Ventral lobe (posterior projection) of inferior appendage with apex very acute (Fig. 27A); dorsal lobe of inferior appendages without stout setae on mesal surface (Fig. 27D); mesal process of tergum X very short or absent (Mexico) (Species *incertae sedis*)	*Oecetis marquesi* Bueno-Soria
–	Ventral lobe of inferior appendage with apex rounded or subacute (Figs 5A, 8A); dorsal lobe of inferior appendages with stout setae on mesal surface (Fig. 5E); mesal process of tergum X present (except in *Oecetis elata*) (*Oecetis avara* group)	2
2(1)	Phallobase with apex distinctly V-shaped in caudal view (Fig. 3C) (ventral margin usually at least somewhat “keeled” preapically)	3
–	Phallobase with apex U-shaped in caudal view (Fig. 5C) (apex never “keeled” preapically, but sometimes rather narrowly U-shaped; if in doubt, follow this couplet)	7
3(2)	Size very small (forewing less than 6 mm); apex of phallobase sharply downturned (Fig. 1B) (Costa Rica, Panama, Ecuador)	*Oecetis acciptrina* sp. n.
–	Size larger (forewing more than 8 mm); phallobase either less downturned (Fig. 3B) or curvature more nearly in middle (Figs 4B, 16B, and 25B)	4
4(3)	Phallobase moderately elongate and arched ventrally, not strongly bent (Fig. 3B); Color yellowish with distinct spots; inferior appendage with ventral lobe weakly projecting, but very angular (Guatemala to Ecuador)	*Oecetis angularis* sp. n.
–	Phallobase more angularly bent (Figs 4B, 16B, and 25B); Color darker, yellowish-brown or light brown (spots small or absent in *Oecetis metlacensis* Bueno-Soria and *Oecetis apache* sp. n., distinct only in *Oecetis uncata* sp. n.), inferior appendage with ventral lobe more rounded, projecting or not)	5
5(4)	Ventral lobe of inferior appendage separated from dorsal lobe by shallow emargination, very weakly projecting (Fig. 4A); forewings without evident spots (Arizona, New Mexico)	*Oecetis apache* sp. n.
–	Ventral lobe of inferior appendage more evidently projecting (Figs 16A, 17A, 25A); forewings with wing spots, spots either distinct or small and indistinct (Mexico, Costa Rica)	6
6(5)	Ventral lobe of inferior appendage, and articulation with dorsal lobe, distinctly angular (Fig. 25A); inflection of phallobase more apical (Fig. 25B); forewings with distinct spots (Costa Rica)	*Oecetis uncata* sp. n.
–	Ventral lobe of inferior appendage rounded, less evidently projecting, separated from dorsal lobe by deep emargination (Figs 16A, 17A); Phallobase more nearly angled in middle (Figs 16B, 17B) (angle sharp or not); forewings with small and relatively indistinct wing spots (Mexico, Costa Rica)	*Oecetis metlacensis* Bueno-Soria
7(2)	Ventral margin of phallobase, as viewed laterally, at least somewhat convexly bulged preapically (Figs 2B, 22B, 24B); color yellowish	8
–	Ventral margin of phallobase, as viewed laterally, either arched (Fig. 5B) or angular (Fig. 23B), rarely nearly straight (Fig. 6B); color yellowish to dark brown	10
8(7)	Phallobase relatively short and bulbous apically (Fig. 24B); inferior appendage with ventral lobe very weakly, but angularly, projecting, forming obtuse or right angle with dorsal lobe (Fig. 24A)	*Oecetis tumida* sp. n.
–	Phallobase typically more elongate, projecting (Figs 2B, 22B); inferior appendage with ventral lobe distinctly projecting, forming acute angle with dorsal lobe. (Fig. 22B)	9
9(8)	Phallobase, in lateral view, very angularly bent or elbowed in middle (Fig. 2B); inferior appendage with dorsal lobe very wide, ventral lobe very distinctly projecting, forming very acute angle with dorsal lobe (Fig. 2A); ventral lobe not angularly bent as viewed ventrally (Fig. 2E); Forewing with wing spots absent or very indistinct (Mexico)	*Oecetis agosta* sp. n.
–	Phallobase, in lateral view, arched and protruding, but not angularly bent (Fig. 22B); inferior appendage with dorsal lobe less wide and ventral lobe less projecting (Fig. 22A); ventral lobe very angularly bent as viewed ventrally (Fig. 22E); Forewing with very conspicuous wing spots (Mexico to Ecuador)	*Oecetis protrusa* sp. n.
10(7)	Apex of phallobase (caudal view), very diagnostically constricted or “pinched” (Figs 7C, 7D); ventral lobe of inferior appendage forming obtuse angle with dorsal lobe (Fig. 7A); size small (forewing length less than 8 mm); color yellowish, forewing spots distinct, rounded (Mexico to northern South America)	*Oecetis constricta* sp. n.
–	Apex of phallobase various, but not strongly constricted (if narrowly U-shaped, then with evident protruding asymmetrical phallic sclerite) (Fig. 19C); ventral lobe of inferior appendage acute or obtuse; size small to large; color and forewing spotting various	11
11(10)	Ventral lobe of inferior appendage separated from dorsal lobe by rounded emargination, apex of lobe not angularly projecting (Figs 19A, 23A)	12
–	Ventral lobe of inferior appendage angularly projecting, angle varying from obtuse to acute (Figs 6A, 26A)	14
12(11)	Very small (forewing less than 8 mm) and dark brown in color; forewing spots absent (Mexico)	*Oecetis elata* Denning & Sykora
–	Larger in size (forewing greater than 9.5 mm); if dark brown, then with evident pigmented chord and small forewing spots	13
13(10)	Phallobase strongly downturned, ventral apex with distinct warty or rugose texture, often forming raised wart (Figs 23B, 23D); wings dark brown with distinctly pigmented forewing chord and indistinct forewing spots; size larger (forewing greater than 10.5 mm) (Mexico and western U.S.A)	*Oecetis sordida* sp. n.
–	Phallobase strongly downturned, ventral apex without warty texture (Fig. 8B); wings lighter in color (brown or yellowish-brown), chord lightly pigmented or not; size smaller (forewing less than 10.5 mm) (known distribution – California, Oregon)	*Oecetis disjuncta* (Banks)
14(11)	Endotheca without asymmetrical phallic sclerite (but with distinct phallotremal sclerite) (lower Central America)	15
–	Endotheca with asymmetrical phallic sclerite (visible in caudal view, usually on right side) (Figs 5C, 5D), in addition to usual phallotremal sclerite (widespread from North America to South America)	16
15(14)	Apex of phallobase, in caudal view, distinctly wide (Fig. 21C); color yellowish, forewing spots small or irregular in shape (Guatemala, Nicaragua)	*Oecetis patula* sp. n.
–	Apex of phallobase, in caudal view, more narrowly U-shaped (Fig. 15C); color light brown, forewing spots distinct, but weakly contrasting with color of wing; size very small (ca. 6 mm) (Costa Rica)	*Oecetis maritza* sp. n.
16(14)	Ventral margin of phallobase with small, preapical rugose wart (Fig. 26B); ventral lobe of inferior appendage distinctly projecting and forming acute angle with dorsal lobe; spots of forewings relatively small, oval (Mexico to Costa Rica)	*Oecetis verrucula* sp. n.
–	Ventral margin of phallobase without preapical rugose wart; ventral lobes of inferior appendage and forewing spots variable	17
17(16)	North America (to northern Mexico); yellowish or brownish species, with setae on costal margin of forewing not distinctly projecting; forewing spots relatively small, distinct or not	18
–	Central and South America; yellowish species, with setae of costal margin of forewing distinctly projecting; forewing spots distinct and rounded	20
18(17)	Ventral margin of inferior appendages very distinctly projecting; ventral lobe forming very acute angle with dorsal lobe (angle with noticeable wrinkles); apex of phallobase more distinctly downturned and heavily sclerotized; color usually lighter, yellowish or yellowish-brown with distinct forewing spots; forewing chord with crossveins relatively widely spaced	*Oecetis avara* (Banks)
–	Ventral margin of inferior appendages less distinctly projecting; ventral lobe forming slightly acute to obtuse angle with dorsal lobe (angle usually somewhat rounded); apex of phallobase less strongly downturned and less sclerotized; color usually darker, light brown with forewing spots small and indistinct; forewing chord with crossveins relatively narrowly spaced (Fig. 34A)	19
19(18)	Ventral lobe of inferior appendage forming slightly acute or approximately right angle to dorsal lobe; posterior margin of dorsal lobe rather distinctly angulate (Fig. 10A)	*Oecetis houghtoni* sp. n.
–	Ventral lobe of inferior appendage forming distinctly obtuse angle to dorsal lobe (Fig. 28A); posterior margin of dorsal lobe straight or evenly rounded (as in *Oecetis avara*), not distinctly angulate	eastern USA variant (unplaced to species)
20(17)	Ventral margin of phallobase nearly straight or very weakly arched (Fig. 6B); forewing spots very large and distinct; setae on costal margin of forewing very elongate and projecting	*Oecetis campana* sp. n.
–	Ventral margin of phallobase more distinctly arched (Figs 18B, 19B); forewing spots distinct, but only moderately large; setae on costal margin of forewing projecting, but not as dense or elongate	*Oecetis mexicana* sp. n.

**Figures 33–34.**
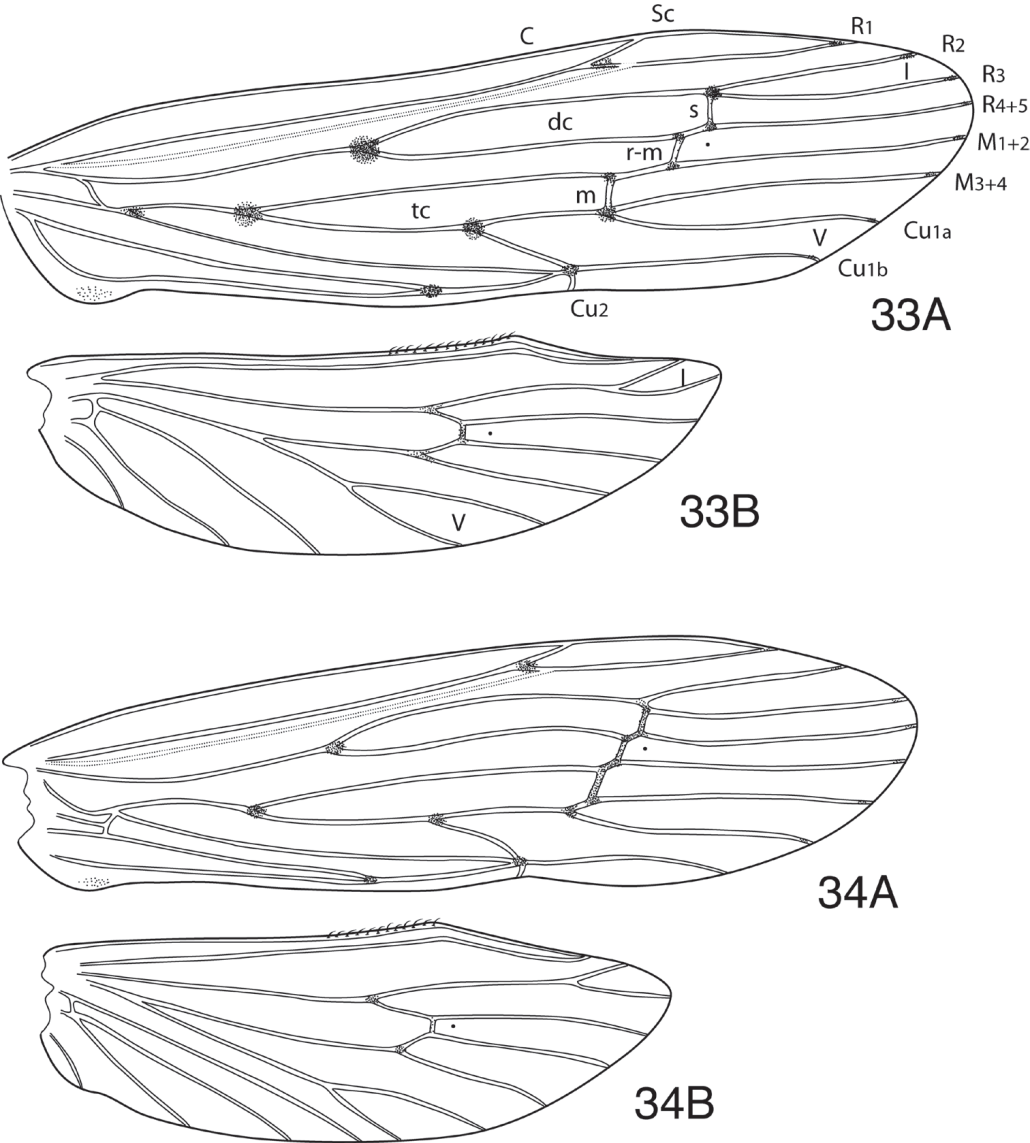
Wings of *Oecetis avara* group species. 33 *Oecetis angularis* sp. n. 34 *Oecetis houghtoni* sp. n.: **A** forewing B hind wing.

**Figures 35–45.**
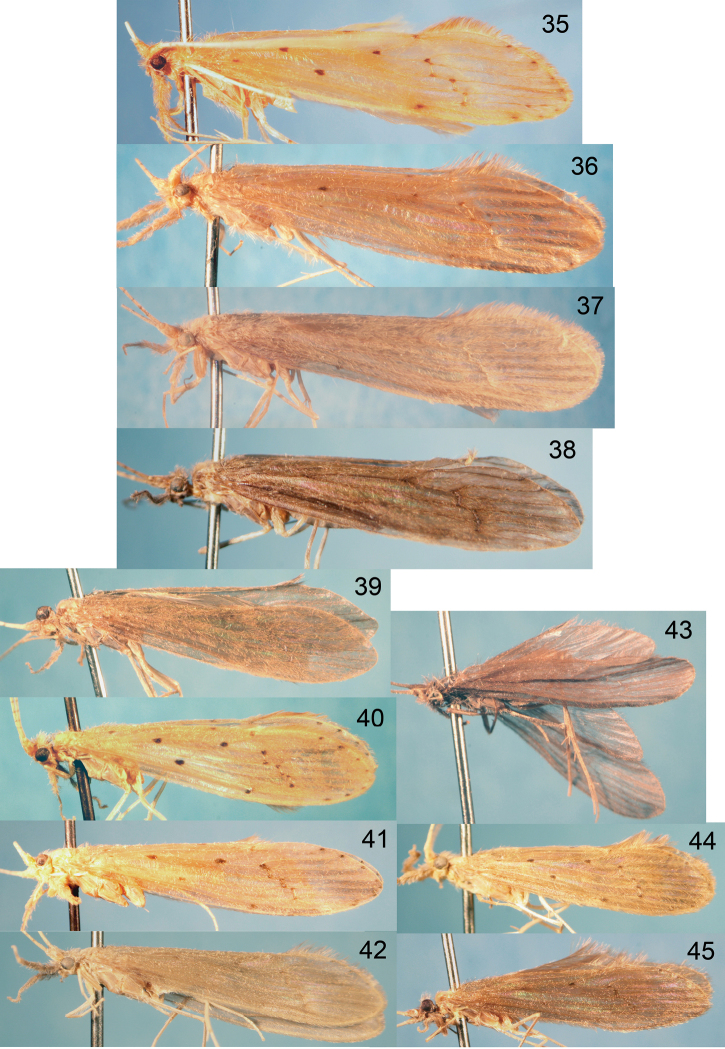
*Oecetis avara* group species – lateral habitus (male). **35** *Oecetis uncata* sp. n. **36** *Oecetis metlacensis* Bueno-Soria **37** *Oecetis apache* sp. n. **38** *Oecetis sordida* sp. n. **39** *Oecetis disjuncta* (Banks) **40** *Oecetis avara* (Banks) – Florida **41** *Oecetis avara* (Banks) – Minnesota **42** *Oecetis houghtoni* sp. n. **43** *Oecetis elata* Denning & Sykora 44 *Oecetis verrucula* sp. n. **45** *Oecetis* species nr. houghtoni – Tennessee.

**Figures 46–45.**
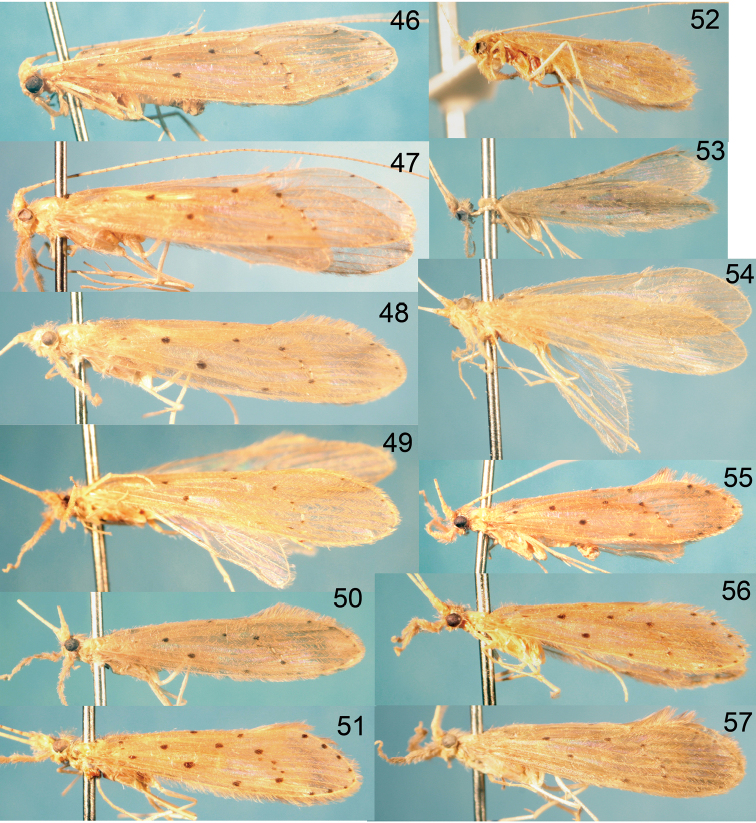
*Oecetis punctata* and *Oecetis avara* group species – lateral habitus (male). **46** *Oecetis punctata* Navás **47** *Oecetis angularis* sp. n. **48** *Oecetis tumida* sp. n. **49** *Oecetis patula* sp. n. **50** *Oecetis mexicana* sp. n. **51** *Oecetis campana* sp. n. **52** *Oecetis acciptrina* sp. n. (female) **53** *Oecetis maritza* sp. n. **54** *Oecetis agosta* sp. n. **55** *Oecetis protrusa* sp. n. **56** *Oecetis constricta* sp. n. **57** *Oecetis verrucula* sp. n.

**Figure 58.**
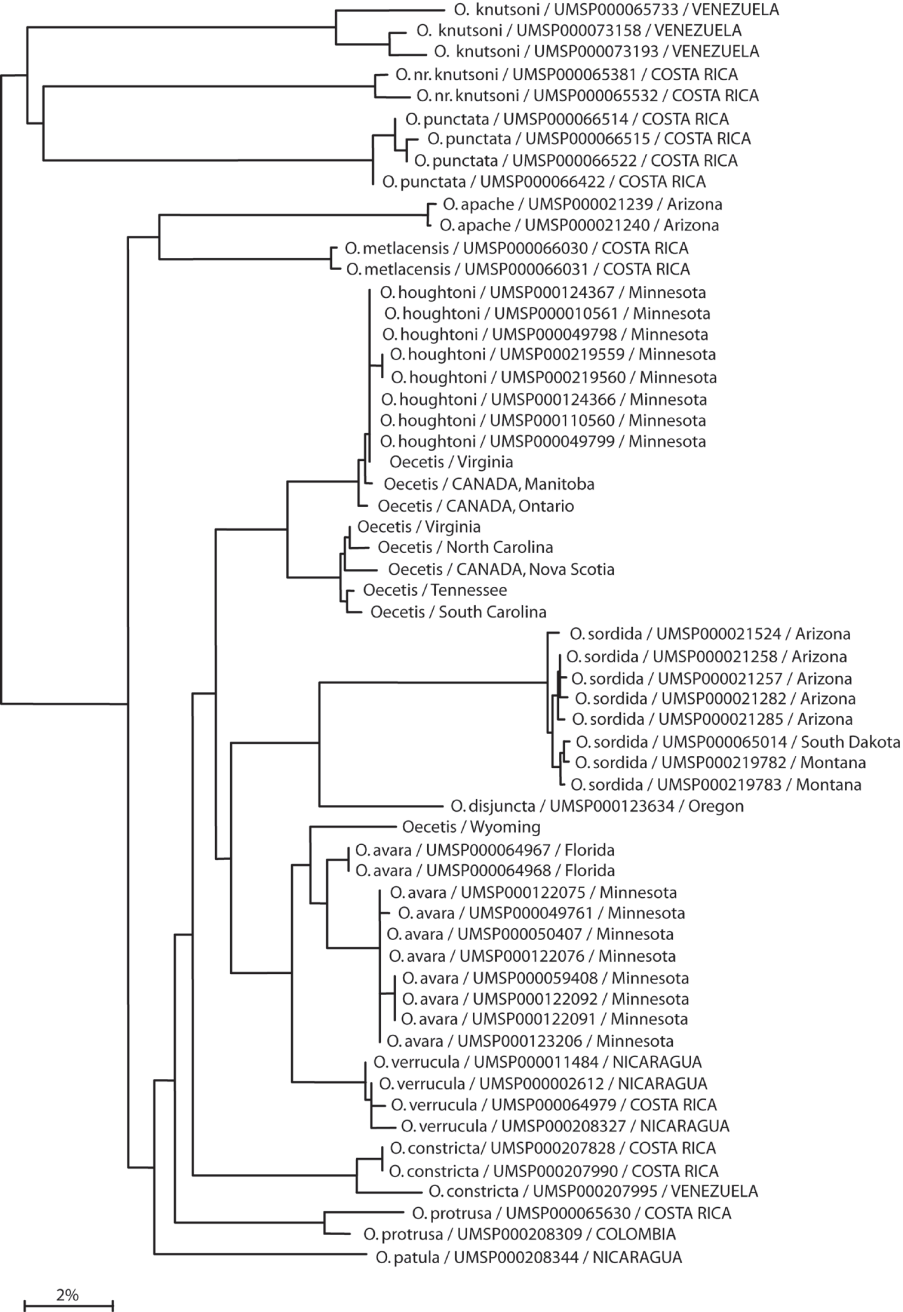
NJ phylogram of COI barcode sequences for *Oecetis avara* group species.

## Supplementary Material

XML Treatment for Oecetis acciptrina

XML Treatment for Oecetis agosta

XML Treatment for Oecetis angularis

XML Treatment for Oecetis apache

XML Treatment for Oecetis avara

XML Treatment for Oecetis campana

XML Treatment for Oecetis constricta

XML Treatment for Oecetis disjuncta

XML Treatment for Oecetis elata

XML Treatment for Oecetis houghtoni

XML Treatment for Oecetis maritza

XML Treatment for Oecetis metlacensis

XML Treatment for Oecetis mexicana

XML Treatment for Oecetis patula

XML Treatment for Oecetis protrusa

XML Treatment for Oecetis sordida

XML Treatment for Oecetis tumida

XML Treatment for Oecetis uncata

XML Treatment for Oecetis verrucula

XML Treatment for Oecetis marquesi

## Appendix

Distribution of *Oecetis avara* group species. (doi: 10.3897/zookeys.376.6047.app) File format: WinZip archive of Google Earth Keyhole Markup files (zip, kmz).

**Explanation note:** **Map 1.** [Square] *Oecetis angularis* sp. n.; [Circle] *Oecetis protrusa* sp. n.; [Triangle] *Oecetis tumida* sp. n.

**Map 2.** Legend: [Star] *Oecetis apache* sp. n.; [Circle] *Oecetis avara* (Banks); [Diamond] *Oecetis disjuncta* (Banks); [Square] *Oecetis houghtoni* sp. n.; [Triangle] *Oecetis sordida* sp. n.

**Map 3.** Legend: [Square] *Oecetis acciptrina* sp. n.; [Circle] *Oecetis agosta* sp. n.; [Diamond] *Oecetis elata* Denning & Sykora; [Star] *Oecetis maritza* sp. n.; [Triangle] *Oecetis patula* sp. n.

**Map 4.** Legend: [Square] *Oecetis campana* sp. n.; [Diamond] *Oecetis constricta* sp. n.; [Circle] *Oecetis mexicana* sp. n.

**Map 5.** Legend: [Square] *Oecetis metlacensis* Bueno-Soria; [Circle] *Oecetis uncata* sp. n.; [Diamond] *Oecetis verrucula* sp. n.
